# Renal injury phenotypes with ICI–VEGFR-TKI combinations in renal cell carcinoma: active-comparator analyses in FAERS and JADER

**DOI:** 10.3389/fimmu.2026.1971413

**Published:** 2026-10-14

**Authors:** Tiejun Li, Liuyue Yang, Xiangbiao He, Kaixiang Wang, Guihua Cao

**Affiliations:** 1 Department of Urology, The People’s Hospital of Leshan, Leshan, China; 2 Department of Anesthesiology, The People’s Hospital of Leshan, Leshan, China

**Keywords:** acute kidney injury, FAERS, immune checkpoint inhibitor, JADER, machine learning, pharmacovigilance, renal cell carcinoma, VEGFR tyrosine kinase inhibitor

## Abstract

**Background:**

Renal adverse-event reports during immune checkpoint inhibitor (ICI)–VEGFR tyrosine kinase inhibitor (TKI) therapy may combine immune-interstitial, proteinuric, and nonspecific acute kidney injury (AKI) features. We compared their reporting patterns with those of nivolumab plus ipilimumab in renal cell carcinoma (RCC).

**Methods:**

We analyzed FAERS/AERS (2004Q1–2026Q2) and the JADER 202607 release (through 2026Q1), applying latest-version handling, strict indication–drug linkage, author-defined Tier-A reporting labels, regimen-specific common support, and year-stratified Mantel–Haenszel reporting odds ratios (MH-RORs). JADER supplied cross-database directional corroboration. Secondary classification of the rule-derived labels used non-anchor features, with and without regimen indicators, and included temporal evaluation in 2024–2025. Additional analyses assessed sparse cells, terminal periods, standard renal queries, and model ablations.

**Results:**

Among 84,369 strict-RCC FAERS reports, 2,307 met Tier-A definitions: 219 immune-interstitial, 1,076 VEGF-axis, 932 nonspecific AKI, and 80 mixed. For pembrolizumab plus axitinib, pembrolizumab plus lenvatinib, and nivolumab plus cabozantinib, VEGF-axis MH-RORs were 5.31 (95% CI 2.43–11.59), 19.48 (9.03–42.01), and 4.59 (2.07–10.19). Proteinuria dominated this signal. Exclusive Tier-A AKI estimates were 1.05, 0.93, and 0.78, with intervals crossing one; this finding was definition-dependent, as a separate narrow acute-renal-failure query yielded 1.56 (1.20–2.02) for pembrolizumab plus lenvatinib. Excluding 2026 retained the primary VEGF-axis direction. JADER estimates were 6.28, 19.27, and 6.16. In a secondary consistency analysis, adding regimen increased temporal macro-AUPRC from 0.606 to 0.638, but immune-interstitial class average precision remained 0.253.

**Conclusions:**

The reproducible finding is a proteinuria-dominant VEGF-axis reporting pattern, not a validated renal mechanism or comparative incidence estimate. Differential surveillance, sparse comparator events, phenotype misclassification, and shared reporting ecology constrain interpretation. The classifier describes internal label consistency and has no established diagnostic use.

## 1 Introduction

Kidney cancer caused more than 430,000 new cases and 179,000 deaths worldwide in 2020 (1). Systemic treatment for advanced clear-cell renal cell carcinoma (RCC) has changed rapidly. Current guidelines recommend immune checkpoint inhibitor (ICI)-based combinations as first-line standards. Treatment selection considers International Metastatic RCC Database Consortium risk, disease tempo, comorbidity, therapeutic goals, and patient preference (2, 3). Improved survival has introduced a practical attribution problem. Adverse events may arise from either component of a combination, yet attribution guides treatment interruption, glucocorticoid use, and the need for kidney biopsy.

Phase III trials established the principal first-line combinations. Nivolumab plus ipilimumab improved outcomes over sunitinib, as did pembrolizumab plus axitinib, nivolumab plus cabozantinib, and pembrolizumab plus lenvatinib (4–7). Extended follow-up confirmed sustained efficacy for each regimen (8–11). Network evidence further supports ICI-based combinations in untreated advanced RCC (12). Clinicians therefore manage overlapping immune-mediated and antiangiogenic toxicities in patients with several competing causes of renal dysfunction. These include reduced nephron mass, chronic kidney disease, hypertension, diabetes, obstruction, infection, volume depletion, and nephrotoxic concomitant medicines.

Renal toxicity is not a single clinical entity. Meta-analysis has documented nephrotoxicity with ICI combination treatment in RCC (13), but the label “AKI” does not specify the cause. ICI-associated kidney injury most often raises concern for acute tubulointerstitial nephritis. Glomerular lesions, electrolyte disorders, and other pathologies also occur (14–19). Serum creatinine kinetics and routine urinalysis may not reliably separate immune-mediated nephritis from hemodynamic, septic, obstructive, or drug-related AKI. Misclassification can prompt unnecessary immunosuppression or interruption of effective therapy. Conversely, delayed recognition of immune-mediated injury may allow further renal damage.

VEGF-pathway inhibition produces a distinct renal pattern. Hypertension and proteinuria are recognized class effects. Nephrotic syndrome and thrombotic microangiopathy can reflect disruption of podocyte–endothelial VEGF signaling (20). Mechanistic reviews describe endothelial dysfunction, glomerular barrier injury, and potential interactions between VEGF inhibition and immune activation (21). Clinicopathologic series have also characterized VEGF–VEGFR2 inhibitor-associated glomerular microangiopathy (22). A creatinine increase during combination therapy may therefore accompany immune-interstitial, proteinuric/endothelial, or nonspecific AKI features. These patterns require different diagnostic and management approaches.

Pharmacovigilance comparisons can mix disease context, treatment era, co-therapy, and dissimilar reporting backgrounds. Within-RCC active comparisons narrow this question but cannot recover treated-patient denominators or unmeasured clinical characteristics. Classification of report-derived labels poses a separate problem: label-defining terms can re-enter as predictors, and remaining features may still encode reporting practice rather than biology. A second reporting database can test directional consistency, but it does not establish independent patients or clinical validity.

We compared three RCC ICI–VEGFR-TKI regimens with nivolumab plus ipilimumab using prespecified, mutually exclusive immune-interstitial, VEGF-axis, and nonspecific AKI reporting phenotypes, alongside mixed reports. The primary question concerned relative reporting enrichment. Secondary modeling asked whether non-anchor co-reports and regimen indicators could recover these author-defined labels. JADER supplied cross-database directional corroboration. The analyses were not designed to estimate incidence, isolate a TKI add-on effect, or determine individual renal pathology.

## 2 Materials and methods

### 2.1 Study design, data governance, and reporting framework

This retrospective study used deidentified spontaneous adverse-event reports from FAERS/AERS and JADER. The study was not preregistered. References to prespecified elements concern documented analysis specifications, not a publicly registered protocol. These specifications covered the phenotype dictionary, comparator, common-support windows, machine-learning features, model hierarchy, validation windows, multiplicity control, and interpretation rules; *post-hoc* sensitivity analyses are identified separately in *Section 2.9*. Reporting followed the READUS-PV statement and its explanation for disproportionality analyses based on individual case safety reports (23, 24). Both databases contain deidentified public data. Institutional review board approval and informed consent were therefore not required.

FAERS/AERS quarterly files covered 2004Q1 through 2026Q2, comprising 90 quarters. We assembled report versions globally, applied FDA deletion files, and retained one latest version per CASEID using FDA receipt date and PRIMARYID order. The prespecified target-program candidate screen contained 574,338 unique latest CASEIDs, not the entire FAERS database. JADER used the official 202607 release identifier, not a July 2026 observation endpoint; its CP932-encoded four-table files covered 2004Q1–2026Q1 and contained 1,043,485 unique DEMO report identifiers, with no orphan DRUG or REAC identifiers. Only 37 reports carried the terminal 2026Q1 label. Neither terminal release was assumed mature. A *post-hoc* sensitivity analysis excluded all 2026 report-year strata; this tests terminal-period influence but does not recover delayed reports or reproduce an earlier database vintage.

### 2.2 Strict RCC treatment context and regimen construction

Eligibility required an RCC indication linked to a target drug on the same drug-sequence record. This restriction reduced heterogeneity in indication and treatment context. In FAERS, we used the DRUG_SEQ–INDI_DRUG_SEQ linkage rather than report-level co-occurrence; both regimen components had to be recorded as primary or secondary suspects, and at least one target drug required a same-sequence RCC indication link. In JADER, indication is recorded on the DRUG row, and both regimen components had to be primary suspects in the main analysis. Indication terms were mapped to a prespecified RCC dictionary containing renal cell carcinoma and reviewed synonymous kidney cancer concepts. The broader ML parent cohort required a same-sequence RCC link to at least one suspect pembrolizumab, nivolumab, ipilimumab, axitinib, lenvatinib, cabozantinib, or sunitinib record, and did not require membership in a named doublet.

The targets were pembrolizumab plus axitinib, pembrolizumab plus lenvatinib, and nivolumab plus cabozantinib. Nivolumab plus ipilimumab provided a contemporary RCC ICI-only reporting comparator. Disease matching did not establish matching on treatment line, IMDC risk, histology, or prior treatment, which were unavailable. Comparing PD-1 plus TKI with PD-1 plus CTLA-4 blockade does not isolate the effect of adding a TKI. Reports matching multiple named regimens were excluded from the primary pharmacovigilance cohorts. Sunitinib remained a historical/mechanistic sensitivity comparator, not a surveillance-calibrated control.

Common support began at regimen-specific RCC availability. FAERS support began in 2019Q2 for pembrolizumab plus axitinib, 2021Q3 for pembrolizumab plus lenvatinib, and 2021Q1 for nivolumab plus cabozantinib. JADER support was established empirically within the Japanese system. Start dates were 2019Q4, 2022Q1, and 2021Q3, respectively. For each comparison, we reconstructed the nivolumab plus ipilimumab denominator over the same support period. Comparator counts consequently differed among comparisons.

### 2.3 Renal phenotype definitions

Tier-A is an author-defined exact-term grouping, not an official MedDRA grade, a single standardized MedDRA query (SMQ), or a validated etiologic diagnosis. The immune-interstitial reporting phenotype included tubulointerstitial nephritis, immune-mediated nephritis, autoimmune nephritis, nephritis autoimmune, and nephritis interstitial. The VEGF-axis reporting phenotype included proteinuria, nephrotic syndrome, and renal-limited thrombotic microangiopathy; the nonspecific AKI reporting phenotype included acute kidney injury and renal failure acute. Normalization converted text to uppercase, collapsed punctuation and whitespace, and matched the complete expression. Historical expressions were retained as explicit aliases, not counted as additional events. Specificity refers only to the selected lexical pattern: interstitial nephritis need not be immune-mediated, proteinuria need not be VEGF-induced, and none was biopsy-adjudicated. **Supplementary Tables S2**, **S18** provide rationale and query details.

A report matching exactly one term family received that exclusive label; a report matching at least two received a mixed label. Broad/Tier-B concepts did not relabel reports. The renal-limited TMA anchor required the complete normalized expression RENAL LIMITED THROMBOTIC MICROANGIOPATHY; generic TMA or hemolytic-uremic syndrome did not qualify by inference. In JADER, immune-mediated renal disorder was not equated with immune-mediated nephritis and entered only an expanded-semantic sensitivity. In figures and compact tables, Immune-IS denotes the immune-interstitial reporting phenotype, VEGF-axis the VEGF-axis reporting phenotype, and AKI the nonspecific AKI reporting phenotype.

### 2.4 Active-comparator pharmacovigilance estimand

For each regimen–phenotype cell, we compared the odds of phenotype reporting with those for contemporaneous nivolumab plus ipilimumab reports. The denominator included all otherwise eligible strict-RCC reports. We calculated crude reporting odds ratios (RORs) and 95% confidence intervals, followed by report-year-stratified Mantel–Haenszel RORs (MH-RORs). When an annual stratum contained a zero cell, we added 0.5 to all four cells in that stratum. Confidence intervals used the Robins–Breslow–Greenland variance. The primary family contained nine prespecified regimen-by-phenotype cells. We adjusted two-sided Wald P-values with the Benjamini–Hochberg procedure. RORs were interpreted as relative reporting enrichment, not as incidence ratios, relative risks, or causal effects.

Prespecified FAERS sensitivity analyses restricted reports to healthcare professionals, allowed regimen overlap, or used sunitinib as the comparator. Further analyses omitted each report year or one Tier-A preferred-term family in turn. We also excluded reports with Japanese reporter or occurrence-country linkage. Sender concentration was quantified with the Herfindahl–Hirschman index, and regimen-specific top-sender exclusions were attempted. A top-sender result was evaluable only when both arms retained events and the stratified estimate remained identifiable.

### 2.5 Supervised machine-learning task and temporal validation

The secondary classifier was a methodological consistency check, informed by TRIPOD+AI and PROBAST+AI reporting principles (25, 26). Among reports already assigned an exclusive Tier-A label, it assessed how well non-anchor report features recovered that rule-derived label. The deterministic dictionary already assigns labels when anchors are present. The model therefore does not resolve an unknown clinical diagnosis, predict future injury, or validate pathology.

The development period was 2019Q1–2023Q4; reports from 2024Q1–2025Q4 were excluded from fitting, preprocessing estimation, and hyperparameter tuning. This temporal set was used for performance evaluation and the prespecified comparison of model families. The 2026Q1–Q2 audit used unchanged models and was descriptive if any class contained fewer than 20 reports. Exclusive reports lacking a usable initial FDA receipt date were included in full-history summaries but excluded from temporal modeling. Mixed reports were excluded from training, tuning, feature selection, and threshold selection.

M1 used multinomial elastic-net logistic regression with 55 predictors: age and missingness, sex, reporter status, country group, serious outcomes, and 36 non-anchor co-reported terms meeting a development prevalence threshold of 20. Label anchors, explicit aliases, and audited leakage families were excluded. This prevented direct anchor leakage but did not eliminate reporting-process or near-outcome information. Calendar time defined splits; time-to-onset and its missingness indicator were sensitivity-only. M2 added five binary, multi-hot indicators for the three target combinations, nivolumab plus ipilimumab, and the prespecified sunitinib reference definition. Unlike the primary pharmacovigilance analysis, the ML set retained other strict-RCC reports and overlapping indicator patterns. An all-zero vector is a heterogeneous remaining-report baseline, not nivolumab plus ipilimumab. Contrasts with nivolumab plus ipilimumab explicitly subtract the corresponding class coefficient. **Supplementary Tables S4**, **S8**, **S21**, **S22** document the analytic matrix and complete feature audit.

Elastic-net tuning considered six prespecified combinations of regularization strength and mixing parameter. Development used five-fold outer and three-fold inner stratified nested cross-validation. Final hyperparameters were selected using development data only, with class-balanced weights and no synthetic oversampling. XGBoost and LightGBM were constrained challengers using the M2 feature set. Under the prespecified rule, a challenger could replace M2 and trigger SHAP analysis only if its temporal macro-AUPRC exceeded M2. Thus, the temporal set also informed model-family retention and was not an independent test of a model selected without reference to those results. Neither challenger exceeded M2, which remained the prespecified reference classifier within the secondary analysis.

The primary endpoint, termed macro-AUPRC throughout, was the unweighted mean of three one-versus-rest average-precision (AP) scores (27), not trapezoidal integration of a macro-averaged precision–recall curve. Secondary endpoints included macro- and class-specific AUROC, class AP, balanced accuracy, macro-F1, sensitivity, precision, confusion matrices, multiclass Brier score, log loss, and one-versus-rest calibration. The M2–M1 difference used 500 paired, class-stratified temporal-validation bootstrap resamples and percentile 95% confidence intervals.

### 2.6 Mixed-case challenge and PV–ML triangulation

After model locking, we scored all 80 mixed reports without assigning a single correct class. Prespecified summaries included maximum predicted probability, normalized predictive entropy, top-two probability gap, and the three class probabilities. Lower maximum probability, higher entropy, and a smaller probability gap than that of exclusive reports were considered supportive of unresolved or dual reporting patterns.

For nine regimen-by-phenotype cells, we paired the log MH-ROR with the M2 regimen class-score contrast, summarizing sign agreement and descriptive Spearman correlation. An MH-ROR of 0.80–1.25 was prespecified as near null. This was an internal consistency check across different denominators: all eligible regimen reports for MH-ROR versus existing exclusive Tier-A reports for M2. Both reused FAERS and the same dictionary. The nine cells also shared comparator data and class structure; the correlation and its nominal P-value are descriptive, not independent replication evidence.

### 2.7 Cross-database directional corroboration in JADER

JADER processing applied strict RCC linkage, exclusive regimens, prespecified reporting phenotypes, active comparison, common support, year stratification, continuity correction, and multiplicity rules. Quantitative interpretation required at least three events in each arm and at least two jointly represented annual strata; other cells remained sparse directional observations. Sensitivities allowed concomitant roles, regimen overlap, broader immune semantics, and channel restrictions. We neither pooled databases nor treated JADER as external clinical validation. Japanese reporting pathways may feed both systems, and cross-system record linkage was unavailable.

### 2.8 Statistical analysis and reproducibility

Continuous variables are summarized by medians and interquartile ranges and categorical variables by counts and percentages. Descriptive characteristics were not significance-tested. Protocol v1.0 included covariate-adjusted Firth/Jeffreys models subject to event thresholds. These models were omitted because the covariate-complete reconstruction did not reproduce the primary cells, leaving year-stratified MH-ROR as the primary adjusted estimate. **Figure 1** robustness ranges used a separate reconstruction with matched target-event numerators. Small denominator differences produced a maximum absolute log-ROR difference of 0.0124; the original primary estimates remained the plotted baselines. *Section 2.9* distinguishes reanalysis of the original annual cells from ancillary report-level analyses. Analysis and figure-reproduction code, frozen query and feature manifests, annual contingency tables, fitted model parameters, and supporting documentation are publicly available in Zenodo (version 1.0.0; https://doi.org/10.5281/zenodo.22807359).

**Figure 1:**
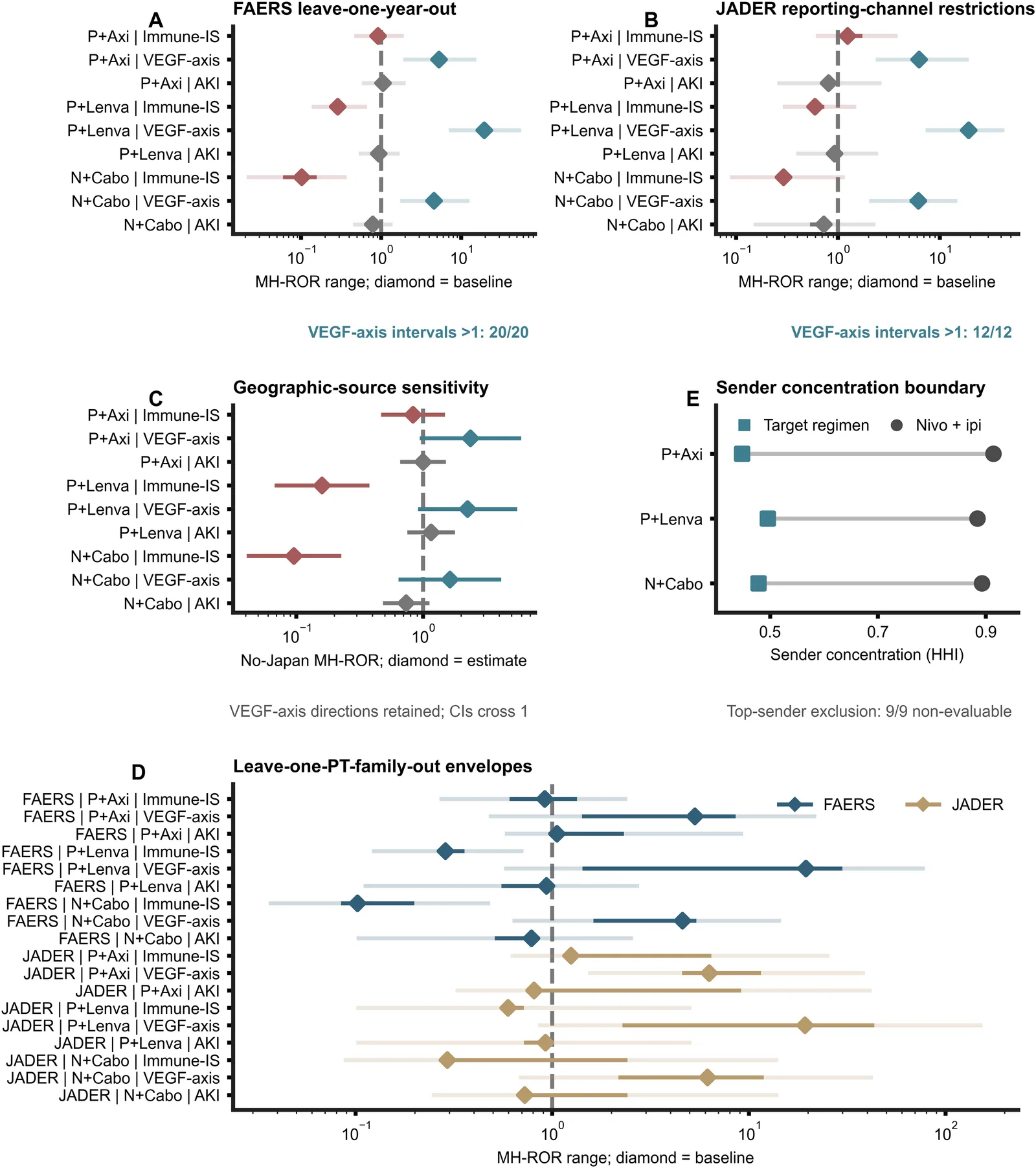
Reporting-source and term-family sensitivities of the VEGF-axis proxy. **(A)** Compact baseline points, point-estimate ranges, and full confidence-interval envelopes after leaving out one FAERS year. **(B)** Corresponding ranges across prespecified reporting channels in the updated JADER 202607 release through 2026Q1. All 20 FAERS leave-one-year and all 12 JADER channel-restricted VEGF-axis confidence intervals remain above one. **(C)** All nine FAERS phenotype estimates after excluding reports with Japanese reporter or occurrence country; all VEGF-axis directions are preserved, but all three confidence intervals cross one. **(D)** FAERS and JADER ranges after leaving out one Tier-A PT family. **(E)** Paired sender HHI for each target regimen and its contemporaneous nivolumab plus ipilimumab comparator. In **(A)** and the FAERS rows of **(D)**, diamonds are the primary estimates from **Table 2**, whereas ranges and envelopes come from the separately reconstructed robustness cohort. Target-event numerators matched the primary cells; small denominator differences produced a maximum |Δlog ROR| of 0.0124 and changed no direction. These analyses assess reporting architecture robustness and limitations, not incidence or causality. Immune-IS, immune-interstitial reporting phenotype; VEGF-axis, VEGF-axis reporting phenotype; AKI, nonspecific acute kidney injury reporting phenotype.

### 2.9 *Post–hoc* sensitivity analyses

*Post-hoc* analyses retained the original phenotype definitions, model specifications, and temporal splits. All nine primary estimates were reproduced from the original annual 2 × 2 cells before applying alternative sparse-cell handling. Alternatives included all-cell 0.1, zero-cell-only 0.5, uncorrected MH, and zero-stratum exclusion. We report raw cells, zero-stratum counts, MH denominator weights, and exploratory corrected-stratum heterogeneity; sparse strata limit heterogeneity assessment. The same annual cells supported exclusion of 2026.

Component, hypertension, hematuria, and nonrenal immune-related co-reporting analyses used the separately reconstructed strict-RCC report set. All 15,155 reports were joined to reaction records after retrieving 371 initially unmatched reports from their original latest-source quarterly files. This reconstruction is not identical to the frozen primary cohort; both denominators and differences are reported. Narrow SMQ retrieval used MedDRA 29.0 active PT-level entries with scope 2 for acute renal failure, proteinuria, and tubulointerstitial diseases. Exact current PT matching does not guarantee complete retrospective remapping of obsolete source terminology. Queries were evaluated separately without forcing mutual exclusivity. Component and co-report results are exploratory; zero-event arms are identified, and continuity-corrected finite values are not treated as biological evidence.

A regimen-only classifier and feature-ablated models used the locked elastic-net settings (C = 0.03, l1_ratio = 0, class-balanced loss), development-only imputation and age scaling, and unchanged temporal evaluation. Reporting-context ablation removed country indicators, HCP/profession-missing flags, and five seriousness/outcome variables. Renal-proximal ablation removed blood creatinine increased, hyperkalemia, and metabolic acidosis. These descriptive analyses did not retune on validation. Official trial-label safety summaries were tabulated with their original endpoint and grading definitions; no cross-trial incidence pooling was attempted.

## 3 Results

### 3.1 Cohort construction and temporal phenotype landscape

After global latest-version and deletion handling, the prespecified target-program candidate screen retained 574,338 unique FAERS CASEIDs. This count denotes the analysis-candidate subset rather than the complete FAERS universe. Of these, 84,369 met the strict RCC treatment-context definition. The Tier-A dictionary identified 2,307 reports: 219 exclusive immune-interstitial, 1,076 exclusive VEGF-axis, 932 exclusive nonspecific AKI, and 80 mixed reports. Mixed reports were separated before supervised modeling (**Figure 2**; **Supplementary Tables S1**, **S2**).

**Figure 2:**
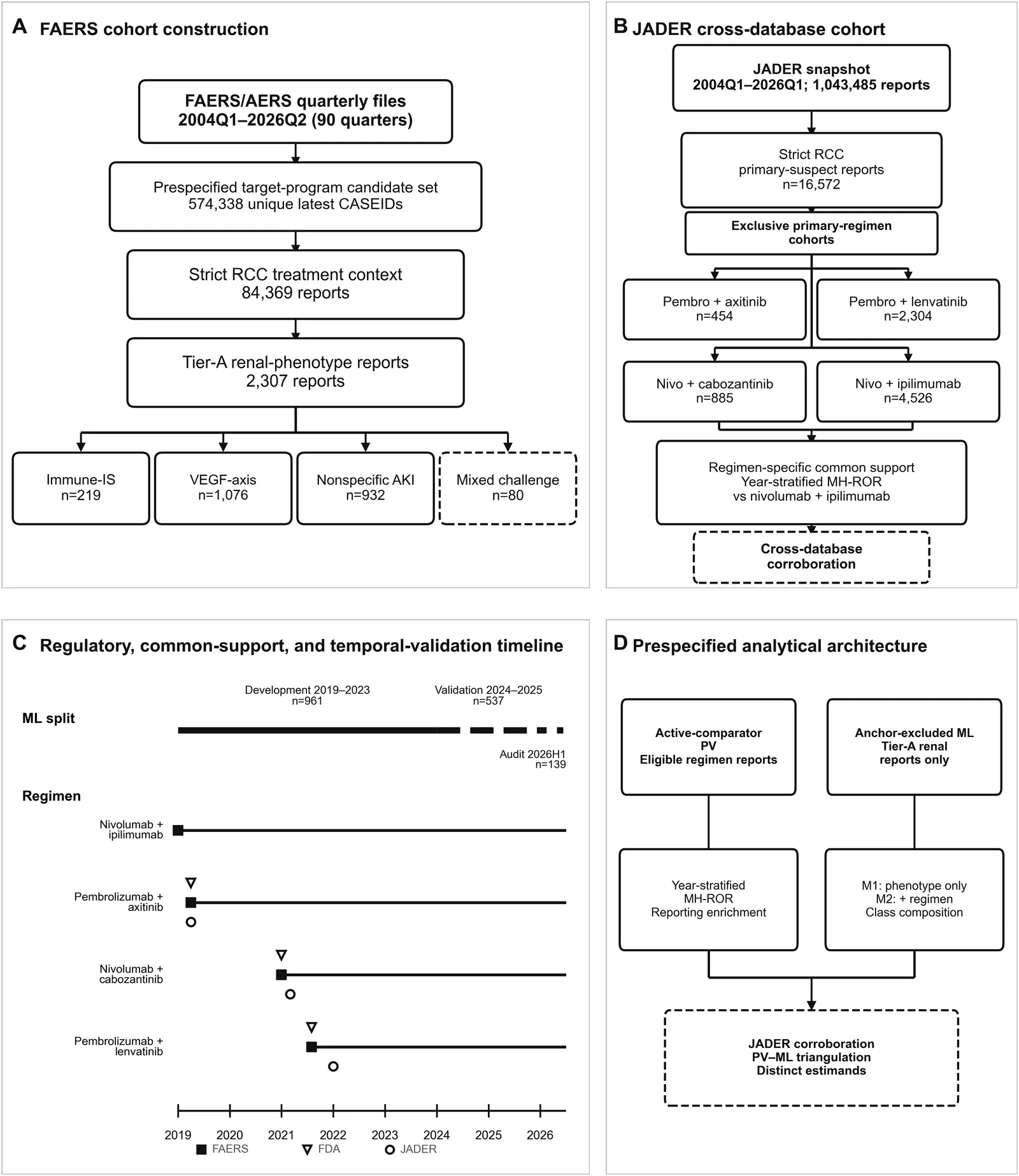
Cohort construction and prespecified analytical framework. **(A)** FAERS/AERS reports from 2004Q1 through 2026Q2 underwent global latest-version and deletion handling before construction of a prespecified target-program candidate set containing 574,338 unique latest CASEIDs; this count is not the complete FAERS database total. Within the strict renal cell carcinoma treatment context, reports meeting the author-defined Tier-A reporting definitions were assigned to mutually exclusive immune-interstitial, VEGF-axis, or nonspecific acute kidney injury phenotypes; mixed cases were reserved as a separate challenge set. **(B)** The updated JADER 202607 release covered 2004Q1 through 2026Q1 and was reconstructed using primary-suspect drug reports under the same strict RCC and exclusive-regimen principles, followed by regimen-specific common-support comparisons with nivolumab plus ipilimumab. The terminal 2026Q1 label contained only 37 released reports and was therefore treated as potentially truncated. **(C)** Regimen-specific common-support periods were aligned with RCC regulatory availability, while machine-learning development, temporal validation, and the locked 2026 descriptive audit were prespecified chronologically. **(D)** Active-comparator pharmacovigilance evaluated phenotype reporting enrichment among all eligible regimen reports, whereas M1 and M2 evaluated phenotype composition conditional on a Tier-A phenotype report. JADER supplies cross-database directional corroboration; PV–ML comparisons assess internal consistency across denominators. Neither establishes independent clinical replication. AKI, acute kidney injury; FAERS, FDA Adverse Event Reporting System; JADER, Japanese Adverse Drug Event Report database; MH-ROR, Mantel–Haenszel reporting odds ratio; ML, machine learning; PV, pharmacovigilance; RCC, renal cell carcinoma; VEGF, vascular endothelial growth factor. Immune-IS, immune-interstitial reporting phenotype; VEGF-axis, VEGF-axis reporting phenotype; AKI, nonspecific acute kidney injury reporting phenotype.

The full-history exclusive cohorts contained 2,446 pembrolizumab plus axitinib, 5,656 pembrolizumab plus lenvatinib, 5,649 nivolumab plus cabozantinib, and 4,566 nivolumab plus ipilimumab reports. Median age ranged from 66 to 68 years. Women comprised 25.0%–27.4% of reports with known sex. Healthcare-professional reporting ranged from 65.7% for nivolumab plus cabozantinib to 92.1% for nivolumab plus ipilimumab. Serious-outcome co-reporting ranged from 33.6% to 68.1%. These values describe reporting composition and were not compared inferentially (**Table 1**; **Supplementary Table S3**).

**Table 1:** FAERS report characteristics in the strict renal cell carcinoma regimen cohorts.

Characteristic	Pembrolizumab + axitinib	Pembrolizumab + lenvatinib	Nivolumab + cabozantinib	Nivolumab + ipilimumab
Reports, n	2,446	5,656	5,649	4,566
Report-year range	2015–2026	2016–2026	2016–2026	2014–2026
Age available, n (%)	2,042 (83.5)	4,160 (73.6)	4,378 (77.5)	3,590 (78.6)
Age, median (IQR), years	67 (60–74)	68 (60–74)	68 (60–75)	66 (58–72)
Sex available, n (%)	2,316 (94.7)	5,361 (94.8)	5,139 (91.0)	4,026 (88.2)
Female among known sex, n (%)	635 (27.4)	1,342 (25.0)	1,404 (27.3)	1,043 (25.9)
Reporter status available, n (%)	2,389 (97.7)	5,439 (96.2)	5,363 (94.9)	4,510 (98.8)
HCP among known reporter status, n (%)	1,874 (78.4)	4,775 (87.8)	3,526 (65.7)	4,154 (92.1)
Any serious outcome, n (%)	1,248 (51.0)	3,733 (66.0)	1,899 (33.6)	3,110 (68.1)
Hospitalization, n (%)	914 (37.4)	3,233 (57.2)	1,528 (27.0)	2,403 (52.6)
Death, n (%)	389 (15.9)	654 (11.6)	488 (8.6)	983 (21.5)
Reported drugs, median (IQR)	3 (2–6)	3 (2–7)	3 (2–9)	2 (2–5)

Values are n (%) unless otherwise indicated. Percentages for availability and outcomes use all regimen reports; female and HCP percentages use reports with known sex and reporter status, respectively.

These descriptive cohorts span 2004Q1–2026Q2 and are not the regimen-specific common-support denominators used for MH-ROR estimation. No between-regimen hypothesis tests were performed.

FAERS, FDA Adverse Event Reporting System; HCP, healthcare professional; IQR, interquartile range; RCC, renal cell carcinoma.

The 2,227 exclusive Tier-A reports were the full-history pool, not the number trained. Development contained 961 reports (136 immune-interstitial, 437 VEGF-axis, 388 AKI); temporal validation contained 537 (51/355/131); and the descriptive 2026 audit contained 139 (14/79/46). A further 426 predated 2019, and 164 lacked usable initial-report dates (3/57/104). Thus 1,637 exclusive reports entered the three chronological windows. Of development reports, 450 had none of the five regimen indicators, 493 had exactly one, and 18 had multiple; the corresponding validation counts were 207, 322, and 8. **Supplementary Table S4** reports counts by phenotype, date availability, analysis period, and regimen-indicator pattern. All 80 mixed reports remained outside training and tuning. The temporal and reporting-phenotype landscape of the cohort is summarized in **Figure 3**.

**Figure 3:**
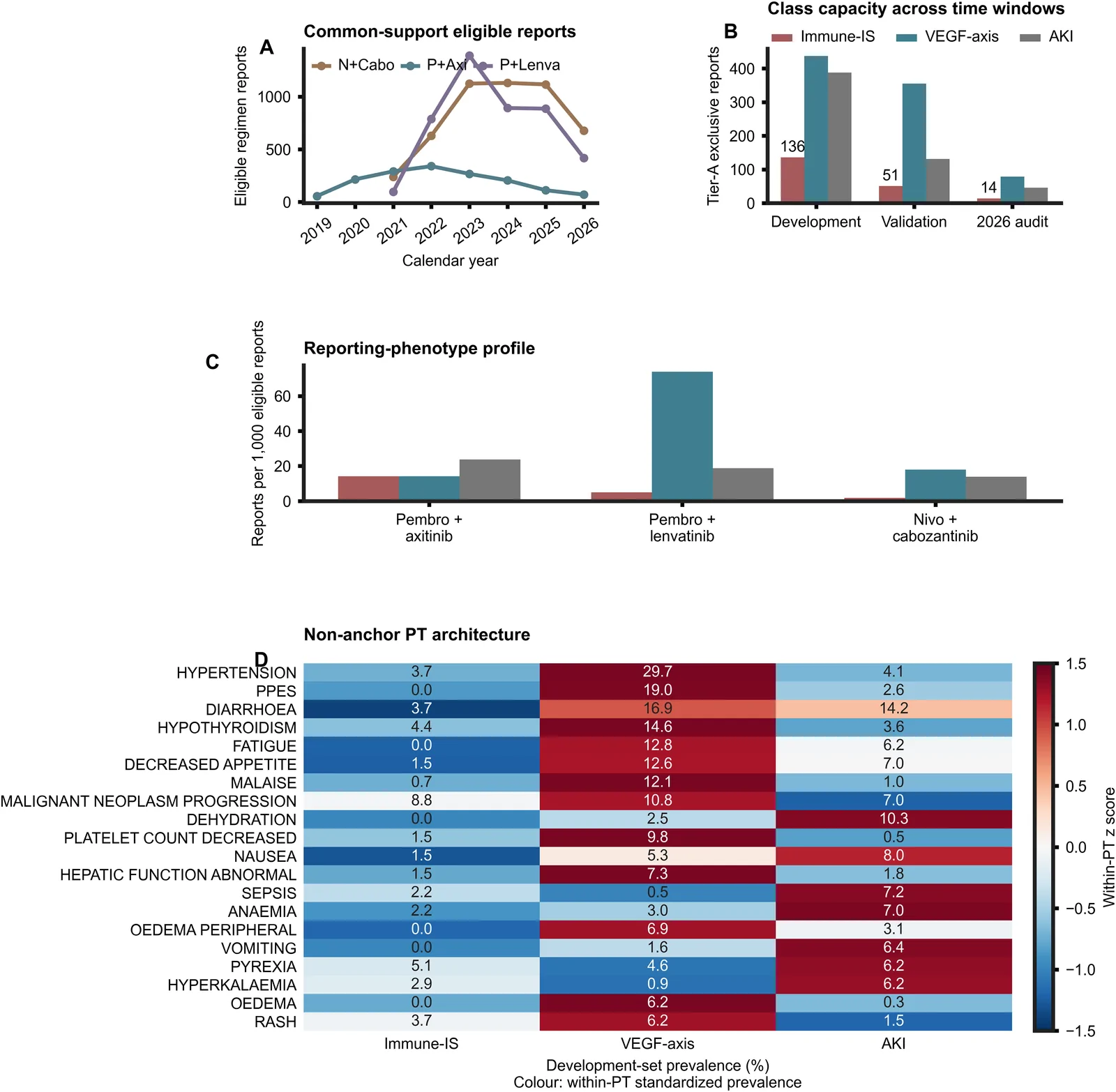
Temporal and reporting-phenotype landscape of the RCC renal-injury cohort. **(A)** Annual eligible FAERS reports for each ICI–VEGFR-TKI regimen within its regimen-specific common-support period with nivolumab plus ipilimumab. **(B)** Tier-A exclusive class counts in the prespecified development, temporal-validation, and 2026 locked-audit windows; the 14 immune-interstitial reports in 2026 retain that window as descriptive only. **(C)** Cumulative proxy-phenotype reporting proportions per 1,000 eligible regimen reports over each regimen-specific common-support window, not annual rates or treated-patient incidence. **(D)** The 20 retained non-anchor preferred terms with the highest development-set class prevalence. Cell labels are absolute prevalence percentages and color is standardized within each PT to emphasize class architecture. Immune-IS, immune-interstitial reporting phenotype; VEGF-axis, VEGF-axis reporting phenotype; AKI, nonspecific acute kidney injury reporting phenotype.

### 3.2 Active-comparator analysis identifies selective VEGF-axis enrichment

The primary analysis produced a phenotype-specific 3 × 3 pattern (**Figure 4**; **Table 2**). Relative to nivolumab plus ipilimumab, pembrolizumab plus axitinib had a VEGF-axis MH-ROR of 5.31 (95% CI 2.43–11.59; BH q = 6.18 × 10^-5^). Its immune-interstitial estimate was near null at 0.92 (0.55–1.52), and its nonspecific AKI estimate was 1.05 (0.71–1.56). Pembrolizumab plus lenvatinib had the largest VEGF-axis estimate at 19.48 (9.03–42.01; q = 3.32 × 10^-13^). Its immune-interstitial estimate was 0.29 (0.16–0.50; q = 3.09 × 10^-5^), and its AKI estimate was 0.93 (0.63–1.39). Nivolumab plus cabozantinib also showed VEGF-axis enrichment at 4.59 (2.07–10.19; q = 3.13 × 10^-4^). Corresponding estimates were 0.10 (0.05–0.22; q = 3.27 × 10^-8^) for immune-interstitial reporting and 0.78 (0.53–1.17) for AKI.

**Figure 4:**
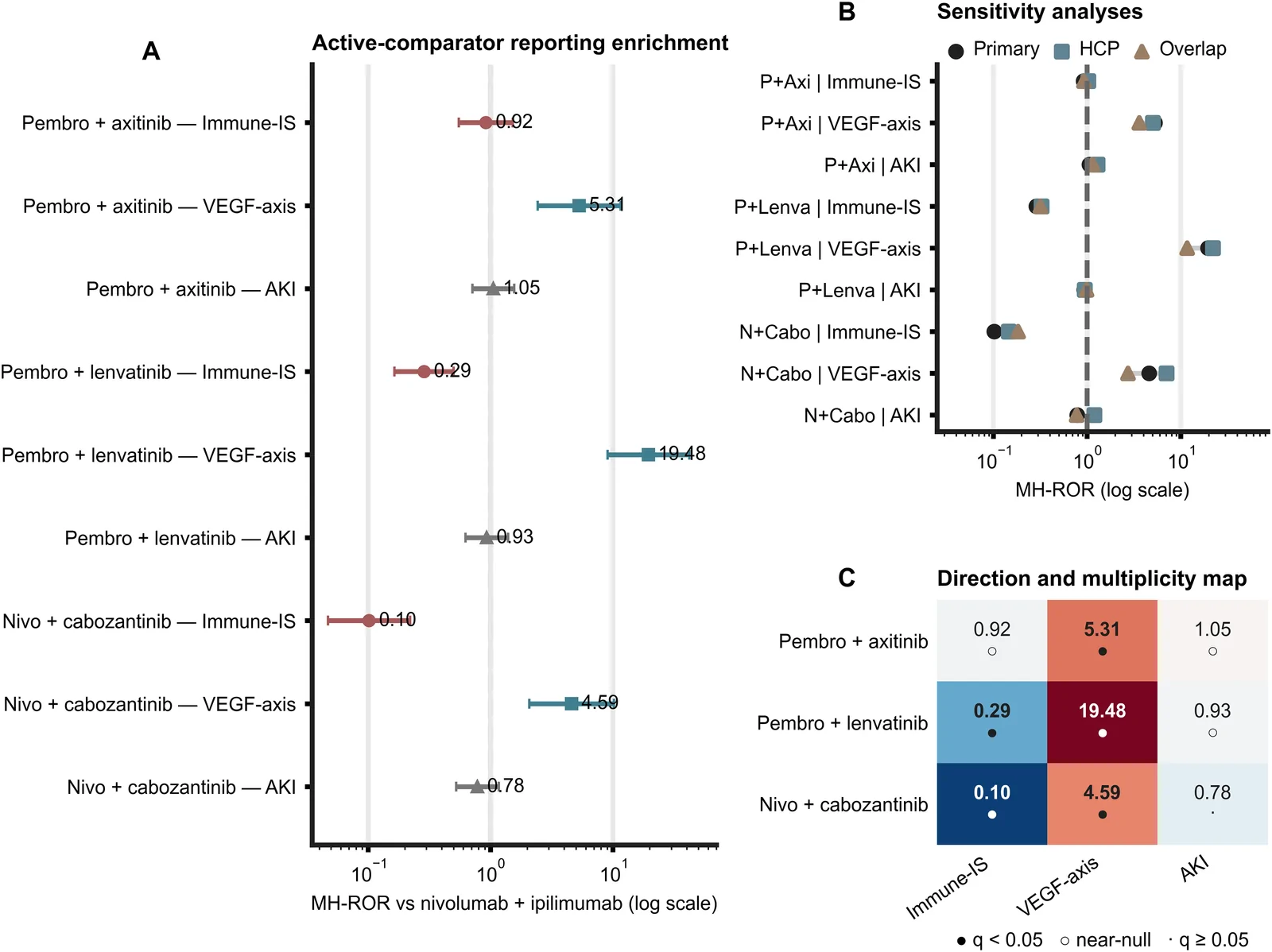
Active-comparator pharmacovigilance identifies selective VEGF-axis reporting enrichment. **(A)** Year-stratified Mantel–Haenszel reporting odds ratios (MH-RORs) compare each ICI–VEGFR-TKI regimen with nivolumab plus ipilimumab from the regimen-specific RCC common-support start; points and bars denote MH-RORs and 95% confidence intervals. **(B)** Primary estimates are compared with HCP-only and regimen-overlap-allowed sensitivity analyses. **(C)** The prespecified 3 × 3 direction matrix displays MH-RORs on a log2 color scale and distinguishes multiplicity-controlled signals, near-null cells, and non-significant directions. Reporting enrichment is not an incidence rate or causal effect. Immune-IS, immune-interstitial reporting phenotype; VEGF-axis, VEGF-axis reporting phenotype; AKI, nonspecific acute kidney injury reporting phenotype.

**Table 2:** Primary FAERS active-comparator analysis versus nivolumab plus ipilimumab.

Target regimen	Tier-A phenotype	Common-support start	Target events/reports	Comparator events/reports	Crude ROR (95% CI)	Year-stratified MH-ROR (95% CI)	P	BH q	PV–M2 classification
Pembrolizumab + axitinib	Immune-IS	2019Q2	22/1,556	50/3,779	1.07 (0.65–1.77)	0.92 (0.55–1.52)	0.735	0.790	directional concordance; near-null
Pembrolizumab + axitinib	VEGF-axis	2019Q2	22/1,556	7/3,779	7.73 (3.29–18.13)	5.31 (2.43–11.59)	2.75e-05	6.18e-05	strong concordance
Pembrolizumab + axitinib	Nonspecific AKI	2019Q2	37/1,556	91/3,779	0.99 (0.67–1.45)	1.05 (0.71–1.56)	0.790	0.790	near-null estimand divergence
Pembrolizumab + lenvatinib	Immune-IS	2021Q3	22/4,471	34/1,992	0.28 (0.17–0.49)	0.29 (0.16–0.50)	1.03e-05	3.09e-05	strong concordance
Pembrolizumab + lenvatinib	VEGF-axis	2021Q3	330/4,471	6/1,992	26.38 (11.74–59.25)	19.48 (9.03–42.01)	3.69e-14	3.32e-13	strong concordance
Pembrolizumab + lenvatinib	Nonspecific AKI	2021Q3	84/4,471	46/1,992	0.81 (0.56–1.17)	0.93 (0.63–1.39)	0.737	0.790	directional concordance; near-null
Nivolumab + cabozantinib	Immune-IS	2021Q1	9/4,914	37/2,299	0.11 (0.05–0.23)	0.10 (0.05–0.22)	7.26e-09	3.27e-08	strong concordance
Nivolumab + cabozantinib	VEGF-axis	2021Q1	88/4,914	6/2,299	6.97 (3.04–15.96)	4.59 (2.07–10.19)	1.74e-04	3.13e-04	strong concordance
Nivolumab + cabozantinib	Nonspecific AKI	2021Q1	68/4,914	54/2,299	0.58 (0.41–0.84)	0.78 (0.53–1.17)	0.233	0.349	directional concordance; near-null

The comparator was nivolumab plus ipilimumab. Common-support periods were regimen-specific and aligned with RCC regulatory availability.

P-values are two-sided Wald tests for the year-stratified Mantel–Haenszel estimate; q values use Benjamini–Hochberg correction across the nine prespecified cells.

PV–M2 agreement was classified by sign, effect magnitude, and multiplicity-adjusted evidence. Strong concordance required same-signed log(MH-ROR) and M2 class-score contrasts, BH q <0.05, and |log(MH-ROR)| ≥ log(1.25). The label “directional concordance; near-null” groups other same-sign cells; not every estimate in this category lies within the near-null interval. Near-null estimand divergence denotes opposite signs with |log(MH-ROR)| < log(1.25); substantive estimand divergence denotes opposite signs outside that band. The prespecified open near-null interval was 0.80–1.25.

RORs quantify reporting enrichment and must not be interpreted as incidence ratios or causal effects. AKI, acute kidney injury; BH, Benjamini–Hochberg; CI, confidence interval; MH-ROR, Mantel–Haenszel reporting odds ratio; PV, pharmacovigilance; RCC, renal cell carcinoma; VEGF, vascular endothelial growth factor; Immune-IS, immune-interstitial reporting phenotype.

All three multiplicity-adjusted positive primary cells were VEGF-axis. None of the exclusive Tier-A AKI cells was enriched, a finding limited to that exclusive definition and not all acute-renal-failure queries. HCP-only and overlap-allowed analyses retained the principal pattern (**Figure 4B**; **Supplementary Tables S5**, **S6**). Sunitinib comparisons were heterogeneous (**Supplementary Table S7**; **Supplementary Figures S4**-**S6**), reinforcing their secondary role without calibrating differences in surveillance.

### 3.3 Regimen information adds modest classification performance in temporal evaluation

The 2024–2025 temporal-validation set contained 537 exclusive reports. M1 achieved a macro-AUPRC of 0.606 and a macro-AUROC of 0.822. Its balanced accuracy was 0.559, macro-F1 was 0.554, and multiclass Brier score was 0.417. M2 increased macro-AUPRC to 0.638 and macro-AUROC to 0.840. Balanced accuracy increased to 0.609, macro-F1 to 0.601, and the Brier score decreased to 0.383 (**Figure 5**; **Table 3**).

**Figure 5:**
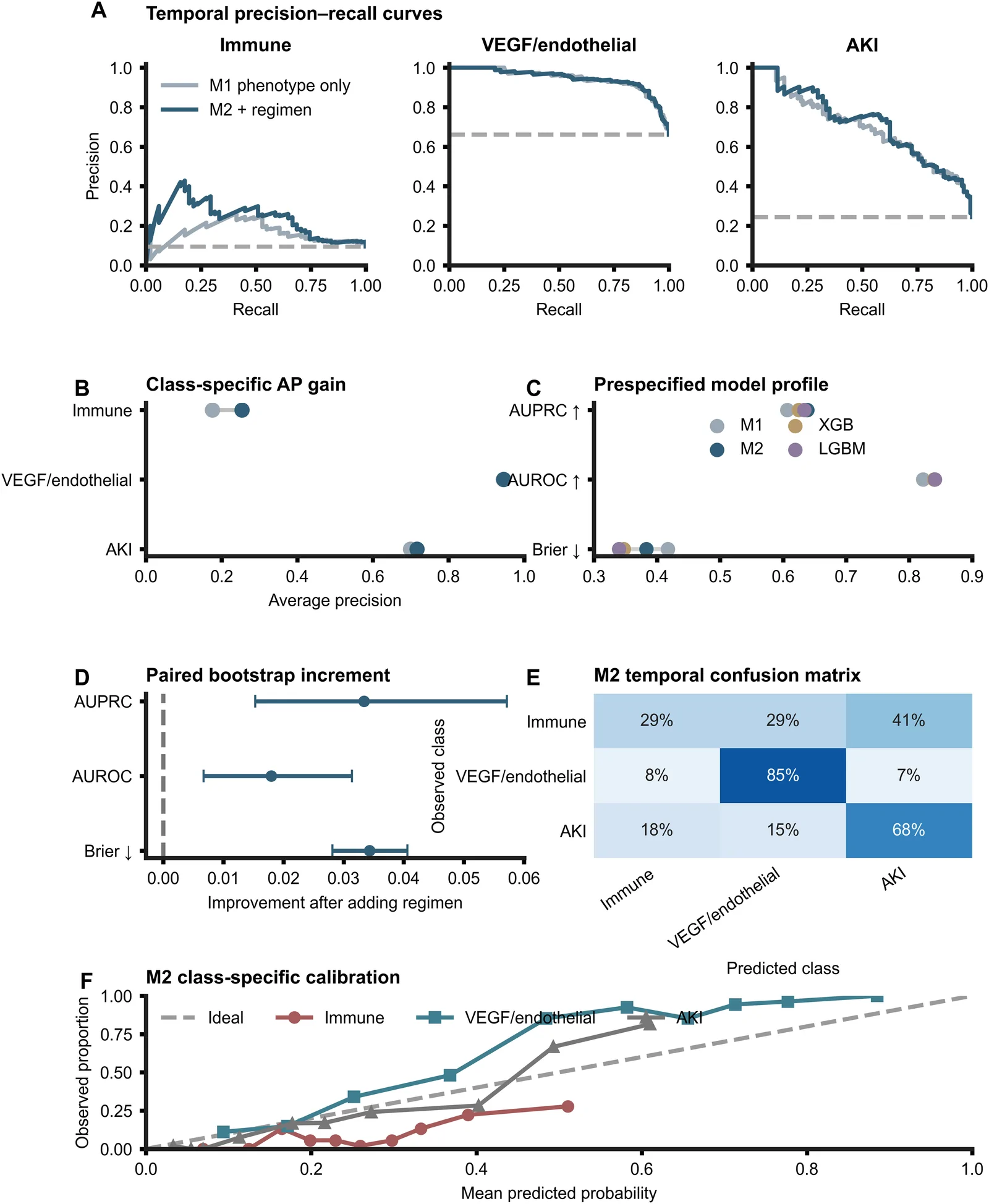
Regimen information improves prespecified temporal classification, with the immune-interstitial proxy remaining the hardest class. **(A)** One-versus-rest precision–recall curves for the anchor-excluded M1 phenotype-only model and M2 after addition of five regimen indicators in the 2024–2025 validation set (n = 537); dashed lines denote class prevalence. **(B)** Class-specific average precision. **(C)** Prespecified performance profile across the reference and nonlinear challenger models. **(D)** Means and percentile 95% intervals of 500 paired bootstrap M2-minus-M1 differences (the observed sample differences are reported in **Table 3** and **Supplementary Table S10**); the Brier difference is sign-inverted only for display so that rightward values consistently denote improvement, while raw signed differences are retained in source data. **(E)** Row-normalized M2 confusion matrix. **(F)** Quantile-binned class-specific M2 calibration in the same temporal validation set; the immune curve displays the most important probability-reliability limitation. Model-family comparisons also used this temporal set; fitting and hyperparameter tuning used development data only. Immune-IS, immune-interstitial reporting phenotype; VEGF-axis, VEGF-axis reporting phenotype; AKI, nonspecific acute kidney injury reporting phenotype.

**Table 3:** Prespecified machine-learning performance in the 2024–2025 temporal validation set.

Model or contrast	n	Macro-AUPRC	Macro-AUROC	Balanced accuracy	Macro-F1	Multiclass Brier	Immune-IS AP	VEGF-axis AP	AKI AP	Role/decision
A. Temporal-validation performance										
M1 multinomial elastic-net	537	0.606	0.822	0.559	0.554	0.417	0.175	0.944	0.699	Baseline M1
M2 multinomial elastic-net + regimen	537	0.638	0.840	0.609	0.601	0.383	0.253	0.946	0.717	Reference M2
XGBoost challenger	537	0.625	0.840	0.612	0.607	0.347	0.219	0.943	0.713	Challenger
LightGBM challenger	537	0.634	0.841	0.598	0.595	0.339	0.237	0.938	0.726	Challenger
B. Prespecified M2 versus M1 paired bootstrap contrasts										
M2 minus M1 (observed point difference; 95% bootstrap CI)	500 resamples	+0.0321 (+0.0153 to +0.0572)	+0.0179 (+0.0067 to +0.0314)	–	–	−0.0342 (−0.0406 to −0.0282)	–	–	–	Regimen increment retained

Macro-AUPRC is the mean of three one-versus-rest AP scores, not trapezoidal integration. M2 was the prespecified reference classifier in the secondary consistency analysis. The temporal set was excluded from fitting and hyperparameter tuning but informed comparison with the nonlinear challengers.

The M2–M1 macro-AUPRC point difference is the observed sample difference (+0.0321); +0.0334 is the mean of the 500 paired bootstrap differences and is not substituted for the observed point estimate.

Lower Brier scores indicate better probability accuracy. AP, average precision; AKI, acute kidney injury; AUROC, area under the receiver-operating-characteristic curve; CI, confidence interval; VEGF, vascular endothelial growth factor; Immune-IS, immune-interstitial reporting phenotype.

The observed M2–M1 macro-AUPRC difference was 0.0321 (paired bootstrap 95% CI 0.0153–0.0572). The macro-AUROC difference was 0.0179 (0.0067–0.0314), and the Brier score difference was −0.0342 (−0.0406 to −0.0282). With M2, average precision was 0.253 for the immune-interstitial reporting phenotype, 0.946 for the VEGF-axis reporting phenotype, and 0.717 for nonspecific AKI. Recall was 0.294, 0.854, and 0.679, respectively. The immune-interstitial class was therefore the principal performance limitation (**Supplementary Table S9**; **Supplementary Figure S7**).

Neither nonlinear challenger exceeded M2 on temporal macro-AUPRC; detailed results are provided in **Supplementary Tables S9**, **S10** and **Supplementary Figures S7**, **S8**. The 2026 M2 audit yielded macro-AUPRC 0.719 and macro-AUROC 0.857, but remained descriptive because the immune-interstitial class contained only 14 reports. *Post-hoc* models achieved macro-AUPRC 0.453 with regimen indicators alone, 0.586 after removing reporting-context variables, and 0.583 after additionally removing three renal-proximal variables. The latter were blood creatinine increased, hyperkalemia, and metabolic acidosis. Removing blood creatinine increased alone yielded 0.639. After reporting-context removal, immune-interstitial AP increased from 0.253 to 0.363, whereas VEGF-axis AP decreased from 0.946 to 0.883 and AKI AP from 0.717 to 0.511. These descriptive class-specific differences were not tested with paired difference intervals, and the ablations did not select or replace M2 (**Supplementary Table S21**).

### 3.4 Model coefficients and uncertainty in mixed reports

The signed M2 regimen coefficients reproduced the qualitative active-comparator pattern. All three ICI–VEGFR-TKI indicators favored the VEGF-axis class relative to the nivolumab plus ipilimumab reporting pattern. The largest negative immune-interstitial contrasts occurred for pembrolizumab plus lenvatinib and nivolumab plus cabozantinib. These coefficients describe attribution within M2 and do not represent biological or causal effects (**Figures 6A, B**; **Supplementary Data Tables SD1**, **SD2**).

**Figure 6:**
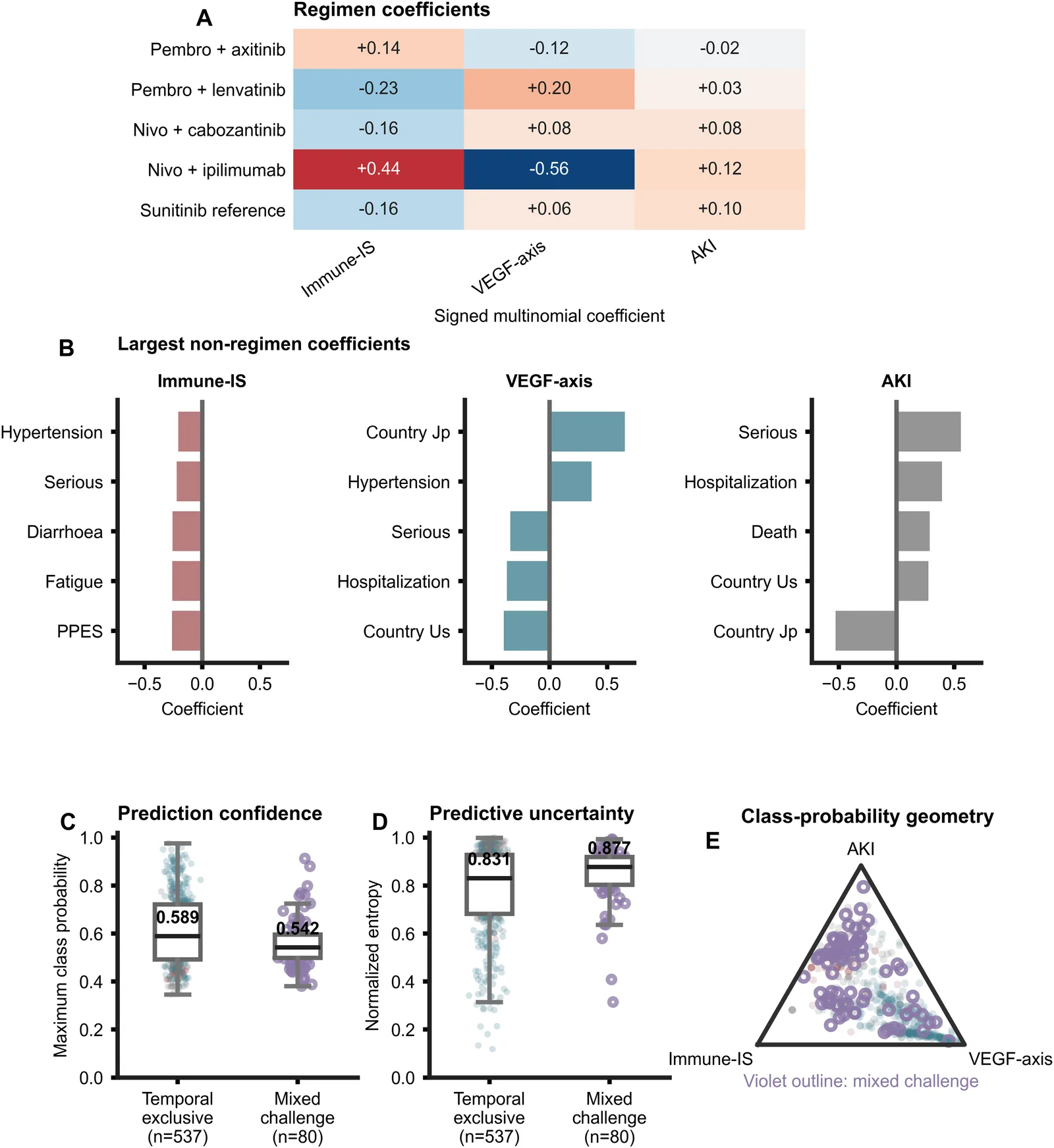
Locked model coefficients and uncertainty in mixed reports. **(A)** Regimen coefficients across the three proxy-label classes. **(B)** Five largest absolute non-regimen coefficients per class after the anchor-family audit. Coefficients are predictive associations, not biological effects. **(C, D)** Maximum probability and normalized entropy in temporal-exclusive reports (n = 537) and all 80 mixed reports, which were excluded from training and tuning. Boxes show medians and interquartile ranges; jitter is visual only. **(E)** Class probabilities on a simplex. Distributional overlap and the absence of clinical adjudication preclude interpreting intermediate probabilities as proven dual pathology. Immune-IS, immune-interstitial reporting phenotype; VEGF-axis, VEGF-axis reporting phenotype; AKI, nonspecific acute kidney injury reporting phenotype.

Compared with exclusive temporal-validation reports, the 80 mixed reports had lower M2 confidence. Median maximum predicted probability was 0.542 versus 0.589, and normalized entropy was 0.877 versus 0.831. The median top-two probability gap was 0.239 versus 0.322. In the simplex projection, mixed reports occupied broader intermediate regions rather than one class (**Figures 6C–E**; **Supplementary Table S11**; **Supplementary Figure S9**). This pattern was consistent with a challenge to forced single-label classification. It did not establish that mixed reports represented clinically adjudicated dual pathology.

### 3.5 Internal agreement between pharmacovigilance and classification

Active-comparator log MH-RORs and M2 regimen class-score contrasts had the same direction in eight of nine prespecified cells (**Figure 7**). No cell showed substantive discordance. The sole sign difference involved pembrolizumab plus axitinib and nonspecific AKI. Its MH-ROR was 1.05, within the prespecified near-null band. The descriptive Spearman correlation between log MH-ROR and the M2 contrast was 0.85 (P = 0.0037).

**Figure 7:**
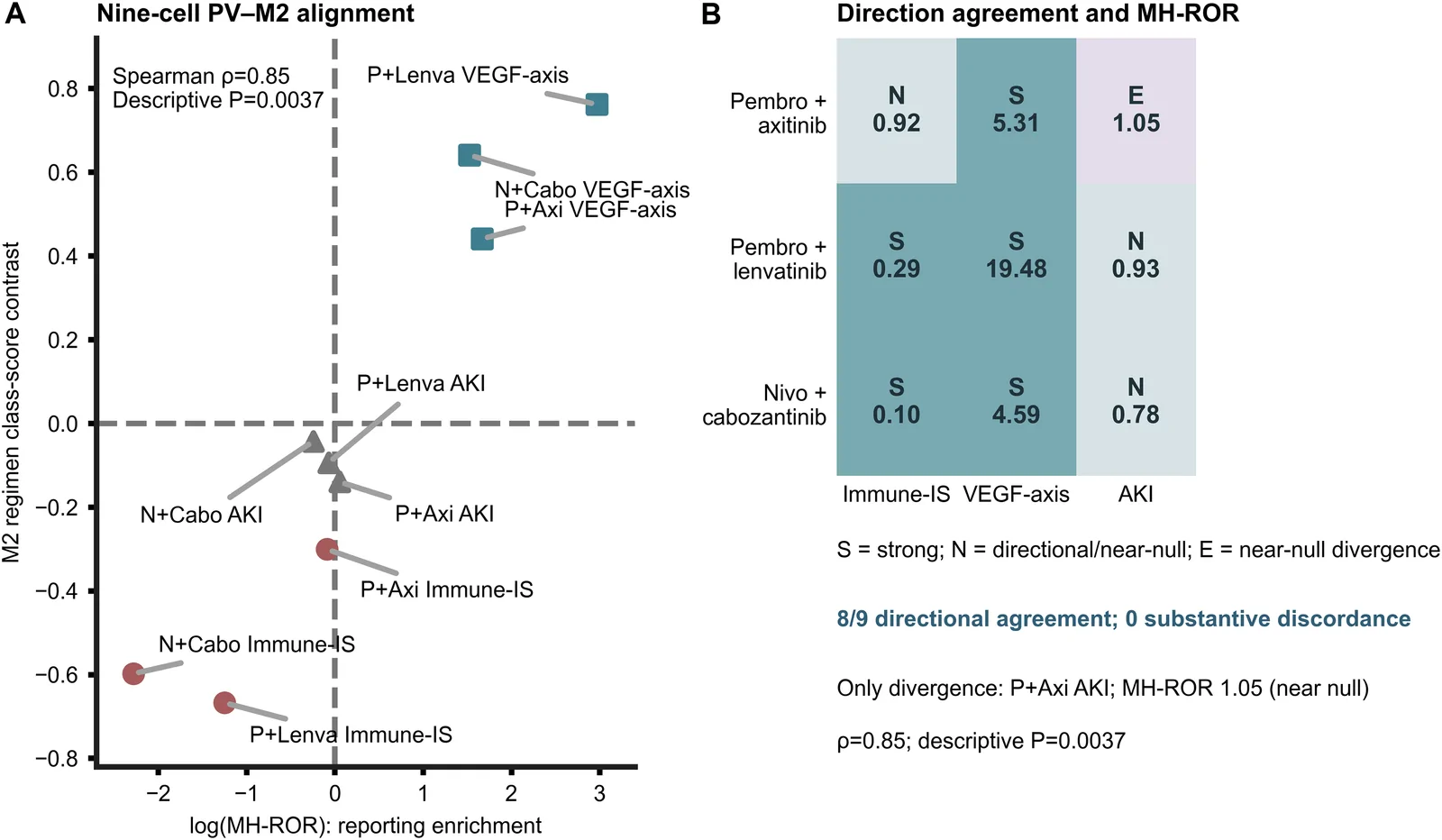
Internal agreement across two reporting estimands. **(A)** Log MH-ROR versus locked M2 regimen class-score contrast for nine prespecified cells. Spearman correlation and nominal P-value are descriptive because cells share data, comparator reports and class structure. **(B)** Eight of nine cells agree in sign; the exception is the near-null pembrolizumab–axitinib AKI cell (MH-ROR 1.05). As shown in **Figure 2D**, the denominators differ: all eligible regimen reports for pharmacovigilance and exclusive Tier-A renal reports for classification. Agreement does not independently validate the labels or their biological mechanism. Immune-IS, immune-interstitial reporting phenotype; VEGF-axis, VEGF-axis reporting phenotype; AKI, nonspecific acute kidney injury reporting phenotype.

Eight-of-nine sign agreement is an internal consistency result, not statistically independent corroboration. Both analyses reuse FAERS and the same reporting dictionary, despite their different denominators. Shared comparators and correlated class scores further limit inferential interpretation of a correlation across nine cells.

### 3.6 JADER provides cross-database directional corroboration

The JADER 202607 release contained 1,043,485 reports, including 16,572 strict-RCC primary-suspect reports. Before common-support restriction, regimen-exclusive cohorts included 454 pembrolizumab plus axitinib, 2,304 pembrolizumab plus lenvatinib, 885 nivolumab plus cabozantinib, and 4,526 nivolumab plus ipilimumab reports. Seven of nine cells met the quantitative evaluability criteria. Pembrolizumab plus axitinib AKI and nivolumab plus cabozantinib immune-interstitial reporting remained sparse directional cells. **Supplementary Figure S1** and **Supplementary Tables S1**, **S12** describe cohort construction. **Supplementary Data Tables SD6**, **SD7** provide quarterly support and the annual 2 × 2 strata.

The three multiplicity-adjusted positive JADER cells were all VEGF-axis (**Figure 8**; **Table 4**). The pembrolizumab plus axitinib MH-ROR was 6.28 (95% CI 2.46–16.02; q = 3.68 × 10^-4^). For pembrolizumab plus lenvatinib, it was 19.27 (8.59–43.21; q = 6.21 × 10^-12^). The estimate for nivolumab plus cabozantinib was 6.16 (2.55–14.91; q = 2.47 × 10^-4^). Quantitatively evaluable immune-interstitial estimates were 1.25 (0.61–2.53) and 0.60 (0.30–1.19) for the two pembrolizumab regimens. The nivolumab plus cabozantinib immune-interstitial cell contained one target event and remained directional only (0.29, 0.09–0.99). JADER AKI estimates were 0.81, 0.92, and 0.73, with wide intervals and no multiplicity-adjusted enrichment.

**Figure 8:**
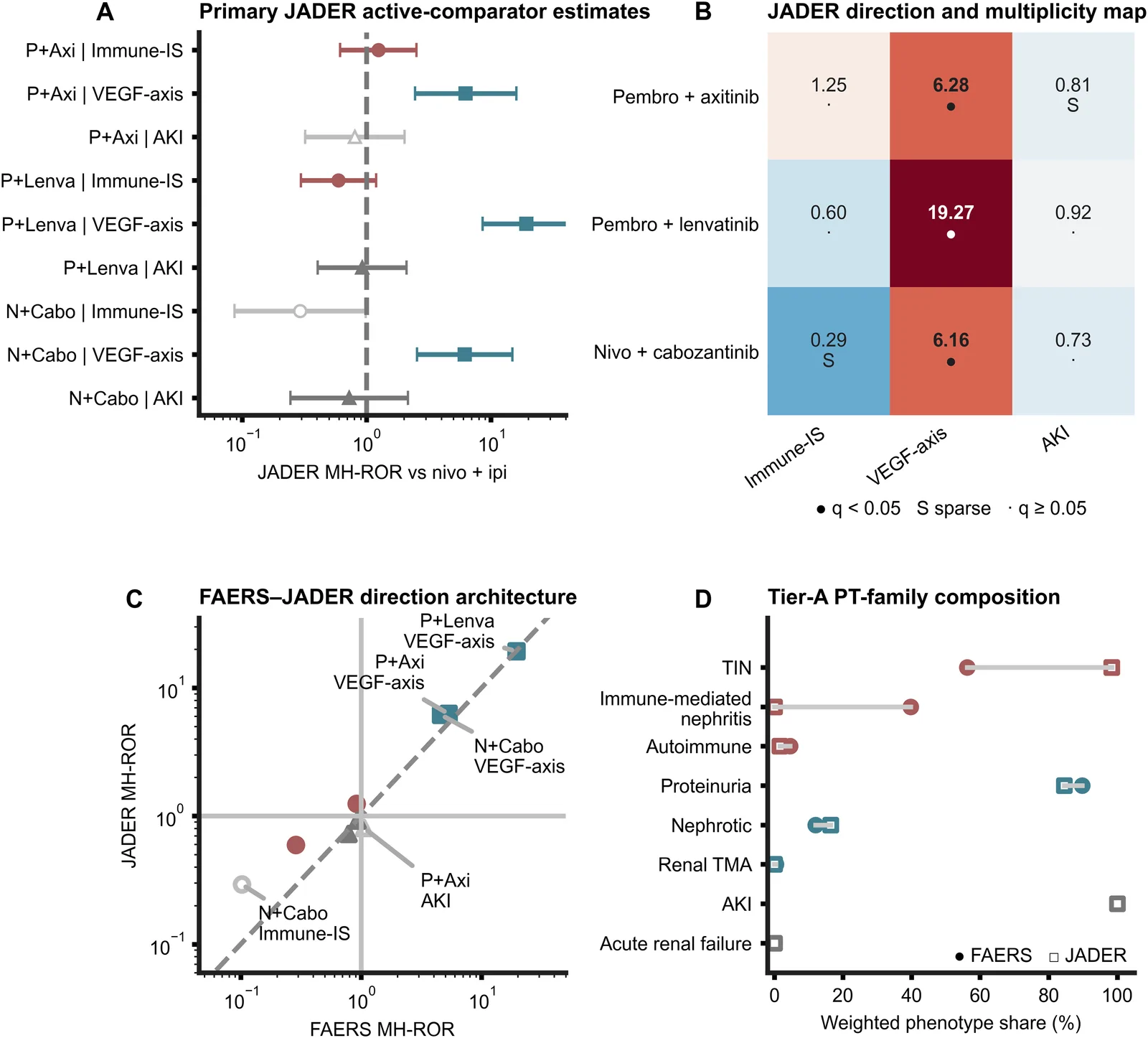
Cross-database JADER analysis supports the VEGF-axis reporting pattern. **(A)** Year-stratified JADER MH-RORs for primary-suspect, exclusive RCC regimens; open gray symbols denote sparse directional cells. The 202607 release covers reports through 2026Q1; the terminal quarter contains 37 reports and may be truncated. **(B)** The 3 × 3 JADER direction matrix; all three multiplicity-controlled positive cells are VEGF-axis. **(C)** FAERS and JADER MH-RORs for the same nine cells, including seven quantitatively evaluable cells and two sparse directional cells. The diagonal indicates equality, not a replication criterion. **(D)** Weighted Tier-A PT-family shares; families can co-occur and are not a mutually exclusive partition. JADER provides directional corroboration, but shared reporting pathways and report overlap could not be assessed without cross-system record linkage. Immune-IS, immune-interstitial reporting phenotype; VEGF-axis, VEGF-axis reporting phenotype; AKI, nonspecific acute kidney injury reporting phenotype.

**Table 4:** Cross-database comparison of FAERS and JADER active-comparator estimates.

Target regimen	Tier-A phenotype	FAERS events, target/comparator	FAERS MH-ROR (95% CI)	FAERS BH q	JADER events, target/comparator	JADER MH-ROR (95% CI)	JADER BH q	JADER evaluability	Cross-database classification
Pembrolizumab + axitinib	Immune-IS	22/50	0.92 (0.55–1.52)	0.790	7/54	1.25 (0.61–2.53)	0.724	quantitatively evaluable	FAERS near null not directional
Pembrolizumab + axitinib	VEGF-axis	22/7	5.31 (2.43–11.59)	6.18e-05	5/6	6.28 (2.46–16.02)	3.68e-04	quantitatively evaluable	directionally concordant
Pembrolizumab + axitinib	Nonspecific AKI	37/91	1.05 (0.71–1.56)	0.790	2/50	0.81 (0.32–2.03)	0.730	sparse directional	inconclusive due to sparsity
Pembrolizumab + lenvatinib	Immune-IS	22/34	0.29 (0.16–0.50)	3.09e-05	14/21	0.60 (0.30–1.19)	0.260	quantitatively evaluable	directionally concordant
Pembrolizumab + lenvatinib	VEGF-axis	330/6	19.48 (9.03–42.01)	3.32e-13	184/6	19.27 (8.59–43.21)	6.21e-12	quantitatively evaluable	directionally concordant
Pembrolizumab + lenvatinib	Nonspecific AKI	84/46	0.93 (0.63–1.39)	0.790	12/12	0.92 (0.41–2.10)	0.849	quantitatively evaluable	FAERS near null not directional
Nivolumab + cabozantinib	Immune-IS	9/37	0.10 (0.05–0.22)	3.27e-08	1/27	0.29 (0.09–0.99)	0.107	sparse directional	inconclusive due to sparsity
Nivolumab + cabozantinib	VEGF-axis	88/6	4.59 (2.07–10.19)	3.13e-04	19/6	6.16 (2.55–14.91)	2.47e-04	quantitatively evaluable	directionally concordant
Nivolumab + cabozantinib	Nonspecific AKI	68/54	0.78 (0.53–1.17)	0.349	3/18	0.73 (0.24–2.15)	0.724	quantitatively evaluable	directionally concordant

FAERS covered 2004Q1–2026Q2. JADER used the 202607 release covering 2004Q1–2026Q1; the terminal quarter contained 37 released reports and may be truncated.

A JADER cell was quantitatively evaluable only when target and comparator each contributed at least three phenotype events and at least two annual strata were available; all other cells were classified as sparse directional, retained only for direction, and not promoted to quantitative corroboration.

Cross-database agreement was classified hierarchically: inconclusive due to sparsity for non-evaluable JADER cells; FAERS near null not directional when the FAERS MH-ROR lay strictly between 0.80 and 1.25; directionally concordant when the remaining signs agreed; JADER near null or imprecise when signs differed but the JADER estimate was near null or its 95% CI included 1; and directionally discordant otherwise.

All three JADER multiplicity-controlled positive cells were VEGF-axis.

The databases use different national reporting systems. Directional agreement does not establish independent reports; cross-system record linkage was unavailable. BH, Benjamini–Hochberg; CI, confidence interval; FAERS, FDA Adverse Event Reporting System; JADER, Japanese Adverse Drug Event Report database; MH-ROR, Mantel–Haenszel reporting odds ratio; VEGF, vascular endothelial growth factor; Immune-IS, immune-interstitial reporting phenotype.

All five informative non-null cells were directionally concordant. Across seven quantitatively evaluable cells, descriptive Spearman correlation was 0.964 (nominal P = 0.0005). Concomitant-role, overlap, semantic, and channel sensitivities retained the three VEGF-axis directions (**Supplementary Tables S13**, **S14**; **Supplementary Figure S10**). Report-level overlap and shared Japanese reporting pathways could not be excluded.

### 3.7 Robustness analyses preserve direction and define signal limits

All 20 FAERS leave-one-year-out VEGF-axis intervals remained above one. The same was true for all 12 JADER reporting-channel-restricted intervals (**Figures 1A, B**; **Supplementary Figure S11**; **Supplementary Table S15**; **Supplementary Data Table SD5**). After excluding FAERS reports linked to Japan, all three point estimates remained above one. Their confidence intervals crossed one, indicating a substantial loss of precision (**Figure 1C**).

Proteinuria accounted for 75.0%–95.8% of target VEGF-axis events in FAERS and 60.0%–92.9% in JADER. Removing this family preserved six point-estimate directions, but only one JADER interval excluded one. The robust empirical content is therefore a proteinuria-dominant reporting pattern; positive directions among the remaining sparse terms do not establish an independent glomerular or endothelial injury signal (**Figure 1D**; **Supplementary Data Tables SD3**, **SD4**).

Comparator reports were more sender-concentrated. All nine top-sender exclusions became non-evaluable after event collapse or loss of identifiability, so no finite post-exclusion estimate was interpreted (**Figure 1E**). Geographic restriction also retained positive VEGF-axis point estimates but lost interval exclusion of one. Together these findings leave reporting-source explanations unresolved rather than demonstrating source-independent robustness.

### 3.8 Sensitivity to phenotype definitions and sparse strata

All nine primary estimates were reproduced to numerical precision. Alternative sparse-cell handling retained the positive VEGF-axis direction but did not make the magnitudes suitable for safety ranking (**Supplementary Table S16**). Excluding 2026 gave VEGF-axis MH-RORs of 6.45 (95% CI 2.77–15.02), 21.36 (9.28–49.20), and 5.05 (2.13–12.00) for pembrolizumab plus axitinib, pembrolizumab plus lenvatinib, and nivolumab plus cabozantinib, respectively. JADER primary estimates were unchanged after removing 2026 strata (**Supplementary Table S17**).

In the separate reconstructed report set, the narrow proteinuria query remained enriched for all three combinations. However, the narrow acute-renal-failure query yielded 1.09 (0.83–1.42), 1.56 (1.20–2.02), and 0.73 (0.55–0.97), respectively. Thus the original absence of exclusive Tier-A AKI enrichment did not generalize across renal definitions. Component estimates concentrated on proteinuria; nephrotic-syndrome intervals crossed one, and renal-limited TMA was too sparse for quantitative interpretation. Hypertension-related reports were enriched, whereas hematuria intervals crossed one; these observations do not distinguish surveillance from injury mechanisms (**Supplementary Table S18**).

Among exclusive AKI reports in the reconstructed set, explicit nonrenal immune/autoimmune organ terms occurred in 0/37, 6/91, 6/70, and 21/94 reports for pembrolizumab plus axitinib, pembrolizumab plus lenvatinib, nivolumab plus cabozantinib and nivolumab plus ipilimumab. A broader irAE-compatible query yielded 2/37, 22/91, 15/70, and 39/94. These are co-reporting proportions, not reclassified immune-nephritis cases; neither absence nor presence establishes renal etiology (**Supplementary Table S19**).

## 4 Discussion

This study identifies a proteinuria-dominant VEGF-axis reporting pattern in three RCC ICI–VEGFR-TKI combinations relative to nivolumab plus ipilimumab. The direction recurred in JADER and persisted when 2026 strata were excluded. It does not establish endothelial pathology or a quantitative ordering of renal safety. The original AKI result is also narrower than a general absence of renal risk: exclusive Tier-A AKI was not enriched, whereas the separate narrow acute-renal-failure query was enriched for pembrolizumab plus lenvatinib. The AKI interpretation therefore depends on the renal phenotype definition.

Recent Frontiers in Immunology studies examined organ-specific reports with ICI combinations and VEGF/VEGFR inhibitors (28–30). Our contribution is a more constrained reporting comparison using indication-linked RCC cohorts, regimen-specific common support, transparent term families, and a second national system. These design choices reduce some heterogeneity but cannot turn reporting contrasts into adjudicated etiologies.

Real-world RCC comparisons show that combination regimens serve overlapping but non-identical populations (31, 32). FAERS cannot match treatment line, IMDC risk, clear-cell versus non-clear-cell histology, perioperative use, prior treatment, or baseline renal function. Uptake after CLEAR may also change the volume, geography, and clinical composition of pembrolizumab plus lenvatinib reports. Annual volumes help expose this process but cannot adjust it away. The PD-1–CTLA-4 comparator is clinically relevant, not an exchangeable control or a way to isolate TKI addition.

Differential ascertainment is the principal competing explanation. VEGFR-TKI product information calls for proteinuria and blood-pressure surveillance, whereas monitoring intensity and thresholds may differ under dual ICI therapy. More frequent urine testing can increase reported proteinuria even without greater underlying injury. Neither database records the number of tests, their timing, dose intensity, or systematic negative results. The available sensitivities cannot rule out surveillance accounting for some or all of the enrichment. Sunitinib also prompts renal monitoring, but its era, pharmacology, population, and reporting history differ; its heterogeneous results do not calibrate surveillance intensity.

Trial-monitored incidence answers a different question. In official KEYNOTE-426 safety summaries, grade 3–4 creatinine increase was 4.3% with pembrolizumab plus axitinib versus 2.4% with sunitinib. CLEAR reported grade 3–4 grouped AKI at 5% versus 2%, and proteinuria at 8% versus 3%. Corresponding grade 3–4 creatinine abnormalities were 1.3% versus 0.6% in CheckMate 9ER and 2.1% versus 1.7% in CheckMate 214. **Supplementary Table S20** presents the different endpoints, safety populations, and label sources; serious AKI is not equated with grade ≥3 AKI. The trials (4–7) were not head-to-head comparisons between these combinations. Nonetheless, their clinically important renal events show why a non-enriched spontaneous-report AKI proportion cannot imply a low patient-level incidence.

Immune undercapture is a central internal-validity threat. ICI-associated interstitial nephritis may be reported only as AKI, placing a clinically immune event in the nonspecific class. AKI after ICI exposure has several competing causes and substantial clinical consequences (33, 34). Biomarker approaches, including urine proteomics and CXCL9, are being investigated but do not supply a universally validated reference standard (35–37). Biopsy remains important when attribution is uncertain (38). Nonrenal immune co-reports within the AKI group are compatible with undercapture, but cannot identify which renal cases are immune-mediated; their absence is equally uninformative.

The comparator’s own reporting background adds asymmetry. Dual ICI immune toxicity is well recognized, and post-CheckMate 214 awareness or labeling may preferentially elicit specific immune-nephritis terminology. A lower target immune ROR may therefore reflect numerator composition or notoriety rather than less immune renal injury. Clinical management should remain grounded in established irAE guidance rather than this classifier or a below-one reporting ratio (39, 40).

VEGF inhibition provides a plausible biological context for proteinuria and hypertension (41–43), but plausibility is not validation. Proteinuria supplied most target events; removing it left sparse, imprecise component estimates. Nephrotic syndrome has several etiologies, and the exact renal-limited TMA anchor was too rare for inference. Hypertension enrichment and imprecise hematuria contrasts add reporting context, not biopsy evidence. The term VEGF-axis is a pharmacologically motivated proxy label, not a diagnosis assigned to each report.

Only seven, six, and six comparator VEGF-axis events supported the primary comparisons, with zero cells in five of eight, two of six, and two of six annual strata. Alternative corrections and uncorrected MH estimates preserved the positive direction, but neither these sensitivities nor low-power heterogeneity statistics make a value such as 19.48 stable enough for ranking. Source review and top-sender event collapse further limit confidence in magnitude. We emphasize a directional proteinuria reporting pattern and its uncertainty.

The model addresses a secondary methodological question: whether non-anchor co-reports recover labels already determinable by rules. M2 improved macro-AUPRC by 0.0321, but recovered only 15 of 51 immune-interstitial validation reports. Regimen-only macro-AUPRC was 0.453; removing reporting-context and renal-proximal features yielded 0.583, compared with 0.638 for full M2. Reporting-context removal had different effects across classes: immune-interstitial AP increased from 0.253 to 0.363, while VEGF-axis and AKI AP decreased. The overall reduction therefore does not imply that reporting-context variables improved classification of every phenotype. These *post-hoc* comparisons describe class-dependent performance and do not establish that the remaining information is biological.

Chronological evaluation, anchor exclusion, and development-only preprocessing reduced direct leakage into fitting and hyperparameter tuning. However, the temporal set also informed model-family retention, limiting interpretation as a fully independent test of the selected model. The all-zero regimen group represents heterogeneous remaining reports, not the active comparator. Mixed reports showed greater uncertainty on average, with substantial distributional overlap. They also spanned different eras from the temporal-exclusive group, so class composition and reporting context could contribute to the uncertainty difference. Without clinical adjudication, these probabilities describe reporting-label consistency rather than diagnostic performance. Detailed challenger results are provided in the supplement.

Cross-database analyses can examine whether reporting directions recur under different national coding and processing systems (44–48). We reconstructed JADER rather than pooling its reports with FAERS. Its three VEGF-axis directions agreed with FAERS, while sparse and near-null cells were not counted as quantitative replications. This supports cross-database directional corroboration, with no claim of external clinical validation.

Report completeness and the reporting process vary across systems (49), and disproportionality can be distorted by stimulation, masking, selection, and duplication (50). Japan-linked FAERS exclusion retained positive point estimates but widened all three intervals to cross one. That finding is compatible with geographic influence and loss of information; it does not itself prove duplicate reports or quantify dependence. Without cross-system linkage, shared Japanese and manufacturer pathways remain unresolved. Channel-restricted and leave-one-year analyses also reuse reports and are not independent confirmations.

Other FAERS–JADER studies have described second-database agreement using different terminology and evaluability rules (51–53). The present findings support directional corroboration. The 37 terminal JADER reports and uncertain FAERS terminal maturity motivated exclusion of 2026. Persistence after exclusion reduces concern about those strata but does not eliminate release lag, retrospective updates, or stimulated reporting during earlier years.

The study used indication-linked cohorts, prespecified primary comparisons, explicit sparse-cell handling, and separate reporting of *post-hoc* analyses. Limitations include absent treated-person denominators, incomplete clinical covariates, unadjudicated labels, sparse immune-interstitial events, and concentrated reporting sources. The ancillary report-level reconstruction was not identical to the primary cohort; its counts and numerical differences are reported separately.

Further progress requires clinical anchoring. RCC cohorts with serial creatinine, quantitative proteinuria, urine sediment, blood pressure, concomitant medication, and treatment timing could distinguish monitoring effects from renal injury. Dechallenge, rechallenge, nephrology adjudication, and selected biopsy findings would be needed to test etiologic assignments. Contemporary onco-nephrology standards support such clinical-pathological evaluation (54, 55). Clinically adjudicated cohorts are needed to determine whether these reporting patterns identify actionable renal phenotypes.

## 5 Conclusion

The principal finding is a proteinuria-dominant VEGF-axis reporting pattern for three RCC ICI–VEGFR-TKI combinations relative to nivolumab plus ipilimumab, with cross-database directional corroboration in JADER. The absence of enrichment for exclusive Tier-A AKI is definition-specific and does not extend to every renal-failure query. Differential monitoring, sparse comparators, immune undercapture, and shared reporting ecology remain important alternative explanations. Secondary classification supports internal label consistency, not renal diagnosis, pathology, comparative incidence, or treatment selection.

## Data Availability

The original contributions presented in the study are included in the article/**Supplementary Material**. Further inquiries can be directed to the corresponding author.
