# Supplementary material for: Renal injury phenotypes with ICI–VEGFR-TKI combinations in renal cell carcinoma: active-comparator analyses in FAERS and JADER
Source: Front Immunol. 2026 Oct 14;17:1971413. doi: 10.3389/fimmu.2026.1971413 (PMC13713503; doi:10.3389/fimmu.2026.1971413)
Supplement: Supplementary file 1 [file Table1.docx]

**SUPPLEMENTARY MATERIAL**

**Contents**

| **Fig.** | **Supplementary figures** | **p.** | **Table** | **Supplementary tables** | **p.** |
| --- | --- | --- | --- | --- | --- |
| **S1** | JADER data and regimen audit | 2 | **S1** | Cohorts and support windows | 13 |
| **S2** | Label–predictor separation | 3 | **S2** | Definitions and language mapping | 14 |
| **S3** | Reporting context and missingness | 4 | **S3** | Report characteristics | 15 |
| **S4** | Annual-stratum diagnostics | 5 | **S4** | Exact ML cohort accounting | 16 |
| **S5** | FAERS sensitivity analyses | 6 | **S5** | Annual primary 2×2 cells | 17 |
| **S6** | Historical sunitinib comparator | 7 | **S6** | FAERS sensitivity estimates | 19 |
| **S7** | Temporal model diagnostics | 8 | **S7** | Historical sunitinib comparator | 20 |
| **S8** | Challengers and 2026 audit | 9 | **S8** | Feature and leakage audit | 21 |
| **S9** | Coefficients and mixed reports | 10 | **S9** | Complete temporal metrics | 25 |
| **S10** | JADER cohort sensitivities | 11 | **S10** | Model contrasts and 2026 audit | 27 |
| **S11** | Reporting-source robustness | 12 | **S11** | Mixed-report uncertainty | 28 |
|  |  | — | **S12** | JADER release audit | 29 |
|  |  | — | **S13** | JADER sensitivity estimates | 30 |
|  |  | — | **S14** | Cross-database concordance | 32 |
|  |  | — | **S15** | Source and year robustness | 33 |
|  |  |  | **S16** | Sparse-cell analysis | 35 |
|  |  |  | **S17** | Terminal-period exclusion | 38 |
|  |  |  | **S18** | Standard queries and components | 39 |
|  |  |  | **S19** | Reporting context and co-reports | 40 |
|  |  |  | **S20** | Phase III trial context | 42 |
|  |  |  | **S21** | Regimen-only and ablated models | 43 |
|  |  |  | **S22** | Full retained PT audit | 43 |
|  |  |  | **S23** | Reporting-standard maps | 45 |
|  |  |  | **S24** | 2026 reference verification | 47 |

**Supplementary Figure S1 | JADER data-quality, report-grain and regimen-exclusivity audit.** (A) Audited report funnel from the updated JADER 202607 release (2004Q1–2026Q1) to strict-RCC primary-suspect reports and the five exclusive regimen cohorts. The final value is summed cohort membership, not a new unique-report denominator. The terminal 2026Q1 label contains 37 released reports and may be truncated. (B) All-match and exclusive primary-suspect counts. (C) Regimen-specific overlap exclusions and burden; pairwise overlap identities were not exported and are not inferred. (D) PASS and informational checks by audit domain. Immune-IS, immune-interstitial reporting phenotype; VEGF-axis, VEGF-axis reporting phenotype; AKI, nonspecific acute kidney injury reporting phenotype.

Supplementary Figure S2 | Separation of Tier-A labels from anchor-excluded M1 predictors. (A) Candidate PT disposition after leakage and prevalence gates. (B) Prespecified anchor and semantic-family blacklist entries. (C) Prevalence of all 36 retained non-anchor PTs across development, temporal validation and the descriptive 2026 audit, stratified by class and split into two consecutive 18-PT display blocks. (D) Pairwise Jaccard similarity among the retained PTs. No Tier-A anchor, direct synonym or mapped leakage family appears in the retained predictor set. Immune-IS, immune-interstitial reporting phenotype; VEGF-axis, VEGF-axis reporting phenotype; AKI, nonspecific acute kidney injury reporting phenotype.

**Supplementary Figure S3 | Reporting characteristics, missingness and sensitivity-only time to onset.** (A) Availability of age and sex. (B) HCP source and serious-outcome composition among time-eligible exclusive Tier-A reports. (C) TTO missingness rises from 48.8% in development to 61.6% in temporal validation and 64.0% in the descriptive 2026 audit. (D) Median and interquartile range among reports with observed TTO. Because missingness is substantial and time-varying, TTO is not a primary predictor and is interpreted only in sensitivity analysis. Immune-IS, immune-interstitial reporting phenotype; VEGF-axis, VEGF-axis reporting phenotype; AKI, nonspecific acute kidney injury reporting phenotype.

**Supplementary Figure S4 | Annual-stratum diagnostics for the FAERS active-comparator analysis.** (A) Eligible target-regimen reports by year. (B) Annual Tier-A event counts summed over the three target regimens. (C) Crude RORs versus year-stratified Mantel–Haenszel RORs. (D) Within-cell normalized contribution of each year to the sum of Mantel–Haenszel numerator and denominator cross-product terms. This diagnostic shows that supported estimates are distributed across calendar strata rather than being assigned an inverse-variance interpretation. Immune-IS, immune-interstitial reporting phenotype; VEGF-axis, VEGF-axis reporting phenotype; AKI, nonspecific acute kidney injury reporting phenotype.

**Supplementary Figure S5 | Prespecified FAERS sensitivity analyses preserve the primary reporting architecture.** (A–C) Year-stratified MH-RORs for the primary exclusive cohort, HCP-only reports and the overlap-allowed specification, shown on identical logarithmic axes for immune, VEGF-axis and AKI phenotypes. (D) Direction relative to one for all 27 regimen-by-phenotype-by-specification estimates. These are sensitivity analyses of the same reporting system and are not independent samples. Immune-IS, immune-interstitial reporting phenotype; VEGF-axis, VEGF-axis reporting phenotype; AKI, nonspecific acute kidney injury reporting phenotype.

**Supplementary Figure S6 | Sunitinib provides a historical/mechanistic sensitivity comparator, not the primary modern active comparator.** (A) Nine year-stratified MH-RORs versus sunitinib. (B) Comparison with the corresponding nivolumab–ipilimumab estimates shows comparator-dependent direction changes, including five substantive estimand divergences. (C) Regimen and comparator report support within common-support periods. Because sunitinib crosses treatment eras and occupies a VEGF-mechanism position, these results are secondary sensitivity evidence. Immune-IS, immune-interstitial reporting phenotype; VEGF-axis, VEGF-axis reporting phenotype; AKI, nonspecific acute kidney injury reporting phenotype.

**Supplementary Figure S7 | Temporal discrimination and calibration of the prespecified classifiers.** (A,B) One-versus-rest precision–recall and receiver-operating-characteristic curves for all four models in the 2024–2025 temporal set (n=537). Dashed precision baselines denote class prevalence. (C) Class-specific one-versus-rest Brier scores. (D) Class-specific calibration intercepts and slopes. (E) Row-normalized confusion matrices. Quantile-binned M2 calibration curves are shown in Figure 4F. Fitting, preprocessing estimation, and hyperparameter tuning used development data only. The temporal set informed model-family comparison under the prespecified macro-AUPRC rule. Neither challenger exceeded M2; it remained the reference classifier in the secondary analysis, and SHAP analysis was not initiated. Immune-IS, immune-interstitial reporting phenotype; VEGF-axis, VEGF-axis reporting phenotype; AKI, nonspecific acute kidney injury reporting phenotype.

**Supplementary Figure S8 | Challenger models and the locked 2026 descriptive audit.** (A) Paired bootstrap contrasts show that XGBoost and LightGBM did not improve the locked macro-AUPRC endpoint over M2; negative differences denote improvement only for Brier score and log loss. (B,C) M2 aggregate and class-specific metrics in formal 2024–2025 temporal validation and the neutral-grey 2026 Q1–Q2 locked audit. (D) Case-level maximum probability and normalized entropy across the two periods. All 2026 results are descriptive only because the immune class contained 14 reports; they are not presented as a second validation set. Immune-IS, immune-interstitial reporting phenotype; VEGF-axis, VEGF-axis reporting phenotype; AKI, nonspecific acute kidney injury reporting phenotype.

**Supplementary Figure S9 | Model coefficients and uncertainty in mixed reports.** (A) The 15 regimen-by-class M2 coefficients represent predictive associations. (B) Mean M2 class probabilities by challenge time stratum; the 2026 stratum contains one report and is descriptive. (C) Maximum probability, normalized entropy, and top-two probability gap for four models in the 80 mixed reports excluded from training, tuning, and threshold selection. Complete signed coefficients are provided in SD1_M1_coefficients.csv and SD2_M2_coefficients.csv in the accompanying reproducibility archive. Immune-IS, immune-interstitial reporting phenotype; VEGF-axis, VEGF-axis reporting phenotype; AKI, nonspecific acute kidney injury reporting phenotype.

**Supplementary Figure S10 | Updated JADER cohort construction and prespecified sensitivity analyses.** The 202607 release covers 2004Q1–2026Q1; the terminal quarter contains 37 released reports and may be truncated. (A) All-match and exclusive primary-suspect regimen counts. (B) Primary-suspect-only estimates compared with the expanded suspect-or-concomitant role definition. (C) Results when regimen overlap is allowed. (D) Primary Tier-A phenotypes compared with expanded immune semantics. (E) Quarterly regimen report support. Sensitivity cohorts reuse reports from the same JADER source and are not independent samples; paired displays are used only to assess architectural stability under alternative construction rules. Immune-IS, immune-interstitial reporting phenotype; VEGF-axis, VEGF-axis reporting phenotype; AKI, nonspecific acute kidney injury reporting phenotype.

**Supplementary Figure S11 | Complete source-level robustness diagnostics underlying Figure 8.** (A) Cell-level FAERS MH-RORs after each eligible calendar year is omitted. (B) Cell-level MH-RORs from the updated JADER 202607 release through 2026Q1 under four reporting-channel definitions. In A and B colour denotes log2 MH-ROR and white cells denote an unsupported year–cell combination. (C) Change from the cell-specific baseline after leaving out one Tier-A PT family; families not belonging to a phenotype are blank. These ratios use the internal baseline from the separately reconstructed robustness cohort; diamonds in Figure 8A and 8D denote the primary FAERS estimates. Target-event numerators matched the primary cells, and max |Δlog ROR| was 0.0124. (D) JADER estimates compared with FAERS estimates after excluding reports linked to Japan. (E) Target and comparator report retention after regimen-specific top-sender exclusion. All nine exclusion cells are non-evaluable because positivity or zero-event requirements fail, so finite post-exclusion estimates are not interpreted; this is an explicit reporting-system limitation. Complete PT-family composition and leave-one-out records are provided in SD3_PT_family_composition.csv and SD4_Leave_one_PT_family.csv in the accompanying reproducibility archive. Immune-IS, immune-interstitial reporting phenotype; VEGF-axis, VEGF-axis reporting phenotype; AKI, nonspecific acute kidney injury reporting phenotype.

**Supplementary Table S1. Data sources, cohort counts, temporal analysis sets, and common-support starts**

| **Item/window/regimen** | **Value/total n** | **Definition** | **Database / Immune-IS n** | **VEGF n** | **AKI n** | **Mixed n** |
| --- | --- | --- | --- | --- | --- | --- |
| **A. Data-source and cohort funnel** | | | | | | |
| FAERS/AERS quarterly files | 2004Q1-2026Q2 | 90 quarters | — | — | — | — |
| Target-program candidate CASEIDs | 574,338 | after global latest-version/deletion handling; not the global FAERS total | — | — | — | — |
| Strict RCC treatment-context reports | 84,369 | strict indication-drug binding | — | — | — | — |
| Tier-A phenotype-positive reports | 2,307 | exclusive plus mixed | — | — | — | — |
| Immune-IS | 219 | exclusive | — | — | — | — |
| VEGF-axis | 1,076 | exclusive | — | — | — | — |
| Nonspecific AKI | 932 | exclusive | — | — | — | — |
| Mixed challenge | 80 | never entered training or tuning | — | — | — | — |
| JADER snapshot | 2004Q1-2026Q1 | 202607 release; terminal quarter contains 37 reports | — | — | — | — |
| JADER reports | 1,043,485 | snapshot total | — | — | — | — |
| Strict RCC primary-suspect reports | 16,572 | eligible reports | — | — | — | — |
| **B. T1 time-defined analysis sets** | | | | | | |
| historical pre2019 | 426 | — | 15 | 148 | 263 | 17 |
| development 2019Q1 2023Q4 | 961 | — | 136 | 437 | 388 | 39 |
| temporal validation 2024Q1 2025Q4 | 537 | — | 51 | 355 | 131 | 16 |
| locked audit 2026Q1 Q2 | 139 | — | 14 | 79 | 46 | 1 |
| missing init quarter | 164 | — | 3 | 57 | 104 | 7 |
| **C. Regimen-specific common-support starts** | | | | | | |
| Pembro + axitinib common support | 2019Q2 | FDA RCC approval 2019-04-19 | FAERS | — | — | — |
| Nivo + cabozantinib common support | 2021Q1 | FDA RCC approval 2021-01-22 | FAERS | — | — | — |
| Pembro + lenvatinib common support | 2021Q3 | FDA RCC approval 2021-08-10 | FAERS | — | — | — |
| Pembrolizumab + axitinib | 2019Q4 | Empirical JADER support start | JADER | — | — | — |
| Pembrolizumab + lenvatinib | 2022Q1 | Empirical JADER support start | JADER | — | — | — |
| Nivolumab + cabozantinib | 2021Q3 | Empirical JADER support start | JADER | — | — | — |

The 574,338 FAERS count is the prespecified target-program analysis-candidate subset after global latest-version and deletion handling; it is not the total number of reports in the complete FAERS universe. In section B the last four columns contain immune-interstitial, VEGF-axis, AKI, and mixed counts as indicated by the header sequence; missing INIT_FDA_DT reports were retained only in full-history sensitivity analyses.

*The 2026Q1–Q2 FAERS audit was descriptive because the immune class contained 14 reports. The JADER terminal 2026Q1 quarter contained 37 released reports and may be truncated.*

*Protocol-amendment register: covariate-adjusted Firth/Jeffreys-penalized models listed in protocol v1.0 were not transported because the available covariate-complete reconstruction did not reproduce the frozen nine primary cells; year-stratified MH-RORs remained the sole adjusted primary pharmacovigilance estimates.*

*AERS, Adverse Event Reporting System; AKI, acute kidney injury; FAERS, FDA Adverse Event Reporting System; FDA, US Food and Drug Administration; INIT_FDA_DT, initial FDA receipt date; JADER, Japanese Adverse Drug Event Report database; ML, machine learning; PS, primary suspect; RCC, renal cell carcinoma; VEGF, vascular endothelial growth factor.*

**Supplementary Table S2. Tier-A phenotype definitions and JADER cross-language mapping**

| **Phenotype** | **Scope/English gloss of JADER PT** | **Concept or exact-match expression** | **Status** | **JADER global n** | **JADER strict-RCC PS n** |
| --- | --- | --- | --- | --- | --- |
| **A. FAERS Tier-A definitions** | | | | | |
| Immune-IS | Five exact lexically constrained immune/interstitial nephritis PTs | ^(TUBULOINTERSTITIAL NEPHRITIS\|IMMUNE MEDIATED NEPHRITIS\|AUTOIMMUNE NEPHRITIS\|NEPHRITIS AUTOIMMUNE\|NEPHRITIS INTERSTITIAL)$ | Prespecified exact-term rule | — | — |
| VEGF-axis | Three exact terms for proteinuria, nephrotic syndrome, or renal-limited TMA | ^(PROTEINURIA\|NEPHROTIC SYNDROME\|RENAL LIMITED THROMBOTIC MICROANGIOPATHY)$ | Prespecified exact-term rule | — | — |
| Nonspecific AKI | Two exact nonspecific acute renal injury PTs | ^(ACUTE KIDNEY INJURY\|RENAL FAILURE ACUTE)$ | Prespecified exact-term rule | — | — |
| **B. JADER Japanese PT mapping** | | | | | |
| VEGF-axis | Nephrotic Syndrome | NEPHROTIC SYNDROME | Tier-A exact primary | 2,282 | 118 |
| Immune-IS | Immune Mediated Nephritis | IMMUNE MEDIATED NEPHRITIS | Tier-A exact primary | — | 0 |
| Immune-IS | Immune-Mediated Renal Disorder (Not Included in Tier-A) | IMMUNE-MEDIATED RENAL DISORDER (not included in Tier-A) | Broad semantic sensitivity only | 265 | 43 |
| Immune-IS | Tubulointerstitial/Interstitial Nephritis | TUBULOINTERSTITIAL/INTERSTITIAL NEPHRITIS | Tier-A exact primary | 3,605 | 133 |
| Nonspecific AKI | Acute Kidney Injury / Renal Failure Acute | ACUTE KIDNEY INJURY / RENAL FAILURE ACUTE | Tier-A exact primary | 11,696 | 171 |
| VEGF-axis | Renal Limited Thrombotic Microangiopathy | RENAL LIMITED THROMBOTIC MICROANGIOPATHY | Tier-A exact primary | 19 | 0 |
| Immune-IS | Autoimmune/Nephritis Autoimmune | AUTOIMMUNE/NEPHRITIS AUTOIMMUNE | Tier-A exact primary | 27 | 5 |
| VEGF-axis | Proteinuria | PROTEINURIA | Tier-A exact primary | 2,283 | 344 |

*Notes: The FAERS expressions in section A are the exact-match definitions after PT normalization to uppercase. Tier-A exclusive labels were frozen before supervised modeling. Mixed reports satisfied more than one Tier-A term family and never entered training, tuning, or threshold selection.*

*Broader protocol concepts and Tier-B PTs were sensitivity-only: they could not modify an exclusive Tier-A label or move a report between the three primary classes.*

*The JADER broad concept 'immune-mediated renal disorder' was used only in the expanded immune-semantic sensitivity analysis and not in the primary label definition.*

*Exact Japanese source PT strings are provided in data/table_source_data/S2_Phenotype_dictionary.csv in the Zenodo archive (https://doi.org/10.5281/zenodo.22807359). This table displays English glosses.*

*JADER, Japanese Adverse Drug Event Report database; PT, preferred term; PS, primary suspect; RCC, renal cell carcinoma.*

**Supplementary Table S3. Extended full-history FAERS report characteristics including the secondary sunitinib reference**

| **Characteristic** | **Pembrolizumab + axitinib** | **Pembrolizumab + lenvatinib** | **Nivolumab + cabozantinib** | **Nivolumab + ipilimumab** | **Sunitinib** |
| --- | --- | --- | --- | --- | --- |
| Reports, n | 2,446 | 5,656 | 5,649 | 4,566 | 21,890 |
| Report-year range | 2015–2026 | 2016–2026 | 2016–2026 | 2014–2026 | 2006–2026 |
| Age available, n (%) | 2,042 (83.5) | 4,160 (73.6) | 4,378 (77.5) | 3,590 (78.6) | 18,035 (82.4) |
| Age, median (IQR), years | 67 (60–74) | 68 (60–74) | 68 (60–75) | 66 (58–72) | 65 (57–72) |
| Female among known sex, n (%) | 635 (27.4) | 1,342 (25.0) | 1,404 (27.3) | 1,043 (25.9) | 6,400 (30.9) |
| HCP among known reporter status, n (%) | 1,874 (78.4) | 4,775 (87.8) | 3,526 (65.7) | 4,154 (92.1) | 13,122 (61.4) |
| Any serious outcome, n (%) | 1,248 (51.0) | 3,733 (66.0) | 1,899 (33.6) | 3,110 (68.1) | 12,422 (56.7) |
| Hospitalization, n (%) | 914 (37.4) | 3,233 (57.2) | 1,528 (27.0) | 2,403 (52.6) | 7,042 (32.2) |
| Death, n (%) | 389 (15.9) | 654 (11.6) | 488 (8.6) | 983 (21.5) | 6,512 (29.7) |
| Reported drugs, median (IQR) | 3 (2–6) | 3 (2–7) | 3 (2–9) | 2 (2–5) | 2 (1–5) |

*Notes: Sunitinib was retained only as a historical/mechanistic sensitivity comparator. Values are descriptive and not common-support adjusted. FAERS, FDA Adverse Event Reporting System; HCP, healthcare professional; IQR, interquartile range; RCC, renal cell carcinoma.*

Supplementary Table S4. ML cohort counts by analysis period and regimen-indicator pattern

| **Period / population** | **Report subset** | **Immune-IS** | **VEGF-axis** | **AKI** | **Exclusive n** | **Mixed n (separate)** |
| --- | --- | --- | --- | --- | --- | --- |
| Full history | Summary | 219 | 1076 | 932 | 2227 | 80 |
| Before 2019 | Excluded from temporal ML | 15 | 148 | 263 | 426 | 17 |
| Missing initial date | Excluded from temporal ML | 3 | 57 | 104 | 164 | 7 |
| Development 2019–2023 | Date-defined subset | 136 | 437 | 388 | 961 | 39 |
| Temporal 2024–2025 | Date-defined subset | 51 | 355 | 131 | 537 | 16 |
| Descriptive 2026 | Date-defined subset | 14 | 79 | 46 | 139 | 1 |
| Mixed challenge | Summary across all periods | — | — | — | 0 | 80 |

| **Date split** | **Indicator pattern** | **Immune-IS** | **VEGF-axis** | **AKI** | **Mixed** |
| --- | --- | --- | --- | --- | --- |
| Development | multiple indicators | 6 | 4 | 8 | 1 |
| Development | none of five | 66 | 223 | 161 | 13 |
| Development | N+Cabo | 1 | 34 | 40 | 4 |
| Development | N+Ipi | 37 | 5 | 83 | 9 |
| Development | P+Axi | 13 | 18 | 24 | 3 |
| Development | P+Lenva | 11 | 138 | 50 | 1 |
| Development | sunitinib | 2 | 15 | 22 | 8 |
| Pre-2019 | none of five | 9 | 82 | 141 | 10 |
| Pre-2019 | N+Ipi | 2 | 0 | 18 | 1 |
| Pre-2019 | P+Axi | 0 | 0 | 7 | 0 |
| Pre-2019 | P+Lenva | 0 | 0 | 4 | 0 |
| Pre-2019 | sunitinib | 4 | 66 | 93 | 6 |
| 2026 audit | none of five | 4 | 26 | 20 | 0 |
| 2026 audit | N+Cabo | 3 | 15 | 2 | 0 |
| 2026 audit | N+Ipi | 2 | 1 | 1 | 0 |
| 2026 audit | P+Axi | 3 | 0 | 5 | 1 |
| 2026 audit | P+Lenva | 2 | 34 | 18 | 0 |
| 2026 audit | sunitinib | 0 | 3 | 0 | 0 |
| Temporal | multiple indicators | 2 | 5 | 1 | 0 |
| Temporal | none of five | 17 | 140 | 50 | 7 |
| Temporal | N+Cabo | 5 | 41 | 28 | 1 |
| Temporal | N+Ipi | 12 | 1 | 11 | 3 |
| Temporal | P+Axi | 6 | 4 | 8 | 0 |
| Temporal | P+Lenva | 9 | 159 | 23 | 5 |
| Temporal | sunitinib | 0 | 5 | 10 | 0 |

The exclusive phenotype columns account for all 2,227 reports by date availability and analysis period: 961 development, 537 temporal-validation, 139 descriptive-audit, 426 pre-2019, and 164 missing-date reports. Mixed-report counts are shown separately and are excluded from the exclusive totals and all training and tuning. The full-history and mixed-challenge rows are summaries, not additional populations. Regimen-indicator patterns are shown for reports with usable dates. Exactly-one and multiple-indicator groups are separate; all-zero denotes heterogeneous remaining strict-RCC reports, not an omitted nivolumab–ipilimumab category. Suspect-role indicators are multi-hot. The sunitinib indicator excludes suspect pembrolizumab, nivolumab, and ipilimumab. Missing-date reports have no temporal-matrix rows, so their regimen-indicator patterns are not tabulated. Serious-outcome summaries are provided in Table 1 and Figure S3.

**Supplementary Table S5. Annual 2 × 2 strata underlying the primary FAERS Mantel–Haenszel estimates**

| **Target regimen** | **Phenotype** | **Year** | **Target event** | **Target non-event** | **Comparator event** | **Comparator non-event** |
| --- | --- | --- | --- | --- | --- | --- |
| Pembrolizumab + axitinib | Immune-IS | 2019 | 0 | 56 | 5 | 680 |
| Pembrolizumab + axitinib | Immune-IS | 2020 | 2 | 213 | 8 | 787 |
| Pembrolizumab + axitinib | Immune-IS | 2021 | 2 | 289 | 7 | 639 |
| Pembrolizumab + axitinib | Immune-IS | 2022 | 5 | 335 | 9 | 531 |
| Pembrolizumab + axitinib | Immune-IS | 2023 | 4 | 263 | 7 | 365 |
| Pembrolizumab + axitinib | Immune-IS | 2024 | 3 | 202 | 11 | 311 |
| Pembrolizumab + axitinib | Immune-IS | 2025 | 3 | 109 | 1 | 306 |
| Pembrolizumab + axitinib | Immune-IS | 2026 | 3 | 67 | 2 | 110 |
| Pembrolizumab + axitinib | VEGF-axis | 2019 | 0 | 56 | 1 | 684 |
| Pembrolizumab + axitinib | VEGF-axis | 2020 | 2 | 213 | 0 | 795 |
| Pembrolizumab + axitinib | VEGF-axis | 2021 | 3 | 288 | 0 | 646 |
| Pembrolizumab + axitinib | VEGF-axis | 2022 | 9 | 331 | 2 | 538 |
| Pembrolizumab + axitinib | VEGF-axis | 2023 | 4 | 263 | 2 | 370 |
| Pembrolizumab + axitinib | VEGF-axis | 2024 | 3 | 202 | 0 | 322 |
| Pembrolizumab + axitinib | VEGF-axis | 2025 | 1 | 111 | 1 | 306 |
| Pembrolizumab + axitinib | VEGF-axis | 2026 | 0 | 70 | 1 | 111 |
| Pembrolizumab + axitinib | Nonspecific AKI | 2019 | 1 | 55 | 16 | 669 |
| Pembrolizumab + axitinib | Nonspecific AKI | 2020 | 10 | 205 | 21 | 774 |
| Pembrolizumab + axitinib | Nonspecific AKI | 2021 | 7 | 284 | 21 | 625 |
| Pembrolizumab + axitinib | Nonspecific AKI | 2022 | 4 | 336 | 17 | 523 |
| Pembrolizumab + axitinib | Nonspecific AKI | 2023 | 2 | 265 | 4 | 368 |
| Pembrolizumab + axitinib | Nonspecific AKI | 2024 | 2 | 203 | 7 | 315 |
| Pembrolizumab + axitinib | Nonspecific AKI | 2025 | 6 | 106 | 4 | 303 |
| Pembrolizumab + axitinib | Nonspecific AKI | 2026 | 5 | 65 | 1 | 111 |
| Pembrolizumab + lenvatinib | Immune-IS | 2021 | 0 | 97 | 4 | 335 |
| Pembrolizumab + lenvatinib | Immune-IS | 2022 | 5 | 784 | 9 | 531 |
| Pembrolizumab + lenvatinib | Immune-IS | 2023 | 6 | 1383 | 7 | 365 |
| Pembrolizumab + lenvatinib | Immune-IS | 2024 | 6 | 886 | 11 | 311 |
| Pembrolizumab + lenvatinib | Immune-IS | 2025 | 3 | 883 | 1 | 306 |
| Pembrolizumab + lenvatinib | Immune-IS | 2026 | 2 | 416 | 2 | 110 |
| Pembrolizumab + lenvatinib | VEGF-axis | 2021 | 0 | 97 | 0 | 339 |
| Pembrolizumab + lenvatinib | VEGF-axis | 2022 | 44 | 745 | 2 | 538 |
| Pembrolizumab + lenvatinib | VEGF-axis | 2023 | 93 | 1296 | 2 | 370 |
| Pembrolizumab + lenvatinib | VEGF-axis | 2024 | 61 | 831 | 0 | 322 |
| Pembrolizumab + lenvatinib | VEGF-axis | 2025 | 98 | 788 | 1 | 306 |
| Pembrolizumab + lenvatinib | VEGF-axis | 2026 | 34 | 384 | 1 | 111 |
| Pembrolizumab + lenvatinib | Nonspecific AKI | 2021 | 1 | 96 | 13 | 326 |
| Pembrolizumab + lenvatinib | Nonspecific AKI | 2022 | 20 | 769 | 17 | 523 |
| Pembrolizumab + lenvatinib | Nonspecific AKI | 2023 | 22 | 1367 | 4 | 368 |
| Pembrolizumab + lenvatinib | Nonspecific AKI | 2024 | 18 | 874 | 7 | 315 |
| Pembrolizumab + lenvatinib | Nonspecific AKI | 2025 | 5 | 881 | 4 | 303 |
| Pembrolizumab + lenvatinib | Nonspecific AKI | 2026 | 18 | 400 | 1 | 111 |
| Nivolumab + cabozantinib | Immune-IS | 2021 | 0 | 239 | 7 | 639 |
| Nivolumab + cabozantinib | Immune-IS | 2022 | 0 | 629 | 9 | 531 |
| Nivolumab + cabozantinib | Immune-IS | 2023 | 1 | 1123 | 7 | 365 |
| Nivolumab + cabozantinib | Immune-IS | 2024 | 0 | 1130 | 11 | 311 |
| Nivolumab + cabozantinib | Immune-IS | 2025 | 5 | 1111 | 1 | 306 |
| Nivolumab + cabozantinib | Immune-IS | 2026 | 3 | 673 | 2 | 110 |
| Nivolumab + cabozantinib | VEGF-axis | 2021 | 1 | 238 | 0 | 646 |
| Nivolumab + cabozantinib | VEGF-axis | 2022 | 7 | 622 | 2 | 538 |
| Nivolumab + cabozantinib | VEGF-axis | 2023 | 24 | 1100 | 2 | 370 |
| Nivolumab + cabozantinib | VEGF-axis | 2024 | 16 | 1114 | 0 | 322 |
| Nivolumab + cabozantinib | VEGF-axis | 2025 | 25 | 1091 | 1 | 306 |
| Nivolumab + cabozantinib | VEGF-axis | 2026 | 15 | 661 | 1 | 111 |
| Nivolumab + cabozantinib | Nonspecific AKI | 2021 | 6 | 233 | 21 | 625 |
| Nivolumab + cabozantinib | Nonspecific AKI | 2022 | 13 | 616 | 17 | 523 |
| Nivolumab + cabozantinib | Nonspecific AKI | 2023 | 19 | 1105 | 4 | 368 |
| Nivolumab + cabozantinib | Nonspecific AKI | 2024 | 9 | 1121 | 7 | 315 |
| Nivolumab + cabozantinib | Nonspecific AKI | 2025 | 19 | 1097 | 4 | 303 |
| Nivolumab + cabozantinib | Nonspecific AKI | 2026 | 2 | 674 | 1 | 111 |

*Notes: Counts are report-level annual strata after regimen-specific common-support restriction. The comparator was nivolumab plus ipilimumab. AKI, acute kidney injury; FAERS, FDA Adverse Event Reporting System; MH, Mantel–Haenszel; RCC, renal cell carcinoma; VEGF, vascular endothelial growth factor.*

**Supplementary Table S6. Prespecified FAERS HCP-only and regimen-overlap sensitivity analyses**

| **Target regimen** | **Phenotype** | **Specification** | **MH-ROR (95% CI)** | **Primary BH q** | **Direction** |
| --- | --- | --- | --- | --- | --- |
| Pembrolizumab + axitinib | Immune-IS | Primary exclusive | 0.92 (0.55–1.52) | 0.790 | <1 |
| Pembrolizumab + axitinib | Immune-IS | HCP-only | 1.03 (0.61–1.74) | — | >1 |
| Pembrolizumab + axitinib | Immune-IS | Regimen overlap allowed | 0.92 (0.56–1.51) | — | <1 |
| Pembrolizumab + axitinib | VEGF-axis | Primary exclusive | 5.31 (2.43–11.59) | 6.18e-05 | >1 |
| Pembrolizumab + axitinib | VEGF-axis | HCP-only | 5.06 (2.24–11.41) | — | >1 |
| Pembrolizumab + axitinib | VEGF-axis | Regimen overlap allowed | 3.60 (1.90–6.80) | — | >1 |
| Pembrolizumab + axitinib | Nonspecific AKI | Primary exclusive | 1.05 (0.71–1.56) | 0.790 | >1 |
| Pembrolizumab + axitinib | Nonspecific AKI | HCP-only | 1.29 (0.86–1.92) | — | >1 |
| Pembrolizumab + axitinib | Nonspecific AKI | Regimen overlap allowed | 1.14 (0.78–1.67) | — | >1 |
| Pembrolizumab + lenvatinib | Immune-IS | Primary exclusive | 0.29 (0.16–0.50) | 3.09e-05 | <1 |
| Pembrolizumab + lenvatinib | Immune-IS | HCP-only | 0.33 (0.19–0.57) | — | <1 |
| Pembrolizumab + lenvatinib | Immune-IS | Regimen overlap allowed | 0.32 (0.18–0.55) | — | <1 |
| Pembrolizumab + lenvatinib | VEGF-axis | Primary exclusive | 19.48 (9.03–42.01) | 3.32e-13 | >1 |
| Pembrolizumab + lenvatinib | VEGF-axis | HCP-only | 22.02 (9.92–48.88) | — | >1 |
| Pembrolizumab + lenvatinib | VEGF-axis | Regimen overlap allowed | 11.67 (6.71–20.31) | — | >1 |
| Pembrolizumab + lenvatinib | Nonspecific AKI | Primary exclusive | 0.93 (0.63–1.39) | 0.790 | <1 |
| Pembrolizumab + lenvatinib | Nonspecific AKI | HCP-only | 0.94 (0.62–1.43) | — | <1 |
| Pembrolizumab + lenvatinib | Nonspecific AKI | Regimen overlap allowed | 0.99 (0.67–1.44) | — | <1 |
| Nivolumab + cabozantinib | Immune-IS | Primary exclusive | 0.10 (0.05–0.22) | 3.27e-08 | <1 |
| Nivolumab + cabozantinib | Immune-IS | HCP-only | 0.15 (0.06–0.33) | — | <1 |
| Nivolumab + cabozantinib | Immune-IS | Regimen overlap allowed | 0.18 (0.10–0.35) | — | <1 |
| Nivolumab + cabozantinib | VEGF-axis | Primary exclusive | 4.59 (2.07–10.19) | 3.13e-04 | >1 |
| Nivolumab + cabozantinib | VEGF-axis | HCP-only | 7.03 (3.03–16.30) | — | >1 |
| Nivolumab + cabozantinib | VEGF-axis | Regimen overlap allowed | 2.73 (1.53–4.89) | — | >1 |
| Nivolumab + cabozantinib | Nonspecific AKI | Primary exclusive | 0.78 (0.53–1.17) | 0.349 | <1 |
| Nivolumab + cabozantinib | Nonspecific AKI | HCP-only | 1.20 (0.78–1.83) | — | >1 |
| Nivolumab + cabozantinib | Nonspecific AKI | Regimen overlap allowed | 0.78 (0.53–1.12) | — | <1 |

*Notes: The comparator was nivolumab plus ipilimumab. Multiplicity-adjusted q values apply to the nine primary cells; sensitivity estimates are interpreted by architecture and interval stability rather than as independent tests. AKI, acute kidney injury; BH, Benjamini–Hochberg; CI, confidence interval; HCP, healthcare professional; MH-ROR, Mantel–Haenszel reporting odds ratio; VEGF, vascular endothelial growth factor.*

**Supplementary Table S7. Sunitinib historical/mechanistic comparator sensitivity analysis**

| **Target regimen** | **Phenotype** | **Support start** | **vs sunitinib MH-ROR (95% CI)** | **BH q** | **vs nivo + ipi MH-ROR (95% CI)** | **Same direction** | **Interpretive class** |
| --- | --- | --- | --- | --- | --- | --- | --- |
| Pembrolizumab + axitinib | Immune-IS | 2019Q2 | 8.97 (3.21–25.11) | 1.12e-04 | 0.92 (0.55–1.52) | No | strong concordance |
| Pembrolizumab + axitinib | VEGF-axis | 2019Q2 | 1.97 (1.06–3.66) | 0.059 | 5.31 (2.43–11.59) | Yes | substantive estimand divergence |
| Pembrolizumab + axitinib | Nonspecific AKI | 2019Q2 | 3.05 (1.80–5.19) | 1.12e-04 | 1.05 (0.71–1.56) | Yes | substantive estimand divergence |
| Pembrolizumab + lenvatinib | Immune-IS | 2021Q3 | 2.68 (0.78–9.24) | 0.152 | 0.29 (0.16–0.50) | No | substantive estimand divergence |
| Pembrolizumab + lenvatinib | VEGF-axis | 2021Q3 | 6.56 (3.81–11.32) | 1.21e-10 | 19.48 (9.03–42.01) | Yes | strong concordance |
| Pembrolizumab + lenvatinib | Nonspecific AKI | 2021Q3 | 1.88 (1.06–3.35) | 0.059 | 0.93 (0.63–1.39) | No | substantive estimand divergence |
| Nivolumab + cabozantinib | Immune-IS | 2021Q1 | 0.88 (0.24–3.18) | 0.848 | 0.10 (0.05–0.22) | Yes | near-null estimand divergence |
| Nivolumab + cabozantinib | VEGF-axis | 2021Q1 | 1.51 (0.84–2.71) | 0.186 | 4.59 (2.07–10.19) | Yes | directional concordance / near-null |
| Nivolumab + cabozantinib | Nonspecific AKI | 2021Q1 | 1.67 (0.95–2.93) | 0.116 | 0.78 (0.53–1.17) | No | substantive estimand divergence |

*Notes: Sunitinib crossed treatment eras and occupied a VEGF-mechanism position; it was therefore prespecified as secondary sensitivity evidence, not the primary modern active comparator. AKI, acute kidney injury; BH, Benjamini–Hochberg; CI, confidence interval; MH-ROR, Mantel–Haenszel reporting odds ratio; VEGF, vascular endothelial growth factor.*

**Supplementary Table S8. Anchor-exclusion audit and complete feature manifest**

| **Model scope** | **Feature/action** | **Family/count** | **Disposition** | **Encoding/transform** | **Freeze reason** |
| --- | --- | --- | --- | --- | --- |
| **A. T1 feature-audit disposition** | | | | | |
| M1+M2 | BLACKLIST_LEAKAGE | 19 | — | — | — |
| M1+M2 | EXCLUDE_PRIMARY | 1 | — | — | — |
| M1+M2 | EXCLUDE_V1 | 1 | — | — | — |
| M1+M2 | KEEP | 55 | — | — | — |
| M1+M2 | SENSITIVITY_ONLY | 2 | — | — | — |
| M2_only | KEEP | 5 | — | — | — |
| **B. Complete feature manifest** | | | | | |
| M1+M2 | age_years | demographic | KEEP | continuous years; valid 0-120 | Pre-event/report-level demographic |
| M1+M2 | age_missing | demographic | KEEP | binary 0/1 | Non-anchor report-level feature |
| M1+M2 | sex_male | demographic | KEEP | binary 0/1 | Non-anchor report-level feature |
| M1+M2 | sex_female | demographic | KEEP | binary 0/1 | Non-anchor report-level feature |
| M1+M2 | sex_missing | demographic | KEEP | binary 0/1 | Non-anchor report-level feature |
| M1+M2 | hcp_flag | reporter | KEEP | binary 0/1 | Non-anchor report-level feature |
| M1+M2 | reporter_prof_missing | reporter | KEEP | binary 0/1 | Non-anchor report-level feature |
| M1+M2 | country_US | reporter | KEEP | binary 0/1 | Non-anchor report-level feature |
| M1+M2 | country_JP | reporter | KEEP | binary 0/1 | Non-anchor report-level feature |
| M1+M2 | country_CA | reporter | KEEP | binary 0/1 | Non-anchor report-level feature |
| M1+M2 | country_UK | reporter | KEEP | binary 0/1 | Non-anchor report-level feature |
| M1+M2 | country_EU_EEA | reporter | KEEP | binary 0/1 | Non-anchor report-level feature |
| M1+M2 | country_OTHER | reporter | KEEP | binary 0/1 | Non-anchor report-level feature |
| M1+M2 | country_MISSING | reporter | KEEP | binary 0/1 | Non-anchor report-level feature |
| M1+M2 | serious | outcome | KEEP | binary 0/1 | Non-anchor report-level feature |
| M1+M2 | death | outcome | KEEP | binary 0/1 | Non-anchor report-level feature |
| M1+M2 | hospitalization | outcome | KEEP | binary 0/1 | Non-anchor report-level feature |
| M1+M2 | life_threatening | outcome | KEEP | binary 0/1 | Non-anchor report-level feature |
| M1+M2 | disability | outcome | KEEP | binary 0/1 | Non-anchor report-level feature |
| M1+M2 | tto_missing | timing | SENSITIVITY_ONLY | binary 0/1 | TTO missingness is high and drifts from 48.8% in development to 61.6% in temporal validation; preserve for sensitivity/characterization, not primary classifier. |
| M1+M2 | tto_days | timing | SENSITIVITY_ONLY | continuous days 0-3650 | TTO missingness is high and drifts from 48.8% in development to 61.6% in temporal validation; preserve for sensitivity/characterization, not primary classifier. |
| M1+M2 | PT:HYPERTENSION | co-reported_PT | KEEP | binary reported/not-reported | Development-only overall frequency >=20; leakage blacklist passed |
| M1+M2 | PT:DIARRHOEA | co-reported_PT | KEEP | binary reported/not-reported | Development-only overall frequency >=20; leakage blacklist passed |
| M1+M2 | PT:PALMAR PLANTAR ERYTHRODYSAESTHESIA SYNDROME | co-reported_PT | KEEP | binary reported/not-reported | Development-only overall frequency >=20; leakage blacklist passed |
| M1+M2 | PT:MALIGNANT NEOPLASM PROGRESSION | co-reported_PT | KEEP | binary reported/not-reported | Development-only overall frequency >=20; leakage blacklist passed |
| M1+M2 | PT:HYPOTHYROIDISM | co-reported_PT | KEEP | binary reported/not-reported | Development-only overall frequency >=20; leakage blacklist passed |
| M1+M2 | PT:DECREASED APPETITE | co-reported_PT | KEEP | binary reported/not-reported | Development-only overall frequency >=20; leakage blacklist passed |
| M1+M2 | PT:FATIGUE | co-reported_PT | KEEP | binary reported/not-reported | Development-only overall frequency >=20; leakage blacklist passed |
| M1+M2 | PT:MALAISE | co-reported_PT | KEEP | binary reported/not-reported | Development-only overall frequency >=20; leakage blacklist passed |
| M1+M2 | PT:NAUSEA | co-reported_PT | KEEP | binary reported/not-reported | Development-only overall frequency >=20; leakage blacklist passed |
| M1+M2 | PT:PYREXIA | co-reported_PT | KEEP | binary reported/not-reported | Development-only overall frequency >=20; leakage blacklist passed |
| M1+M2 | PT:DEHYDRATION | co-reported_PT | KEEP | binary reported/not-reported | Development-only overall frequency >=20; leakage blacklist passed |
| M1+M2 | PT:PLATELET COUNT DECREASED | co-reported_PT | KEEP | binary reported/not-reported | Development-only overall frequency >=20; leakage blacklist passed |
| M1+M2 | PT:ANAEMIA | co-reported_PT | KEEP | binary reported/not-reported | Development-only overall frequency >=20; leakage blacklist passed |
| M1+M2 | PT:OEDEMA PERIPHERAL | co-reported_PT | KEEP | binary reported/not-reported | Development-only overall frequency >=20; leakage blacklist passed |
| M1+M2 | PT:HEPATIC FUNCTION ABNORMAL | co-reported_PT | KEEP | binary reported/not-reported | Development-only overall frequency >=20; leakage blacklist passed |
| M1+M2 | PT:WEIGHT DECREASED | co-reported_PT | KEEP | binary reported/not-reported | Development-only overall frequency >=20; leakage blacklist passed |
| M1+M2 | PT:RASH | co-reported_PT | KEEP | binary reported/not-reported | Development-only overall frequency >=20; leakage blacklist passed |
| M1+M2 | PT:BLOOD CREATININE INCREASED | co-reported_PT | KEEP | binary reported/not-reported | Development-only overall frequency >=20; leakage blacklist passed |
| M1+M2 | PT:DYSPNOEA | co-reported_PT | KEEP | binary reported/not-reported | Development-only overall frequency >=20; leakage blacklist passed |
| M1+M2 | PT:SEPSIS | co-reported_PT | KEEP | binary reported/not-reported | Development-only overall frequency >=20; leakage blacklist passed |
| M1+M2 | PT:VOMITING | co-reported_PT | KEEP | binary reported/not-reported | Development-only overall frequency >=20; leakage blacklist passed |
| M1+M2 | PT:HYPERKALAEMIA | co-reported_PT | KEEP | binary reported/not-reported | Development-only overall frequency >=20; leakage blacklist passed |
| M1+M2 | PT:STOMATITIS | co-reported_PT | KEEP | binary reported/not-reported | Development-only overall frequency >=20; leakage blacklist passed |
| M1+M2 | PT:DYSPHONIA | co-reported_PT | KEEP | binary reported/not-reported | Development-only overall frequency >=20; leakage blacklist passed |
| M1+M2 | PT:OEDEMA | co-reported_PT | KEEP | binary reported/not-reported | Development-only overall frequency >=20; leakage blacklist passed |
| M1+M2 | PT:ASTHENIA | co-reported_PT | KEEP | binary reported/not-reported | Development-only overall frequency >=20; leakage blacklist passed |
| M1+M2 | PT:PNEUMONIA | co-reported_PT | KEEP | binary reported/not-reported | Development-only overall frequency >=20; leakage blacklist passed |
| M1+M2 | PT:ADRENAL INSUFFICIENCY | co-reported_PT | KEEP | binary reported/not-reported | Development-only overall frequency >=20; leakage blacklist passed |
| M1+M2 | PT:CARDIAC FAILURE | co-reported_PT | KEEP | binary reported/not-reported | Development-only overall frequency >=20; leakage blacklist passed |
| M1+M2 | PT:BLOOD PRESSURE INCREASED | co-reported_PT | KEEP | binary reported/not-reported | Development-only overall frequency >=20; leakage blacklist passed |
| M1+M2 | PT:PLEURAL EFFUSION | co-reported_PT | KEEP | binary reported/not-reported | Development-only overall frequency >=20; leakage blacklist passed |
| M1+M2 | PT:OFF LABEL USE | co-reported_PT | KEEP | binary reported/not-reported | Development-only overall frequency >=20; leakage blacklist passed |
| M1+M2 | PT:GENERAL PHYSICAL HEALTH DETERIORATION | co-reported_PT | KEEP | binary reported/not-reported | Development-only overall frequency >=20; leakage blacklist passed |
| M1+M2 | PT:TASTE DISORDER | co-reported_PT | KEEP | binary reported/not-reported | Development-only overall frequency >=20; leakage blacklist passed |
| M1+M2 | PT:ASCITES | co-reported_PT | KEEP | binary reported/not-reported | Development-only overall frequency >=20; leakage blacklist passed |
| M1+M2 | PT:METABOLIC ACIDOSIS | co-reported_PT | KEEP | binary reported/not-reported | Development-only overall frequency >=20; leakage blacklist passed |
| M2_only | reg_pembro_axitinib | regimen | KEEP | binary multi-hot | Treatment-regimen increment over anchor-excluded M1 |
| M2_only | reg_pembro_lenvatinib | regimen | KEEP | binary multi-hot | Treatment-regimen increment over anchor-excluded M1 |
| M2_only | reg_nivo_cabozantinib | regimen | KEEP | binary multi-hot | Treatment-regimen increment over anchor-excluded M1 |
| M2_only | reg_nivo_ipilimumab | regimen | KEEP | binary multi-hot | Treatment-regimen increment over anchor-excluded M1 |
| M2_only | reg_sunitinib_reference | regimen | KEEP | binary multi-hot | Treatment-regimen increment over anchor-excluded M1 |
| M1+M2 | calendar_year_or_quarter | calendar | EXCLUDE_PRIMARY | split/audit variable only | Would undermine temporal transportability test if used as predictor |
| M1+M2 | concomitant_drug_architecture | medication | EXCLUDE_V1 |  | The source extract included only target/proxy drugs. Complete pre-event concomitant medication histories were unavailable, and post-event rescue treatment could introduce leakage. |
| M1+M2 | PT:ACUTE KIDNEY INJURY | co-reported_PT | BLACKLIST_LEAKAGE |  | Direct renal class anchor/synonym |
| M1+M2 | PT:AUTOIMMUNE NEPHRITIS | co-reported_PT | BLACKLIST_LEAKAGE |  | Direct renal class anchor/synonym |
| M1+M2 | PT:GLOMERULONEPHRITIS | co-reported_PT | BLACKLIST_LEAKAGE |  | Direct renal class anchor/synonym |
| M1+M2 | PT:GLOMERULONEPHRITIS ACUTE | co-reported_PT | BLACKLIST_LEAKAGE |  | Direct renal class anchor/synonym |
| M1+M2 | PT:GLOMERULONEPHRITIS RAPIDLY PROGRESSIVE | co-reported_PT | BLACKLIST_LEAKAGE |  | Direct renal class anchor/synonym |
| M1+M2 | PT:IMMUNE MEDIATED NEPHRITIS | co-reported_PT | BLACKLIST_LEAKAGE |  | Direct renal class anchor/synonym |
| M1+M2 | PT:MICROANGIOPATHY | co-reported_PT | BLACKLIST_LEAKAGE |  | Direct renal class anchor/synonym |
| M1+M2 | PT:NEPHRITIC SYNDROME | co-reported_PT | BLACKLIST_LEAKAGE |  | Direct renal class anchor/synonym |
| M1+M2 | PT:NEPHRITIS | co-reported_PT | BLACKLIST_LEAKAGE |  | Direct renal class anchor/synonym |
| M1+M2 | PT:NEPHRITIS AUTOIMMUNE | co-reported_PT | BLACKLIST_LEAKAGE |  | Direct renal class anchor/synonym |
| M1+M2 | PT:NEPHRITIS INTERSTITIAL | co-reported_PT | BLACKLIST_LEAKAGE |  | Direct renal class anchor/synonym |
| M1+M2 | PT:NEPHROTIC SYNDROME | co-reported_PT | BLACKLIST_LEAKAGE |  | Direct renal class anchor/synonym |
| M1+M2 | PT:PROTEINURIA | co-reported_PT | BLACKLIST_LEAKAGE |  | Direct renal class anchor/synonym |
| M1+M2 | PT:RENAL FAILURE | co-reported_PT | BLACKLIST_LEAKAGE |  | Direct renal class anchor/synonym |
| M1+M2 | PT:RENAL FAILURE ACUTE | co-reported_PT | BLACKLIST_LEAKAGE |  | Direct renal class anchor/synonym |
| M1+M2 | PT:RENAL IMPAIRMENT | co-reported_PT | BLACKLIST_LEAKAGE |  | Direct renal class anchor/synonym |
| M1+M2 | PT:RENAL LIMITED THROMBOTIC MICROANGIOPATHY | co-reported_PT | BLACKLIST_LEAKAGE |  | Direct renal class anchor/synonym |
| M1+M2 | PT:THROMBOTIC MICROANGIOPATHY | co-reported_PT | BLACKLIST_LEAKAGE |  | Direct renal class anchor/synonym |
| M1+M2 | PT:TUBULOINTERSTITIAL NEPHRITIS | co-reported_PT | BLACKLIST_LEAKAGE |  | Direct renal class anchor/synonym |

Notes: M1 contained 55 anchor-excluded predictors. M2 added only five prespecified regimen indicators. TTO variables were sensitivity-only because missingness increased over calendar time. Tier-A anchor terms and their direct semantic families were excluded from supervised predictors. M1, phenotype-only classifier; M2, M1 plus treatment-regimen indicators; ML, machine learning; PT, preferred term; T1, three-class renal-phenotype task; TTO, time to onset.

**Supplementary Table S9. Complete temporal-validation discrimination, calibration, and confusion-matrix metrics**

| **Model** | **Class or cell** | **n** | AP/macro-AUPRC | **AUROC/macro-AUROC** | **Sensitivity/balanced accuracy** | **Precision/macro-F1** | **Brier** | **Log loss or calibration intercept/slope or row proportion** |
| --- | --- | --- | --- | --- | --- | --- | --- | --- |
| **A. Aggregate temporal-validation performance** | | | | | | | | |
| M1 multinomial elastic-net | All classes | 537 | 0.606 | 0.822 | 0.559 | 0.554 | 0.417 | 0.735 |
| M2 multinomial elastic-net + regimen | All classes | 537 | 0.638 | 0.840 | 0.609 | 0.601 | 0.383 | 0.686 |
| XGBoost challenger | All classes | 537 | 0.625 | 0.840 | 0.612 | 0.607 | 0.347 | 0.624 |
| LightGBM challenger | All classes | 537 | 0.634 | 0.841 | 0.598 | 0.595 | 0.339 | 0.613 |
| **B. Class-specific discrimination, classification, and calibration** | | | | | | | | |
| M1 multinomial elastic-net | Immune-IS | — | 0.175 | 0.697 | 0.176 | 0.120 | 0.125 | -1.353 / 1.098 |
| M1 multinomial elastic-net | VEGF-axis | — | 0.944 | 0.900 | 0.828 | 0.913 | 0.167 | 1.564 / 1.808 |
| M1 multinomial elastic-net | Nonspecific AKI | — | 0.699 | 0.870 | 0.672 | 0.629 | 0.125 | 0.459 / 1.627 |
| M2 multinomial elastic-net + regimen | Immune-IS | — | 0.253 | 0.742 | 0.294 | 0.231 | 0.109 | -1.063 / 1.289 |
| M2 multinomial elastic-net + regimen | VEGF-axis | — | 0.946 | 0.904 | 0.854 | 0.899 | 0.151 | 1.270 / 1.764 |
| M2 multinomial elastic-net + regimen | Nonspecific AKI | — | 0.717 | 0.874 | 0.679 | 0.659 | 0.123 | 0.452 / 1.615 |
| XGBoost challenger | Immune-IS | — | 0.219 | 0.737 | 0.314 | 0.250 | 0.103 | -1.315 / 0.710 |
| XGBoost challenger | VEGF-axis | — | 0.943 | 0.902 | 0.873 | 0.896 | 0.127 | 0.818 / 1.111 |
| XGBoost challenger | Nonspecific AKI | — | 0.713 | 0.880 | 0.649 | 0.669 | 0.117 | 0.156 / 1.095 |
| LightGBM challenger | Immune-IS | — | 0.237 | 0.741 | 0.294 | 0.250 | 0.100 | -1.336 / 0.605 |
| LightGBM challenger | VEGF-axis | — | 0.938 | 0.899 | 0.868 | 0.888 | 0.125 | 0.698 / 0.993 |
| LightGBM challenger | Nonspecific AKI | — | 0.726 | 0.883 | 0.634 | 0.638 | 0.115 | 0.108 / 1.023 |
| **C. Row-normalized confusion-matrix cells** | | | | | | | | |
| M1 multinomial elastic-net | Immune-IS → Immune-IS | 9 | — | — | — | — | — | 0.176 |
| M1 multinomial elastic-net | Immune-IS → VEGF-axis | 16 | — | — | — | — | — | 0.314 |
| M1 multinomial elastic-net | Immune-IS → Nonspecific AKI | 26 | — | — | — | — | — | 0.510 |
| M1 multinomial elastic-net | VEGF-axis → Immune-IS | 35 | — | — | — | — | — | 0.099 |
| M1 multinomial elastic-net | VEGF-axis → VEGF-axis | 294 | — | — | — | — | — | 0.828 |
| M1 multinomial elastic-net | VEGF-axis → Nonspecific AKI | 26 | — | — | — | — | — | 0.073 |
| M1 multinomial elastic-net | Nonspecific AKI → Immune-IS | 31 | — | — | — | — | — | 0.237 |
| M1 multinomial elastic-net | Nonspecific AKI → VEGF-axis | 12 | — | — | — | — | — | 0.092 |
| M1 multinomial elastic-net | Nonspecific AKI → Nonspecific AKI | 88 | — | — | — | — | — | 0.672 |
| M2 multinomial elastic-net + regimen | Immune-IS → Immune-IS | 15 | — | — | — | — | — | 0.294 |
| M2 multinomial elastic-net + regimen | Immune-IS → VEGF-axis | 15 | — | — | — | — | — | 0.294 |
| M2 multinomial elastic-net + regimen | Immune-IS → Nonspecific AKI | 21 | — | — | — | — | — | 0.412 |
| M2 multinomial elastic-net + regimen | VEGF-axis → Immune-IS | 27 | — | — | — | — | — | 0.076 |
| M2 multinomial elastic-net + regimen | VEGF-axis → VEGF-axis | 303 | — | — | — | — | — | 0.854 |
| M2 multinomial elastic-net + regimen | VEGF-axis → Nonspecific AKI | 25 | — | — | — | — | — | 0.070 |
| M2 multinomial elastic-net + regimen | Nonspecific AKI → Immune-IS | 23 | — | — | — | — | — | 0.176 |
| M2 multinomial elastic-net + regimen | Nonspecific AKI → VEGF-axis | 19 | — | — | — | — | — | 0.145 |
| M2 multinomial elastic-net + regimen | Nonspecific AKI → Nonspecific AKI | 89 | — | — | — | — | — | 0.679 |
| XGBoost challenger | Immune-IS → Immune-IS | 16 | — | — | — | — | — | 0.314 |
| XGBoost challenger | Immune-IS → VEGF-axis | 15 | — | — | — | — | — | 0.294 |
| XGBoost challenger | Immune-IS → Nonspecific AKI | 20 | — | — | — | — | — | 0.392 |
| XGBoost challenger | VEGF-axis → Immune-IS | 23 | — | — | — | — | — | 0.065 |
| XGBoost challenger | VEGF-axis → VEGF-axis | 310 | — | — | — | — | — | 0.873 |
| XGBoost challenger | VEGF-axis → Nonspecific AKI | 22 | — | — | — | — | — | 0.062 |
| XGBoost challenger | Nonspecific AKI → Immune-IS | 25 | — | — | — | — | — | 0.191 |
| XGBoost challenger | Nonspecific AKI → VEGF-axis | 21 | — | — | — | — | — | 0.160 |
| XGBoost challenger | Nonspecific AKI → Nonspecific AKI | 85 | — | — | — | — | — | 0.649 |
| LightGBM challenger | Immune-IS → Immune-IS | 15 | — | — | — | — | — | 0.294 |
| LightGBM challenger | Immune-IS → VEGF-axis | 14 | — | — | — | — | — | 0.275 |
| LightGBM challenger | Immune-IS → Nonspecific AKI | 22 | — | — | — | — | — | 0.431 |
| LightGBM challenger | VEGF-axis → Immune-IS | 22 | — | — | — | — | — | 0.062 |
| LightGBM challenger | VEGF-axis → VEGF-axis | 308 | — | — | — | — | — | 0.868 |
| LightGBM challenger | VEGF-axis → Nonspecific AKI | 25 | — | — | — | — | — | 0.070 |
| LightGBM challenger | Nonspecific AKI → Immune-IS | 23 | — | — | — | — | — | 0.176 |
| LightGBM challenger | Nonspecific AKI → VEGF-axis | 25 | — | — | — | — | — | 0.191 |
| LightGBM challenger | Nonspecific AKI → Nonspecific AKI | 83 | — | — | — | — | — | 0.634 |

*Notes: All metrics use the 2024–2025 temporal validation set (n=537). Calibration intercept and slope are one-versus-rest. Development data were used for fitting and tuning; this temporal set also informed model-family comparison. AKI, acute kidney injury; AP, average precision; AUPRC, area under the precision–recall curve; AUROC, area under the receiver-operating-characteristic curve; M1, phenotype-only classifier; M2, M1 plus treatment-regimen indicators; VEGF, vascular endothelial growth factor.*

**Supplementary Table S10. Paired bootstrap contrasts, nonlinear challengers, and the locked 2026 descriptive audit**

| **Contrast/model** | **Metric** | Observed difference / audit estimate | **95% CI low** | **95% CI high** | **Bootstrap n / audit n** | **Probability/interpretation** |
| --- | --- | --- | --- | --- | --- | --- |
| A. Observed M2 minus M1 differences with paired bootstrap intervals | | | | | | |
| M2 vs M1 | macro_AUPRC | 0.0321 | 0.0153 | 0.0572 | 500 | Pr(Δ > 0) = 1.000 |
| M2 vs M1 | macro_AUROC | 0.0179 | 0.0067 | 0.0314 | 500 | Pr(Δ > 0) = 0.998 |
| M2 vs M1 | multiclass_Brier | -0.0342 | -0.0406 | -0.0282 | 500 | Pr(Δ > 0) = 0.000 |
| **B. Nonlinear challengers versus locked M2** | | | | | | |
| XGBoost vs M2 | Macro-AUPRC | -0.0135 | -0.0381 | 0.0063 | 500 | Positive is better |
| XGBoost vs M2 | Macro-AUROC | -0.0003 | -0.0133 | 0.0140 | 500 | Positive is better |
| XGBoost vs M2 | Multiclass Brier | -0.0359 | -0.0491 | -0.0235 | 500 | Negative is better |
| XGBoost vs M2 | Log loss | -0.0626 | -0.0859 | -0.0417 | 500 | Negative is better |
| LightGBM vs M2 | Macro-AUPRC | -0.0048 | -0.0275 | 0.0195 | 500 | Positive is better |
| LightGBM vs M2 | Macro-AUROC | 0.0006 | -0.0150 | 0.0164 | 500 | Positive is better |
| LightGBM vs M2 | Multiclass Brier | -0.0435 | -0.0605 | -0.0274 | 500 | Negative is better |
| LightGBM vs M2 | Log loss | -0.0737 | -0.1043 | -0.0416 | 500 | Negative is better |
| **C. Locked 2026Q1–Q2 descriptive audit** | | | | | | |
| M1 multinomial elastic-net | macro-AUPRC / macro-AUROC | 0.656 / 0.850 | — | — | 139 | Descriptive only |
| M2 multinomial elastic-net + regimen | macro-AUPRC / macro-AUROC | 0.719 / 0.857 | — | — | 139 | Descriptive only |
| XGBoost challenger | macro-AUPRC / macro-AUROC | 0.670 / 0.848 | — | — | 139 | Descriptive only |
| LightGBM challenger | macro-AUPRC / macro-AUROC | 0.681 / 0.845 | — | — | 139 | Descriptive only |

Notes: For AUPRC and AUROC, positive differences favor the first model; for Brier score and log loss, negative differences favor the first model. The 2026 audit was descriptive because the immune class contained 14 reports. AUPRC, area under the precision–recall curve; AUROC, area under the receiver-operating-characteristic curve; CI, confidence interval; M1, phenotype-only classifier; M2, M1 plus treatment-regimen indicators. Section A reports observed differences; the bootstrap means were 0.0334, 0.0180 and −0.0343, respectively. Figure 4D displays those bootstrap means with percentile intervals. Pr(Δ > 0) is the bootstrap fraction, not a hypothesis-test P value.

**Supplementary Table S11. Prespecified mixed-case uncertainty and class probabilities**

| **Model** | **Group/time stratum** | **n** | **Maximum probability, median (IQR)** | **Normalized entropy, median (IQR)** | **Top-two gap, median** | **Class** | **Mean probability** |
| --- | --- | --- | --- | --- | --- | --- | --- |
| **A. Mixed-case uncertainty relative to temporal-exclusive cases** | | | | | | | |
| M1 multinomial elastic-net | mixed80 | 80 | 0.524 (0.493–0.576) | 0.882 (0.828–0.921) | 0.181 | — | — |
| M1 multinomial elastic-net | temporal_exclusive537 | 537 | 0.559 (0.473–0.687) | 0.852 (0.721–0.934) | 0.271 | — | — |
| M2 multinomial elastic-net + regimen | mixed80 | 80 | 0.542 (0.498–0.596) | 0.877 (0.803–0.919) | 0.239 | — | — |
| M2 multinomial elastic-net + regimen | temporal_exclusive537 | 537 | 0.589 (0.492–0.722) | 0.831 (0.682–0.928) | 0.322 | — | — |
| XGBoost challenger | mixed80 | 80 | 0.673 (0.565–0.775) | 0.730 (0.623–0.804) | 0.422 | — | — |
| XGBoost challenger | temporal_exclusive537 | 537 | 0.719 (0.574–0.880) | 0.677 (0.398–0.829) | 0.518 | — | — |
| LightGBM challenger | mixed80 | 80 | 0.692 (0.595–0.784) | 0.679 (0.595–0.749) | 0.486 | — | — |
| LightGBM challenger | temporal_exclusive537 | 537 | 0.767 (0.591–0.907) | 0.610 (0.332–0.796) | 0.595 | — | — |
| **B. Mean M2 class probabilities in mixed cases by time stratum** | | | | | | | |
| M2 multinomial elastic-net + regimen | Pre-2019 | 17 | — | — | — | Immune-IS | 0.243 |
| M2 multinomial elastic-net + regimen | Pre-2019 | 17 | — | — | — | VEGF-axis | 0.297 |
| M2 multinomial elastic-net + regimen | Pre-2019 | 17 | — | — | — | Nonspecific AKI | 0.460 |
| M2 multinomial elastic-net + regimen | Development | 39 | — | — | — | Immune-IS | 0.363 |
| M2 multinomial elastic-net + regimen | Development | 39 | — | — | — | VEGF-axis | 0.227 |
| M2 multinomial elastic-net + regimen | Development | 39 | — | — | — | Nonspecific AKI | 0.410 |
| M2 multinomial elastic-net + regimen | Validation | 16 | — | — | — | Immune-IS | 0.290 |
| M2 multinomial elastic-net + regimen | Validation | 16 | — | — | — | VEGF-axis | 0.435 |
| M2 multinomial elastic-net + regimen | Validation | 16 | — | — | — | Nonspecific AKI | 0.275 |
| M2 multinomial elastic-net + regimen | 2026 audit | 1 | — | — | — | Immune-IS | 0.574 |
| M2 multinomial elastic-net + regimen | 2026 audit | 1 | — | — | — | VEGF-axis | 0.196 |
| M2 multinomial elastic-net + regimen | 2026 audit | 1 | — | — | — | Nonspecific AKI | 0.230 |
| M2 multinomial elastic-net + regimen | Missing date | 7 | — | — | — | Immune-IS | 0.336 |
| M2 multinomial elastic-net + regimen | Missing date | 7 | — | — | — | VEGF-axis | 0.169 |
| M2 multinomial elastic-net + regimen | Missing date | 7 | — | — | — | Nonspecific AKI | 0.495 |

*Notes: The 80 mixed cases never entered training, tuning, or threshold selection. They were not required to receive a single correct label; the challenge evaluated uncertainty and coexistence of class probabilities. AKI, acute kidney injury; IQR, interquartile range; M1, phenotype-only classifier; M2, M1 plus treatment-regimen indicators; VEGF, vascular endothelial growth factor.*

**Supplementary Table S12. JADER 202607 release audit, regimen exclusivity, and phenotype-term coverage**

| **Check/regimen/English PT gloss** | **Value/phenotype** | **Status/exclusive PS n** | **Impact / phenotype concept** | **Excluded overlap/global n** | **PS-or-C/strict-RCC PS n** |
| --- | --- | --- | --- | --- | --- |
| **A. JADER data-quality audit** | | | | | |
| DEMO rows | 1043485 | INFO | source volume | — | — |
| DEMO unique primaryid | 1043485 | PASS | report grain confirmed | — | — |
| DEMO duplicated primaryid | 0 | PASS | no additional latest-version collapse possible | — | — |
| valid report quarter | 1043485 | PASS | year/quarter stratification valid | — | — |
| snapshot first quarter | 200401 | INFO | coverage | — | — |
| snapshot last quarter | 202601 | INFO | validation ends at 2026Q1 | — | — |
| 2026Q1 reports | 37 | INFO | 2026Q1 contains only 37 released reports and is interpreted as potentially truncated | — | — |
| DRUG rows | 5005056 | INFO | source volume | — | — |
| REAC rows | 1732544 | INFO | source volume | — | — |
| DRUG primaryid orphan vs DEMO | 0 | PASS | join integrity | — | — |
| REAC primaryid orphan vs DEMO | 0 | PASS | join integrity | — | — |
| target drug rows with canonical mapping | 117657 | INFO | exposure mapping | — | — |
| strict RCC PS base reports | 16572 | INFO | analytic denominator | — | — |
| PS role availability | 2079137 | PASS | suspected-drug main analysis feasible | — | — |
| raw source format | CP932 native JADER 202607 release | PASS | official native four-table release; indication is bound on the DRUG row | — | — |
| **B. Regimen exclusivity** | | | | | |
| Pembrolizumab + axitinib | 469 | 454 | 15 | 457 | — |
| Pembrolizumab + lenvatinib | 2,322 | 2,304 | 18 | 2,304 | — |
| Nivolumab + cabozantinib | 1,061 | 885 | 176 | 885 | — |
| Nivolumab + ipilimumab | 4,702 | 4,526 | 176 | 4,529 | — |
| Sunitinib | 2,436 | 2,436 | 0 | 2,444 | — |
| **C. Japanese PT coverage** | | | | | |
| Nephrotic Syndrome | VEGF-axis | Tier-A exact primary | NEPHROTIC SYNDROME | 2,282 | 118 |
| Immune Mediated Nephritis | Immune-IS | Tier-A exact primary | IMMUNE MEDIATED NEPHRITIS | — | 0 |
| Immune-Mediated Renal Disorder (Not Included in Tier-A) | Immune-IS | Broad semantic sensitivity only | IMMUNE-MEDIATED RENAL DISORDER (not included in Tier-A) | 265 | 43 |
| Tubulointerstitial/Interstitial Nephritis | Immune-IS | Tier-A exact primary | TUBULOINTERSTITIAL/INTERSTITIAL NEPHRITIS | 3,605 | 133 |
| Acute Kidney Injury / Renal Failure Acute | Nonspecific AKI | Tier-A exact primary | ACUTE KIDNEY INJURY / RENAL FAILURE ACUTE | 11,696 | 171 |
| Renal Limited Thrombotic Microangiopathy | VEGF-axis | Tier-A exact primary | RENAL LIMITED THROMBOTIC MICROANGIOPATHY | 19 | 0 |
| Autoimmune/Nephritis Autoimmune | Immune-IS | Tier-A exact primary | AUTOIMMUNE/NEPHRITIS AUTOIMMUNE | 27 | 5 |
| Proteinuria | VEGF-axis | Tier-A exact primary | PROTEINURIA | 2,283 | 344 |

*Notes: The 202607 release covered 2004Q1–2026Q1. The terminal 2026Q1 label contained 37 released reports and may be truncated.*

*Exact Japanese source PT strings are provided in data/table_source_data/S12_JADER_audit.csv in the Zenodo archive (https://doi.org/10.5281/zenodo.22807359). This table displays English glosses.*

*C, concomitant; CP932, Japanese code page 932; JADER, Japanese Adverse Drug Event Report database; PS, primary suspect; PT, preferred term; RCC, renal cell carcinoma.*

**Supplementary Table S13. JADER primary analysis and prespecified cohort/reporting-channel sensitivities**

| **Specification** | **Target regimen** | **Phenotype** | **Support start** | **Target events/reports** | **Comparator events/reports** | **MH-ROR (95% CI)** | **BH q** | **Evaluability/direction** |
| --- | --- | --- | --- | --- | --- | --- | --- | --- |
| **Primary PS exclusive** | | | | | | | | |
| Primary PS exclusive | Pembrolizumab + axitinib | Immune-IS | 2019Q4 | 7/439 | 54/3,659 | 1.25 (0.61–2.53) | 0.724 | Quantitatively evaluable |
| Primary PS exclusive | Pembrolizumab + axitinib | VEGF-axis | 2019Q4 | 5/439 | 6/3,659 | 6.28 (2.46–16.02) | 3.68e-04 | Quantitatively evaluable |
| Primary PS exclusive | Pembrolizumab + axitinib | Nonspecific AKI | 2019Q4 | 2/439 | 50/3,659 | 0.81 (0.32–2.03) | 0.730 | Sparse directional |
| Primary PS exclusive | Pembrolizumab + lenvatinib | Immune-IS | 2022Q1 | 14/2,304 | 21/1,634 | 0.60 (0.30–1.19) | 0.260 | Quantitatively evaluable |
| Primary PS exclusive | Pembrolizumab + lenvatinib | VEGF-axis | 2022Q1 | 184/2,304 | 6/1,634 | 19.27 (8.59–43.21) | 6.21e-12 | Quantitatively evaluable |
| Primary PS exclusive | Pembrolizumab + lenvatinib | Nonspecific AKI | 2022Q1 | 12/2,304 | 12/1,634 | 0.92 (0.41–2.10) | 0.849 | Quantitatively evaluable |
| Primary PS exclusive | Nivolumab + cabozantinib | Immune-IS | 2021Q3 | 1/873 | 27/2,096 | 0.29 (0.09–0.99) | 0.107 | Sparse directional |
| Primary PS exclusive | Nivolumab + cabozantinib | VEGF-axis | 2021Q3 | 19/873 | 6/2,096 | 6.16 (2.55–14.91) | 2.47e-04 | Quantitatively evaluable |
| Primary PS exclusive | Nivolumab + cabozantinib | Nonspecific AKI | 2021Q3 | 3/873 | 18/2,096 | 0.73 (0.24–2.15) | 0.724 | Quantitatively evaluable |
| **PS or concomitant** | | | | | | | | |
| Sensitivity PS or C exclusive | Pembrolizumab + axitinib | Immune-IS | 2019Q4 | 7/442 | 54/3,661 | 1.24 (0.61–2.52) | 0.723 | Quantitatively evaluable |
| Sensitivity PS or C exclusive | Pembrolizumab + axitinib | VEGF-axis | 2019Q4 | 5/442 | 6/3,661 | 6.24 (2.44–15.92) | 3.85e-04 | Quantitatively evaluable |
| Sensitivity PS or C exclusive | Pembrolizumab + axitinib | Nonspecific AKI | 2019Q4 | 2/442 | 50/3,661 | 0.80 (0.32–2.02) | 0.723 | Sparse directional |
| Sensitivity PS or C exclusive | Pembrolizumab + lenvatinib | Immune-IS | 2022Q1 | 14/2,304 | 21/1,634 | 0.60 (0.30–1.19) | 0.260 | Quantitatively evaluable |
| Sensitivity PS or C exclusive | Pembrolizumab + lenvatinib | VEGF-axis | 2022Q1 | 184/2,304 | 6/1,634 | 19.27 (8.59–43.21) | 6.21e-12 | Quantitatively evaluable |
| Sensitivity PS or C exclusive | Pembrolizumab + lenvatinib | Nonspecific AKI | 2022Q1 | 12/2,304 | 12/1,634 | 0.92 (0.41–2.10) | 0.849 | Quantitatively evaluable |
| Sensitivity PS or C exclusive | Nivolumab + cabozantinib | Immune-IS | 2021Q3 | 1/873 | 27/2,096 | 0.29 (0.09–0.99) | 0.107 | Sparse directional |
| Sensitivity PS or C exclusive | Nivolumab + cabozantinib | VEGF-axis | 2021Q3 | 19/873 | 6/2,096 | 6.16 (2.55–14.91) | 2.47e-04 | Quantitatively evaluable |
| Sensitivity PS or C exclusive | Nivolumab + cabozantinib | Nonspecific AKI | 2021Q3 | 3/873 | 18/2,096 | 0.73 (0.24–2.15) | 0.723 | Quantitatively evaluable |
| **Regimen overlap allowed** | | | | | | | | |
| Sensitivity PS overlap allowed | Pembrolizumab + axitinib | Immune-IS | 2019Q4 | 7/454 | 58/3,834 | 1.18 (0.58–2.38) | 0.767 | Quantitatively evaluable |
| Sensitivity PS overlap allowed | Pembrolizumab + axitinib | VEGF-axis | 2019Q4 | 5/454 | 10/3,834 | 4.61 (1.92–11.03) | 0.002 | Quantitatively evaluable |
| Sensitivity PS overlap allowed | Pembrolizumab + axitinib | Nonspecific AKI | 2019Q4 | 2/454 | 50/3,834 | 0.82 (0.33–2.07) | 0.767 | Sparse directional |
| Sensitivity PS overlap allowed | Pembrolizumab + lenvatinib | Immune-IS | 2022Q1 | 14/2,322 | 25/1,755 | 0.51 (0.26–0.98) | 0.097 | Quantitatively evaluable |
| Sensitivity PS overlap allowed | Pembrolizumab + lenvatinib | VEGF-axis | 2022Q1 | 186/2,322 | 10/1,755 | 13.11 (6.88–24.98) | 4.56e-14 | Quantitatively evaluable |
| Sensitivity PS overlap allowed | Pembrolizumab + lenvatinib | Nonspecific AKI | 2022Q1 | 12/2,322 | 12/1,755 | 0.98 (0.43–2.21) | 0.954 | Quantitatively evaluable |
| Sensitivity PS overlap allowed | Nivolumab + cabozantinib | Immune-IS | 2021Q3 | 5/1,007 | 31/2,230 | 0.46 (0.19–1.11) | 0.153 | Quantitatively evaluable |
| Sensitivity PS overlap allowed | Nivolumab + cabozantinib | VEGF-axis | 2021Q3 | 23/1,007 | 10/2,230 | 4.35 (2.10–8.99) | 3.37e-04 | Quantitatively evaluable |
| Sensitivity PS overlap allowed | Nivolumab + cabozantinib | Nonspecific AKI | 2021Q3 | 3/1,007 | 18/2,230 | 0.66 (0.22–1.96) | 0.688 | Quantitatively evaluable |
| **Expanded immune semantics** | | | | | | | | |
| Sensitivity broad immune semantic | Pembrolizumab + axitinib | Immune-IS | 2019Q4 | 10/439 | 68/3,659 | 1.26 (0.68–2.35) | 0.695 | Quantitatively evaluable |
| Sensitivity broad immune semantic | Pembrolizumab + axitinib | VEGF-axis | 2019Q4 | 5/439 | 6/3,659 | 6.28 (2.46–16.02) | 3.68e-04 | Quantitatively evaluable |
| Sensitivity broad immune semantic | Pembrolizumab + axitinib | Nonspecific AKI | 2019Q4 | 2/439 | 50/3,659 | 0.81 (0.32–2.03) | 0.730 | Sparse directional |
| Sensitivity broad immune semantic | Pembrolizumab + lenvatinib | Immune-IS | 2022Q1 | 27/2,304 | 33/1,634 | 0.67 (0.39–1.14) | 0.247 | Quantitatively evaluable |
| Sensitivity broad immune semantic | Pembrolizumab + lenvatinib | VEGF-axis | 2022Q1 | 183/2,304 | 6/1,634 | 19.14 (8.53–42.91) | 7.08e-12 | Quantitatively evaluable |
| Sensitivity broad immune semantic | Pembrolizumab + lenvatinib | Nonspecific AKI | 2022Q1 | 12/2,304 | 12/1,634 | 0.92 (0.41–2.10) | 0.849 | Quantitatively evaluable |
| Sensitivity broad immune semantic | Nivolumab + cabozantinib | Immune-IS | 2021Q3 | 1/873 | 40/2,096 | 0.18 (0.06–0.60) | 0.011 | Sparse directional |
| Sensitivity broad immune semantic | Nivolumab + cabozantinib | VEGF-axis | 2021Q3 | 19/873 | 6/2,096 | 6.16 (2.55–14.91) | 2.47e-04 | Quantitatively evaluable |
| Sensitivity broad immune semantic | Nivolumab + cabozantinib | Nonspecific AKI | 2021Q3 | 3/873 | 18/2,096 | 0.73 (0.24–2.15) | 0.724 | Quantitatively evaluable |
| **Reporting-channel sensitivity** | | | | | | | | |
| All reports | Nivolumab + cabozantinib | Nonspecific AKI | 2021Q3 | 3/873 | 18/2,096 | 0.73 (0.24–2.15) | 0.724 | <1 |
| Spontaneous only | Nivolumab + cabozantinib | Nonspecific AKI | 2021Q3 | 1/835 | 16/1,989 | 0.53 (0.15–1.90) | 0.425 | <1 |
| HCP only | Nivolumab + cabozantinib | Nonspecific AKI | 2021Q3 | 3/789 | 17/1,939 | 0.78 (0.26–2.34) | 0.743 | <1 |
| Spontaneous HCP only | Nivolumab + cabozantinib | Nonspecific AKI | 2021Q3 | 1/754 | 15/1,842 | 0.57 (0.16–2.06) | 0.505 | <1 |
| All reports | Nivolumab + cabozantinib | VEGF-axis | 2021Q3 | 19/873 | 6/2,096 | 6.16 (2.55–14.91) | 2.47e-04 | >1 |
| Spontaneous only | Nivolumab + cabozantinib | VEGF-axis | 2021Q3 | 17/835 | 6/1,989 | 5.51 (2.24–13.59) | 6.23e-04 | >1 |
| HCP only | Nivolumab + cabozantinib | VEGF-axis | 2021Q3 | 17/789 | 6/1,939 | 5.73 (2.34–14.04) | 6.11e-04 | >1 |
| Spontaneous HCP only | Nivolumab + cabozantinib | VEGF-axis | 2021Q3 | 15/754 | 6/1,842 | 5.06 (2.02–12.71) | 0.002 | >1 |
| All reports | Nivolumab + cabozantinib | Immune-IS | 2021Q3 | 1/873 | 27/2,096 | 0.29 (0.09–0.99) | 0.107 | <1 |
| Spontaneous only | Nivolumab + cabozantinib | Immune-IS | 2021Q3 | 1/835 | 27/1,989 | 0.29 (0.09–0.99) | 0.107 | <1 |
| HCP only | Nivolumab + cabozantinib | Immune-IS | 2021Q3 | 1/789 | 24/1,939 | 0.34 (0.10–1.17) | 0.196 | <1 |
| Spontaneous HCP only | Nivolumab + cabozantinib | Immune-IS | 2021Q3 | 1/754 | 24/1,842 | 0.34 (0.10–1.17) | 0.196 | <1 |
| All reports | Pembrolizumab + axitinib | Nonspecific AKI | 2019Q4 | 2/439 | 50/3,659 | 0.81 (0.32–2.03) | 0.730 | <1 |
| Spontaneous only | Pembrolizumab + axitinib | Nonspecific AKI | 2019Q4 | 0/347 | 43/3,249 | 0.75 (0.25–2.25) | 0.690 | <1 |
| HCP only | Pembrolizumab + axitinib | Nonspecific AKI | 2019Q4 | 1/354 | 45/3,257 | 0.78 (0.28–2.17) | 0.743 | <1 |
| Spontaneous HCP only | Pembrolizumab + axitinib | Nonspecific AKI | 2019Q4 | 0/275 | 40/2,924 | 0.90 (0.30–2.67) | 0.949 | <1 |
| All reports | Pembrolizumab + axitinib | VEGF-axis | 2019Q4 | 5/439 | 6/3,659 | 6.28 (2.46–16.02) | 3.68e-04 | >1 |
| Spontaneous only | Pembrolizumab + axitinib | VEGF-axis | 2019Q4 | 5/347 | 6/3,249 | 7.19 (2.81–18.40) | 1.76e-04 | >1 |
| HCP only | Pembrolizumab + axitinib | VEGF-axis | 2019Q4 | 4/354 | 6/3,257 | 6.24 (2.35–16.54) | 7.03e-04 | >1 |
| Spontaneous HCP only | Pembrolizumab + axitinib | VEGF-axis | 2019Q4 | 4/275 | 6/2,924 | 7.23 (2.72–19.26) | 3.38e-04 | >1 |
| All reports | Pembrolizumab + axitinib | Immune-IS | 2019Q4 | 7/439 | 54/3,659 | 1.25 (0.61–2.53) | 0.724 | >1 |
| Spontaneous only | Pembrolizumab + axitinib | Immune-IS | 2019Q4 | 7/347 | 46/3,249 | 1.71 (0.84–3.50) | 0.210 | >1 |
| HCP only | Pembrolizumab + axitinib | Immune-IS | 2019Q4 | 5/354 | 43/3,257 | 1.33 (0.60–2.93) | 0.717 | >1 |
| Spontaneous HCP only | Pembrolizumab + axitinib | Immune-IS | 2019Q4 | 5/275 | 40/2,924 | 1.74 (0.79–3.85) | 0.306 | >1 |
| All reports | Pembrolizumab + lenvatinib | Nonspecific AKI | 2022Q1 | 12/2,304 | 12/1,634 | 0.92 (0.41–2.10) | 0.849 | <1 |
| Spontaneous only | Pembrolizumab + lenvatinib | Nonspecific AKI | 2022Q1 | 12/2,274 | 10/1,569 | 1.03 (0.44–2.42) | 0.944 | >1 |
| HCP only | Pembrolizumab + lenvatinib | Nonspecific AKI | 2022Q1 | 10/1,884 | 12/1,535 | 0.92 (0.39–2.17) | 0.844 | <1 |
| Spontaneous HCP only | Pembrolizumab + lenvatinib | Nonspecific AKI | 2022Q1 | 10/1,862 | 10/1,478 | 1.02 (0.42–2.48) | 0.966 | >1 |
| All reports | Pembrolizumab + lenvatinib | VEGF-axis | 2022Q1 | 184/2,304 | 6/1,634 | 19.27 (8.59–43.21) | 6.21e-12 | >1 |
| Spontaneous only | Pembrolizumab + lenvatinib | VEGF-axis | 2022Q1 | 183/2,274 | 6/1,569 | 18.76 (8.37–42.01) | 9.32e-12 | >1 |
| HCP only | Pembrolizumab + lenvatinib | VEGF-axis | 2022Q1 | 136/1,884 | 6/1,535 | 16.72 (7.43–37.59) | 8.67e-11 | >1 |
| Spontaneous HCP only | Pembrolizumab + lenvatinib | VEGF-axis | 2022Q1 | 135/1,862 | 6/1,478 | 16.28 (7.26–36.55) | 1.20e-10 | >1 |
| All reports | Pembrolizumab + lenvatinib | Immune-IS | 2022Q1 | 14/2,304 | 21/1,634 | 0.60 (0.30–1.19) | 0.260 | <1 |
| Spontaneous only | Pembrolizumab + lenvatinib | Immune-IS | 2022Q1 | 14/2,274 | 21/1,569 | 0.58 (0.29–1.15) | 0.210 | <1 |
| HCP only | Pembrolizumab + lenvatinib | Immune-IS | 2022Q1 | 14/1,884 | 19/1,535 | 0.73 (0.36–1.51) | 0.717 | <1 |
| Spontaneous HCP only | Pembrolizumab + lenvatinib | Immune-IS | 2022Q1 | 14/1,862 | 19/1,478 | 0.71 (0.35–1.45) | 0.505 | <1 |

*All specifications reuse reports from the same JADER source and are not independent samples. The primary analysis was primary-suspect, regimen-exclusive, strict RCC. BH q values are displayed where available. AKI, acute kidney injury; BH, Benjamini–Hochberg; C, concomitant; CI, confidence interval; HCP, healthcare professional; JADER, Japanese Adverse Drug Event Report database; MH-ROR, Mantel–Haenszel reporting odds ratio; PS, primary suspect; RCC, renal cell carcinoma; VEGF, vascular endothelial growth factor.*

**Supplementary Table S14.** Cell-level FAERS–JADER concordance and geographic-source sensitivity

| **Target regimen** | **Phenotype** | **FAERS MH-ROR (95% CI)** | **FAERS BH q** | **JADER MH-ROR (95% CI)** | **JADER BH q** | **Same direction** | **Classification** |
| --- | --- | --- | --- | --- | --- | --- | --- |
| **A. Primary FAERS versus JADER** | | | | | | | |
| Nivolumab + cabozantinib | Nonspecific AKI | 0.78 (0.53–1.17) | 0.349 | 0.73 (0.24–2.15) | 0.724 | Yes | directionally concordant |
| Nivolumab + cabozantinib | VEGF-axis | 4.59 (2.07–10.19) | 3.13e-04 | 6.16 (2.55–14.91) | 2.47e-04 | Yes | directionally concordant |
| Nivolumab + cabozantinib | Immune-IS | 0.10 (0.05–0.22) | 3.27e-08 | 0.29 (0.09–0.99) | 0.107 | Yes | inconclusive due to sparsity |
| Pembrolizumab + axitinib | Nonspecific AKI | 1.05 (0.71–1.56) | 0.790 | 0.81 (0.32–2.03) | 0.730 | No | inconclusive due to sparsity |
| Pembrolizumab + axitinib | VEGF-axis | 5.31 (2.43–11.59) | 6.18e-05 | 6.28 (2.46–16.02) | 3.68e-04 | Yes | directionally concordant |
| Pembrolizumab + axitinib | Immune-IS | 0.92 (0.55–1.52) | 0.790 | 1.25 (0.61–2.53) | 0.724 | No | FAERS near null not directional |
| Pembrolizumab + lenvatinib | Nonspecific AKI | 0.93 (0.63–1.39) | 0.790 | 0.92 (0.41–2.10) | 0.849 | Yes | FAERS near null not directional |
| Pembrolizumab + lenvatinib | VEGF-axis | 19.48 (9.03–42.01) | 3.32e-13 | 19.27 (8.59–43.21) | 6.21e-12 | Yes | directionally concordant |
| Pembrolizumab + lenvatinib | Immune-IS | 0.29 (0.16–0.50) | 3.09e-05 | 0.60 (0.30–1.19) | 0.260 | Yes | directionally concordant |
| **B. FAERS after excluding any report linked to Japan versus JADER** | | | | | | | |
| Nivolumab + cabozantinib | Nonspecific AKI | 0.74 (0.48–1.12) | 0.282 | 0.73 (0.24–2.15) | 0.724 | Yes | Directional comparison |
| Nivolumab + cabozantinib | VEGF-axis | 1.63 (0.64–4.14) | 0.458 | 6.16 (2.55–14.91) | 2.47e-04 | Yes | Directional comparison |
| Nivolumab + cabozantinib | Immune-IS | 0.10 (0.04–0.23) | 7.65e-07 | 0.29 (0.09–0.99) | 0.107 | Yes | Directional comparison |
| Pembrolizumab + axitinib | Nonspecific AKI | 1.00 (0.66–1.51) | 0.999 | 0.81 (0.32–2.03) | 0.730 | No | Directional comparison |
| Pembrolizumab + axitinib | VEGF-axis | 2.37 (0.94–5.95) | 0.177 | 6.28 (2.46–16.02) | 3.68e-04 | Yes | Directional comparison |
| Pembrolizumab + axitinib | Immune-IS | 0.83 (0.47–1.49) | 0.609 | 1.25 (0.61–2.53) | 0.724 | No | Directional comparison |
| Pembrolizumab + lenvatinib | Nonspecific AKI | 1.16 (0.75–1.79) | 0.609 | 0.92 (0.41–2.10) | 0.849 | No | Directional comparison |
| Pembrolizumab + lenvatinib | VEGF-axis | 2.24 (0.91–5.52) | 0.177 | 19.27 (8.59–43.21) | 6.21e-12 | Yes | Directional comparison |
| Pembrolizumab + lenvatinib | Immune-IS | 0.16 (0.07–0.38) | 1.35e-04 | 0.60 (0.30–1.19) | 0.260 | Yes | Directional comparison |

*Notes: Near-null and sparse cells were not forced into a replication claim. Cross-database agreement does not prove report-level independence because record linkage across systems was unavailable. Excluding Japan-linked FAERS reports assesses geographic-source dependence but reduces precision. AKI, acute kidney injury; BH, Benjamini–Hochberg; CI, confidence interval; FAERS, FDA Adverse Event Reporting System; JADER, Japanese Adverse Drug Event Report database; MH-ROR, Mantel–Haenszel reporting odds ratio; VEGF, vascular endothelial growth factor.*

**Supplementary Table S15. Leave-one-year-out stability, sender concentration, and prespecified robustness judgments**

| **Regimen/check** | **Phenotype/observed** | **Baseline/HHI/estimate/status** | **Point-estimate range/target retained, %** | **CI envelope/comparator retained, %** | **Direction/top sender/evaluability** | **Omissions/total reports** | **Event reports** |
| --- | --- | --- | --- | --- | --- | --- | --- |
| **A. Leave-one-year-out envelopes** | | | | | | | |
| Pembrolizumab + axitinib | Immune-IS | 0.916 | 0.791–1.096 | 0.462–1.915 | No | 8 | — |
| Pembrolizumab + axitinib | VEGF-axis | 5.376 | 4.714–6.547 | 1.896–15.452 | Yes | 8 | — |
| Pembrolizumab + axitinib | Nonspecific AKI | 1.064 | 0.909–1.322 | 0.575–2.023 | No | 8 | — |
| Pembrolizumab + lenvatinib | Immune-IS | 0.287 | 0.259–0.341 | 0.136–0.666 | Yes | 6 | — |
| Pembrolizumab + lenvatinib | VEGF-axis | 19.483 | 16.228–22.571 | 7.033–56.628 | Yes | 6 | — |
| Pembrolizumab + lenvatinib | Nonspecific AKI | 0.935 | 0.806–1.026 | 0.530–1.707 | No | 6 | — |
| Nivolumab + cabozantinib | Immune-IS | 0.102 | 0.059–0.157 | 0.021–0.369 | Yes | 6 | — |
| Nivolumab + cabozantinib | VEGF-axis | 4.599 | 4.123–5.064 | 1.730–12.733 | Yes | 6 | — |
| Nivolumab + cabozantinib | Nonspecific AKI | 0.789 | 0.690–0.897 | 0.445–1.389 | Yes | 6 | — |
| **B. Sender concentration** | | | | | | | |
| Pembrolizumab + axitinib | Immune-IS | 0.448 | 51.5 | 36.4 | MERCK | 1,557 | 22 |
| Pembrolizumab + axitinib | VEGF-axis | 0.448 | 51.5 | 68.2 | MERCK | 1,557 | 22 |
| Pembrolizumab + axitinib | Nonspecific AKI | 0.448 | 51.5 | 45.9 | MERCK | 1,557 | 37 |
| Nivolumab + ipilimumab | Immune-IS | 0.914 | 95.6 | 80.8 | BRISTOL MYERS SQUIBB | 3,988 | 52 |
| Nivolumab + ipilimumab | VEGF-axis | 0.914 | 95.6 | 100.0 | BRISTOL MYERS SQUIBB | 3,988 | 7 |
| Nivolumab + ipilimumab | Nonspecific AKI | 0.914 | 95.6 | 92.6 | BRISTOL MYERS SQUIBB | 3,988 | 94 |
| Pembrolizumab + lenvatinib | Immune-IS | 0.496 | 55.0 | 68.2 | EISAI | 4,471 | 22 |
| Pembrolizumab + lenvatinib | VEGF-axis | 0.496 | 55.0 | 29.7 | EISAI | 4,471 | 330 |
| Pembrolizumab + lenvatinib | Nonspecific AKI | 0.496 | 55.0 | 69.0 | EISAI | 4,471 | 84 |
| Nivolumab + ipilimumab | Immune-IS | 0.885 | 94.0 | 70.6 | BRISTOL MYERS SQUIBB | 1,997 | 34 |
| Nivolumab + ipilimumab | VEGF-axis | 0.885 | 94.0 | 100.0 | BRISTOL MYERS SQUIBB | 1,997 | 6 |
| Nivolumab + ipilimumab | Nonspecific AKI | 0.885 | 94.0 | 87.0 | BRISTOL MYERS SQUIBB | 1,997 | 46 |
| Nivolumab + cabozantinib | Immune-IS | 0.479 | 52.9 | 44.4 | EXELIXIS | 4,914 | 9 |
| Nivolumab + cabozantinib | VEGF-axis | 0.479 | 52.9 | 52.3 | EXELIXIS | 4,914 | 88 |
| Nivolumab + cabozantinib | Nonspecific AKI | 0.479 | 52.9 | 39.7 | EXELIXIS | 4,914 | 68 |
| Nivolumab + ipilimumab | Immune-IS | 0.893 | 94.5 | 73.0 | BRISTOL MYERS SQUIBB | 2,317 | 37 |
| Nivolumab + ipilimumab | VEGF-axis | 0.893 | 94.5 | 100.0 | BRISTOL MYERS SQUIBB | 2,317 | 6 |
| Nivolumab + ipilimumab | Nonspecific AKI | 0.893 | 94.5 | 87.0 | BRISTOL MYERS SQUIBB | 2,317 | 54 |
| **C. Regimen-specific top-sender exclusion** | | | | | | | |
| Nivolumab + cabozantinib | Nonspecific AKI | 0.27 (0.12–0.59) | 47.1 | 5.5 | NON EVALUABLE POSITIVITY OR ZERO EVENT | — | — |
| Nivolumab + cabozantinib | VEGF-axis | 0.74 (0.22–2.44) | 47.1 | 5.5 | NON EVALUABLE POSITIVITY OR ZERO EVENT | — | — |
| Nivolumab + cabozantinib | Immune-IS | 0.03 (0.01–0.07) | 47.1 | 5.5 | NON EVALUABLE POSITIVITY OR ZERO EVENT | — | — |
| Pembrolizumab + axitinib | Nonspecific AKI | 0.60 (0.27–1.35) | 48.5 | 4.4 | NON EVALUABLE POSITIVITY OR ZERO EVENT | — | — |
| Pembrolizumab + axitinib | VEGF-axis | 0.62 (0.19–1.99) | 48.5 | 4.4 | NON EVALUABLE POSITIVITY OR ZERO EVENT | — | — |
| Pembrolizumab + axitinib | Immune-IS | 0.29 (0.13–0.65) | 48.5 | 4.4 | NON EVALUABLE POSITIVITY OR ZERO EVENT | — | — |
| Pembrolizumab + lenvatinib | Nonspecific AKI | 0.21 (0.09–0.52) | 45.0 | 6.0 | NON EVALUABLE POSITIVITY OR ZERO EVENT | — | — |
| Pembrolizumab + lenvatinib | VEGF-axis | 4.69 (1.46–15.09) | 45.0 | 6.0 | NON EVALUABLE POSITIVITY OR ZERO EVENT | — | — |
| Pembrolizumab + lenvatinib | Immune-IS | 0.06 (0.02–0.15) | 45.0 | 6.0 | NON EVALUABLE POSITIVITY OR ZERO EVENT | — | — |
| **D. Prespecified robustness judgments** | | | | | | | |
| FAERS exclude-any-Japan: substantive (non-near-null) directions retained | 6/6 | PASS | — | — | — | — | — |
| FAERS exclude-any-Japan: VEGF point estimates remain >1 | 3/3 | PASS | — | — | — | — | — |
| FAERS exclude-any-Japan: VEGF lower 95% CI remains >1 | 0/3 | LIMITATION | — | — | — | — | — |
| FAERS leave-one-year-out: every VEGF estimate remains >1 with lower CI >1 | 20/20 | PASS | — | — | — | — | — |
| JADER spontaneous/HCP restrictions: every VEGF estimate remains >1 with lower CI >1 | 12/12 | PASS | — | — | — | — | — |
| FAERS delete proteinuria family: VEGF direction / lower CI >1 | 3/3 direction; 0/3 lower CI | LIMITATION | — | — | — | — | — |
| JADER delete proteinuria family: VEGF direction / lower CI >1 | 3/3 direction; 1/3 lower CI | LIMITATION | — | — | — | — | — |
| FAERS nivo+ipi top-sender share across support windows | 94.0%–95.6% | LIMITATION | — | — | — | — | — |
| FAERS top-sender exclusion quantitatively evaluable for VEGF | 0/3 | LIMITATION | — | — | — | — | — |

*Notes: HHI is the Herfindahl–Hirschman index. All nine top-sender exclusion cells were non-evaluable because positivity or zero-event requirements failed; finite post-exclusion estimates must not be interpreted quantitatively. AKI, acute kidney injury; CI, confidence interval; FAERS, FDA Adverse Event Reporting System; HCP, healthcare professional; JADER, Japanese Adverse Drug Event Report database; VEGF, vascular endothelial growth factor.*

# Supplementary Table S16. Annual cells, sparse-stratum handling and weights

All nine baseline MH-RORs reproduce the primary results to numerical precision. Table S5 provides raw annual 2×2 counts; this table supplies diagnostics and correction sensitivity. Denominator weights are proportional to corrected b×c/n and sum to 100% within a cell. Q and I² summarize corrected stratum log-odds heterogeneity descriptively; small event counts make absence of detected heterogeneity uninformative.

| **Target** | **Proxy** | **Events target** | **Events N+Ipi** | **Zero/year strata** | **Q** | **I² %** |
| --- | --- | --- | --- | --- | --- | --- |
| P+Axi | Immune-IS | 22 | 50 | 1/8 | 6.49 | 0.0 |
| P+Axi | VEGF-axis | 22 | 7 | 5/8 | 4.32 | 0.0 |
| P+Axi | AKI | 37 | 91 | 0/8 | 15.68 | 55.4 |
| P+Lenva | Immune-IS | 22 | 34 | 1/6 | 2.31 | 0.0 |
| P+Lenva | VEGF-axis | 330 | 6 | 2/6 | 2.20 | 0.0 |
| P+Lenva | AKI | 84 | 46 | 0/6 | 6.31 | 20.7 |
| N+Cabo | Immune-IS | 9 | 37 | 3/6 | 9.32 | 46.3 |
| N+Cabo | VEGF-axis | 88 | 6 | 2/6 | 1.15 | 0.0 |
| N+Cabo | AKI | 68 | 54 | 0/6 | 5.51 | 9.2 |

| **Target** | **Proxy** | **Sparse-cell specification** | **MH-ROR (95% CI)** |
| --- | --- | --- | --- |
| P+Axi | Immune-IS | All cells +0.5 if zero | 0.92 (0.55–1.52) |
| P+Axi | Immune-IS | All cells +0.1 if zero | 0.89 (0.54–1.49) |
| P+Axi | Immune-IS | Zero cells only +0.5 | 0.92 (0.55–1.53) |
| P+Axi | Immune-IS | Uncorrected MH | 0.89 (0.53–1.49) |
| P+Axi | Immune-IS | Omit zero strata | 0.91 (0.54–1.53) |
| P+Axi | VEGF-axis | All cells +0.5 if zero | 5.31 (2.43–11.59) |
| P+Axi | VEGF-axis | All cells +0.1 if zero | 5.90 (2.50–13.92) |
| P+Axi | VEGF-axis | Zero cells only +0.5 | 5.38 (2.39–12.12) |
| P+Axi | VEGF-axis | Uncorrected MH | 6.10 (2.52–14.72) |
| P+Axi | VEGF-axis | Omit zero strata | 4.65 (1.65–13.07) |
| P+Axi | AKI | All cells +0.5 if zero | 1.05 (0.71–1.56) |
| P+Axi | AKI | All cells +0.1 if zero | 1.05 (0.71–1.56) |
| P+Axi | AKI | Zero cells only +0.5 | 1.05 (0.71–1.56) |
| P+Axi | AKI | Uncorrected MH | 1.05 (0.71–1.56) |
| P+Axi | AKI | Omit zero strata | 1.05 (0.71–1.56) |
| P+Lenva | Immune-IS | All cells +0.5 if zero | 0.29 (0.16–0.50) |
| P+Lenva | Immune-IS | All cells +0.1 if zero | 0.27 (0.16–0.48) |
| P+Lenva | Immune-IS | Zero cells only +0.5 | 0.29 (0.17–0.50) |
| P+Lenva | Immune-IS | Uncorrected MH | 0.27 (0.15–0.47) |
| P+Lenva | Immune-IS | Omit zero strata | 0.28 (0.16–0.49) |
| P+Lenva | VEGF-axis | All cells +0.5 if zero | 19.48 (9.03–42.01) |
| P+Lenva | VEGF-axis | All cells +0.1 if zero | 21.11 (9.45–47.17) |
| P+Lenva | VEGF-axis | Zero cells only +0.5 | 19.45 (9.02–41.94) |
| P+Lenva | VEGF-axis | Uncorrected MH | 21.57 (9.56–48.66) |
| P+Lenva | VEGF-axis | Omit zero strata | 17.50 (7.75–39.52) |
| P+Lenva | AKI | All cells +0.5 if zero | 0.93 (0.63–1.39) |
| P+Lenva | AKI | All cells +0.1 if zero | 0.93 (0.63–1.39) |
| P+Lenva | AKI | Zero cells only +0.5 | 0.93 (0.63–1.39) |
| P+Lenva | AKI | Uncorrected MH | 0.93 (0.63–1.39) |
| P+Lenva | AKI | Omit zero strata | 0.93 (0.63–1.39) |
| N+Cabo | Immune-IS | All cells +0.5 if zero | 0.10 (0.05–0.22) |
| N+Cabo | Immune-IS | All cells +0.1 if zero | 0.08 (0.04–0.18) |
| N+Cabo | Immune-IS | Zero cells only +0.5 | 0.11 (0.05–0.23) |
| N+Cabo | Immune-IS | Uncorrected MH | 0.08 (0.03–0.17) |
| N+Cabo | Immune-IS | Omit zero strata | 0.22 (0.09–0.57) |
| N+Cabo | VEGF-axis | All cells +0.5 if zero | 4.59 (2.07–10.19) |
| N+Cabo | VEGF-axis | All cells +0.1 if zero | 4.95 (2.15–11.41) |
| N+Cabo | VEGF-axis | Zero cells only +0.5 | 4.49 (2.02–9.98) |
| N+Cabo | VEGF-axis | Uncorrected MH | 5.05 (2.17–11.76) |
| N+Cabo | VEGF-axis | Omit zero strata | 4.02 (1.71–9.45) |
| N+Cabo | AKI | All cells +0.5 if zero | 0.78 (0.53–1.17) |
| N+Cabo | AKI | All cells +0.1 if zero | 0.78 (0.53–1.17) |
| N+Cabo | AKI | Zero cells only +0.5 | 0.78 (0.53–1.17) |
| N+Cabo | AKI | Uncorrected MH | 0.78 (0.53–1.17) |
| N+Cabo | AKI | Omit zero strata | 0.78 (0.53–1.17) |

| **Target** | **Proxy** | **Year** | **Target event/non-event** | **N+Ipi event/non-event** | **Zero** | **MH weight %** |
| --- | --- | --- | --- | --- | --- | --- |
| P+Axi | Immune-IS | 2019 | 0/56 | 5/680 | Yes | 2.6 |
| P+Axi | Immune-IS | 2020 | 2/213 | 8/787 | No | 10.7 |
| P+Axi | Immune-IS | 2021 | 2/289 | 7/639 | No | 13.7 |
| P+Axi | Immune-IS | 2022 | 5/335 | 9/531 | No | 21.7 |
| P+Axi | Immune-IS | 2023 | 4/263 | 7/365 | No | 18.3 |
| P+Axi | Immune-IS | 2024 | 3/202 | 11/311 | No | 26.7 |
| P+Axi | Immune-IS | 2025 | 3/109 | 1/306 | No | 1.6 |
| P+Axi | Immune-IS | 2026 | 3/67 | 2/110 | No | 4.7 |
| P+Axi | VEGF-axis | 2019 | 0/56 | 1/684 | Yes | 3.8 |
| P+Axi | VEGF-axis | 2020 | 2/213 | 0/795 | Yes | 3.5 |
| P+Axi | VEGF-axis | 2021 | 3/288 | 0/646 | Yes | 5.2 |
| P+Axi | VEGF-axis | 2022 | 9/331 | 2/538 | No | 25.2 |
| P+Axi | VEGF-axis | 2023 | 4/263 | 2/370 | No | 27.6 |
| P+Axi | VEGF-axis | 2024 | 3/202 | 0/322 | Yes | 6.4 |
| P+Axi | VEGF-axis | 2025 | 1/111 | 1/306 | No | 8.9 |
| P+Axi | VEGF-axis | 2026 | 0/70 | 1/111 | Yes | 19.3 |
| P+Axi | AKI | 2019 | 1/55 | 16/669 | No | 4.9 |
| P+Axi | AKI | 2020 | 10/205 | 21/774 | No | 17.7 |
| P+Axi | AKI | 2021 | 7/284 | 21/625 | No | 26.5 |
| P+Axi | AKI | 2022 | 4/336 | 17/523 | No | 27.0 |
| P+Axi | AKI | 2023 | 2/265 | 4/368 | No | 6.9 |
| P+Axi | AKI | 2024 | 2/203 | 7/315 | No | 11.2 |
| P+Axi | AKI | 2025 | 6/106 | 4/303 | No | 4.2 |
| P+Axi | AKI | 2026 | 5/65 | 1/111 | No | 1.5 |
| P+Lenva | Immune-IS | 2021 | 0/97 | 4/335 | Yes | 4.5 |
| P+Lenva | Immune-IS | 2022 | 5/784 | 9/531 | No | 24.0 |
| P+Lenva | Immune-IS | 2023 | 6/1383 | 7/365 | No | 24.8 |
| P+Lenva | Immune-IS | 2024 | 6/886 | 11/311 | No | 36.2 |
| P+Lenva | Immune-IS | 2025 | 3/883 | 1/306 | No | 3.3 |
| P+Lenva | Immune-IS | 2026 | 2/416 | 2/110 | No | 7.1 |
| P+Lenva | VEGF-axis | 2021 | 0/97 | 0/339 | Yes | 2.5 |
| P+Lenva | VEGF-axis | 2022 | 44/745 | 2/538 | No | 25.3 |
| P+Lenva | VEGF-axis | 2023 | 93/1296 | 2/370 | No | 33.2 |
| P+Lenva | VEGF-axis | 2024 | 61/831 | 0/322 | Yes | 7.7 |
| P+Lenva | VEGF-axis | 2025 | 98/788 | 1/306 | No | 14.9 |
| P+Lenva | VEGF-axis | 2026 | 34/384 | 1/111 | No | 16.4 |
| P+Lenva | AKI | 2021 | 1/96 | 13/326 | No | 11.7 |
| P+Lenva | AKI | 2022 | 20/769 | 17/523 | No | 40.1 |
| P+Lenva | AKI | 2023 | 22/1367 | 4/368 | No | 12.6 |
| P+Lenva | AKI | 2024 | 18/874 | 7/315 | No | 20.5 |
| P+Lenva | AKI | 2025 | 5/881 | 4/303 | No | 12.0 |
| P+Lenva | AKI | 2026 | 18/400 | 1/111 | No | 3.1 |
| N+Cabo | Immune-IS | 2021 | 0/239 | 7/639 | Yes | 8.5 |
| N+Cabo | Immune-IS | 2022 | 0/629 | 9/531 | Yes | 21.4 |
| N+Cabo | Immune-IS | 2023 | 1/1123 | 7/365 | No | 22.1 |
| N+Cabo | Immune-IS | 2024 | 0/1130 | 11/311 | Yes | 37.5 |
| N+Cabo | Immune-IS | 2025 | 5/1111 | 1/306 | No | 3.3 |
| N+Cabo | Immune-IS | 2026 | 3/673 | 2/110 | No | 7.2 |
| N+Cabo | VEGF-axis | 2021 | 1/238 | 0/646 | Yes | 2.9 |
| N+Cabo | VEGF-axis | 2022 | 7/622 | 2/538 | No | 22.8 |
| N+Cabo | VEGF-axis | 2023 | 24/1100 | 2/370 | No | 31.6 |
| N+Cabo | VEGF-axis | 2024 | 16/1114 | 0/322 | Yes | 8.2 |
| N+Cabo | VEGF-axis | 2025 | 25/1091 | 1/306 | No | 16.5 |
| N+Cabo | VEGF-axis | 2026 | 15/661 | 1/111 | No | 18.0 |
| N+Cabo | AKI | 2021 | 6/233 | 21/625 | No | 20.6 |
| N+Cabo | AKI | 2022 | 13/616 | 17/523 | No | 33.4 |
| N+Cabo | AKI | 2023 | 19/1105 | 4/368 | No | 11.0 |
| N+Cabo | AKI | 2024 | 9/1121 | 7/315 | No | 20.2 |
| N+Cabo | AKI | 2025 | 19/1097 | 4/303 | No | 11.5 |
| N+Cabo | AKI | 2026 | 2/674 | 1/111 | No | 3.2 |

Raw counts are never replaced by continuity-corrected pseudo-counts in these displays. Alternative specifications are sensitivity analyses, not model selection or multiple independent confirmations.

# Supplementary Table S17. Exclusion of all 2026 report-year strata

| **FAERS target** | **Proxy** | **MH-ROR (95% CI)** |
| --- | --- | --- |
| P+Axi | Immune-IS | 0.84 (0.49–1.44) |
| P+Axi | VEGF-axis | 6.45 (2.77–15.02) |
| P+Axi | AKI | 0.94 (0.62–1.42) |
| P+Lenva | Immune-IS | 0.29 (0.16–0.51) |
| P+Lenva | VEGF-axis | 21.36 (9.28–49.20) |
| P+Lenva | AKI | 0.80 (0.53–1.22) |
| N+Cabo | Immune-IS | 0.09 (0.04–0.22) |
| N+Cabo | VEGF-axis | 5.05 (2.13–12.00) |
| N+Cabo | AKI | 0.80 (0.53–1.20) |

| **Target** | **Query** | **Target events/reports** | **N+Ipi events/reports** | **MH-ROR (95% CI)** | **Zero strata** |
| --- | --- | --- | --- | --- | --- |
| P+Axi | Immune-IS | 7/439 | 54/3659 | 1.25 (0.61–2.53) | 5/7 |
| P+Axi | VEGF-axis | 5/439 | 6/3659 | 6.28 (2.46–16.02) | 6/7 |
| P+Axi | AKI | 2/439 | 50/3659 | Sparse; not interpreted | 6/7 |
| P+Lenva | Immune-IS | 14/2304 | 21/1634 | 0.60 (0.30–1.19) | 1/4 |
| P+Lenva | VEGF-axis | 184/2304 | 6/1634 | 19.27 (8.59–43.21) | 1/4 |
| P+Lenva | AKI | 12/2304 | 12/1634 | 0.92 (0.41–2.10) | 1/4 |
| N+Cabo | Immune-IS | 1/873 | 27/2096 | Sparse; not interpreted | 4/5 |
| N+Cabo | VEGF-axis | 19/873 | 6/2096 | 6.16 (2.55–14.91) | 2/5 |
| N+Cabo | AKI | 3/873 | 18/2096 | 0.73 (0.24–2.15) | 3/5 |

JADER estimates are unchanged after excluding 2026 strata. Sparse immune/AKI cells remain non-quantitative under the original ≥3 events-per-arm rule. Exclusion does not prove release completeness, resolve reporting lag, or reconstruct an earlier data vintage.

# Supplementary Table S18. Standard narrow renal queries and component analyses

Basis: separate ancillary FAERS reconstruction; 15,155/15,155 reports have joined reaction records after raw-file supplementation of 371 initially unmatched reports. Cohort and query checks are supplied in the reproducibility archive. These are not numerically identical to the primary cohort; denominators and a primary-reconstruction comparison are supplied. Standard-query and component events are not forced into mutually exclusive classes.

| **Target** | **Query** | **Target events/reports** | **N+Ipi events/reports** | **MH-ROR (95% CI)** | **Zero strata** |
| --- | --- | --- | --- | --- | --- |
| P+Axi | acute renal failure SMQ narrow | 87/1557 | 216/3988 | 1.09 (0.83–1.42) | 0/8 |
| P+Axi | proteinuria SMQ narrow | 27/1557 | 5/3988 | 8.88 (3.80–20.78) | 5/8 |
| P+Axi | tubulointerstitial diseases SMQ narrow | 28/1557 | 47/3988 | 1.25 (0.78–2.02) | 2/8 |
| P+Lenva | acute renal failure SMQ narrow | 291/4471 | 96/1997 | 1.56 (1.20–2.02) | 0/6 |
| P+Lenva | proteinuria SMQ narrow | 337/4471 | 4/1997 | 25.27 (10.64–59.97) | 3/6 |
| P+Lenva | tubulointerstitial diseases SMQ narrow | 18/4471 | 31/1997 | 0.26 (0.14–0.48) | 1/6 |
| N+Cabo | acute renal failure SMQ narrow | 138/4914 | 114/2317 | 0.73 (0.55–0.97) | 0/6 |
| N+Cabo | proteinuria SMQ narrow | 91/4914 | 4/2317 | 5.78 (2.37–14.07) | 3/6 |
| N+Cabo | tubulointerstitial diseases SMQ narrow | 4/4914 | 35/2317 | 0.07 (0.03–0.20) | 3/6 |

Narrow retrieval uses active MedDRA 29.0 PT-level members (term level 4, scope 2, status A) of acute renal failure (20000003), proteinuria (20000220) and tubulointerstitial diseases (20000221). It uses exact current PT names and does not establish complete historical remapping. The acute-renal-failure result for P+Lenva, 1.56 (1.20–2.02), qualifies the original exclusive-AKI interpretation. Query multiplicity values are exploratory and not part of the primary nine-cell family.

| **Target** | **Query** | **Target events/reports** | **N+Ipi events/reports** | **MH-ROR (95% CI)** | **Zero strata** |
| --- | --- | --- | --- | --- | --- |
| P+Axi | proteinuria | 22/1557 | 4/3988 | 9.02 (3.54–22.98) | 5/8 |
| P+Axi | nephrotic syndrome | 1/1557 | 6/3988 | Sparse; not interpreted | 7/8 |
| P+Axi | renal limited TMA | 1/1557 | 0/3988 | Sparse; not interpreted | 8/8 |
| P+Lenva | proteinuria | 321/4471 | 3/1997 | 30.41 (11.59–79.78) | 3/6 |
| P+Lenva | nephrotic syndrome | 21/4471 | 6/1997 | 1.09 (0.47–2.52) | 3/6 |
| P+Lenva | renal limited TMA | 0/4471 | 0/1997 | Sparse; not interpreted | 6/6 |
| N+Cabo | proteinuria | 67/4914 | 3/2317 | 5.42 (2.01–14.62) | 3/6 |
| N+Cabo | nephrotic syndrome | 21/4914 | 6/2317 | 1.19 (0.50–2.83) | 2/6 |
| N+Cabo | renal limited TMA | 1/4914 | 0/2317 | Sparse; not interpreted | 6/6 |

Individual component queries may include mixed reports; their counts are therefore not exclusive Tier-A counts. Renal-limited TMA counts are 1 versus 0, 0 versus 0, and 1 versus 0: not quantitatively interpretable. No estimate is assigned to the double-zero comparison.

| **Target** | **Query** | **Target events/reports** | **N+Ipi events/reports** | **MH-ROR (95% CI)** | **Zero strata** |
| --- | --- | --- | --- | --- | --- |
| P+Axi | hypertension related | 170/1557 | 39/3988 | 11.51 (7.93–16.71) | 0/8 |
| P+Axi | haematuria | 9/1557 | 20/3988 | 1.65 (0.79–3.48) | 6/8 |
| P+Axi | VEGF with hypertension or haematuria | 5/1557 | 0/3988 | Sparse; not interpreted | 8/8 |
| P+Lenva | hypertension related | 773/4471 | 23/1997 | 18.58 (11.98–28.80) | 0/6 |
| P+Lenva | haematuria | 27/4471 | 8/1997 | 1.80 (0.78–4.16) | 3/6 |
| P+Lenva | VEGF with hypertension or haematuria | 155/4471 | 0/1997 | Sparse; not interpreted | 6/6 |
| N+Cabo | hypertension related | 793/4914 | 25/2317 | 20.00 (13.10–30.53) | 0/6 |
| N+Cabo | haematuria | 25/4914 | 9/2317 | 1.56 (0.69–3.56) | 3/6 |
| N+Cabo | VEGF with hypertension or haematuria | 25/4914 | 0/2317 | Sparse; not interpreted | 6/6 |

The co-occurrence query has no comparator events and is not interpreted quantitatively. Hypertension/haematuria queries add context without adjudicating endothelial pathology. Exact term lists and retrieval membership are included in machine-readable manifests.

| **Concept** | **Rationale and limitation** | **Normalization / disposition** |
| --- | --- | --- |
| Tubulointerstitial / interstitial nephritis | Lexically interstitial; not uniquely immune | NEPHRITIS INTERSTITIAL retained as explicit historical expression |
| Immune-mediated / autoimmune nephritis | Explicit immune wording; not adjudicated | NEPHRITIS AUTOIMMUNE retained alongside AUTOIMMUNE NEPHRITIS |
| Proteinuria | Observable proteinuric manifestation; several causes | Exact PROTEINURIA anchor; no inference from a positive urine test alone |
| Nephrotic syndrome | Proteinuric syndrome; heterogeneous pathology | Exact term; neither biopsy nor VEGF causality assumed |
| Renal-limited TMA | Renal-qualified textual TMA concept | Exact full expression only; generic TMA is excluded from primary family |
| AKI / renal failure acute | Nonspecific acute renal dysfunction | RENAL FAILURE ACUTE retained as explicit historical expression |
| Creatinine increase, generic nephritis, chronic renal disease | Insufficient specificity for primary family | Not silently substituted for anchors; relevant standard queries are separate |
| Generic TMA, HUS, broader immune renal disorder | Systemic/semantic breadth differs | Excluded from primary exact family; broader analyses labelled separately |

Normalization is case/punctuation/whitespace harmonization followed by complete-string matching, not a claim that every historical source string is a current PT. No alias can move a report between frozen families. MedDRA distribution-format guidance: https://admin.meddra.org/sites/default/files/guidance/file/dist_file_format_24_0_English.pdf (field layout checked against the 29.0 local files).

# Supplementary Table S19. Nonrenal co-reporting and reporting-source context

| **Regimen** | **Query** | **AKI reports** | **With query** | **Share %** |
| --- | --- | --- | --- | --- |
| P+Lenva | Explicit organ immune terms | 91 | 6 | 6.6 |
| N+Ipi | Explicit organ immune terms | 94 | 21 | 22.3 |
| P+Axi | Explicit organ immune terms | 37 | 0 | 0.0 |
| N+Cabo | Explicit organ immune terms | 70 | 6 | 8.6 |
| P+Lenva | Extended irAE-compatible terms | 91 | 22 | 24.2 |
| N+Ipi | Extended irAE-compatible terms | 94 | 39 | 41.5 |
| P+Axi | Extended irAE-compatible terms | 37 | 2 | 5.4 |
| N+Cabo | Extended irAE-compatible terms | 70 | 15 | 21.4 |

Descriptive basis: reconstructed four-regimen reports with initial date from 2019Q2, before applying each target’s later support start; these are not pairwise incidence comparisons. Extended terms include nonspecific organ conditions and are not validated irAEs. No report was reclassified. The exact core/extended lists are included in Ancillary_PT_query_manifest.csv.

| **Regimen** | **Year** | **Reports** | **Immune-IS** | **VEGF-axis** | **AKI** | **Proteinuria** |
| --- | --- | --- | --- | --- | --- | --- |
| N+Cabo | 2019 | 76 | 0 | 0 | 1 | 0 |
| N+Cabo | 2020 | 80 | 0 | 2 | 1 | 2 |
| N+Cabo | 2021 | 239 | 0 | 1 | 6 | 1 |
| N+Cabo | 2022 | 629 | 0 | 7 | 13 | 4 |
| N+Cabo | 2023 | 1124 | 1 | 24 | 19 | 17 |
| N+Cabo | 2024 | 1130 | 0 | 16 | 9 | 15 |
| N+Cabo | 2025 | 1116 | 5 | 25 | 19 | 17 |
| N+Cabo | 2026 | 676 | 3 | 15 | 2 | 13 |
| N+Ipi | 2019 | 795 | 6 | 1 | 16 | 1 |
| N+Ipi | 2020 | 876 | 9 | 0 | 24 | 0 |
| N+Ipi | 2021 | 664 | 7 | 0 | 21 | 0 |
| N+Ipi | 2022 | 540 | 9 | 2 | 17 | 1 |
| N+Ipi | 2023 | 372 | 7 | 2 | 4 | 1 |
| N+Ipi | 2024 | 322 | 11 | 0 | 7 | 0 |
| N+Ipi | 2025 | 307 | 1 | 1 | 4 | 1 |
| N+Ipi | 2026 | 112 | 2 | 1 | 1 | 0 |
| P+Axi | 2019 | 56 | 0 | 0 | 1 | 0 |
| P+Axi | 2020 | 215 | 2 | 2 | 10 | 2 |
| P+Axi | 2021 | 292 | 2 | 3 | 7 | 5 |
| P+Axi | 2022 | 340 | 5 | 9 | 4 | 9 |
| P+Axi | 2023 | 267 | 4 | 4 | 2 | 3 |
| P+Axi | 2024 | 205 | 3 | 3 | 2 | 2 |
| P+Axi | 2025 | 112 | 3 | 1 | 6 | 1 |
| P+Axi | 2026 | 70 | 3 | 0 | 5 | 0 |
| P+Lenva | 2019 | 30 | 0 | 1 | 3 | 1 |
| P+Lenva | 2020 | 22 | 0 | 0 | 2 | 0 |
| P+Lenva | 2021 | 114 | 0 | 0 | 3 | 0 |
| P+Lenva | 2022 | 789 | 5 | 44 | 20 | 44 |
| P+Lenva | 2023 | 1389 | 6 | 93 | 22 | 89 |
| P+Lenva | 2024 | 892 | 6 | 61 | 18 | 62 |
| P+Lenva | 2025 | 886 | 3 | 98 | 5 | 92 |
| P+Lenva | 2026 | 418 | 2 | 34 | 18 | 34 |

Annual counts above are ancillary reconstruction counts, not treated-patient uptake. Primary annual counts are provided in S5/S16. Proteinuria components can include mixed reports and need not equal the VEGF-axis exclusive count.

| **Target window** | **Reporter country / occupation** | **Report type** | **Sender** | **Reports** | **P/N/T counts** |
| --- | --- | --- | --- | --- | --- |
| N+Cabo | CZ / PH | EXP | BRISTOL MYERS SQUIBB | 1 | 1/0/0 |
| N+Cabo | FR / MD | EXP | BRISTOL MYERS SQUIBB | 1 | 0/1/0 |
| N+Cabo | JP / MD | EXP | BRISTOL MYERS SQUIBB | 1 | 0/1/0 |
| N+Cabo | JP / MD | EXP | BRISTOL-MYERS SQUIBB COMPANY | 1 | 0/1/0 |
| N+Cabo | RO / MD | EXP | BRISTOL MYERS SQUIBB | 1 | 1/1/0 |
| N+Cabo | US / CN | PER | BRISTOL-MYERS SQUIBB COMPANY | 1 | 1/0/0 |
| P+Axi | BR / MD | EXP | BRISTOL MYERS SQUIBB | 1 | 1/0/0 |
| P+Axi | CZ / PH | EXP | BRISTOL MYERS SQUIBB | 1 | 1/0/0 |
| P+Axi | FR / MD | EXP | BRISTOL MYERS SQUIBB | 1 | 0/1/0 |
| P+Axi | JP / MD | EXP | BRISTOL MYERS SQUIBB | 1 | 0/1/0 |
| P+Axi | JP / MD | EXP | BRISTOL-MYERS SQUIBB COMPANY | 1 | 0/1/0 |
| P+Axi | RO / MD | EXP | BRISTOL MYERS SQUIBB | 1 | 1/1/0 |
| P+Axi | US / CN | PER | BRISTOL-MYERS SQUIBB COMPANY | 1 | 1/0/0 |
| P+Lenva | CZ / PH | EXP | BRISTOL MYERS SQUIBB | 1 | 1/0/0 |
| P+Lenva | FR / MD | EXP | BRISTOL MYERS SQUIBB | 1 | 0/1/0 |
| P+Lenva | JP / MD | EXP | BRISTOL MYERS SQUIBB | 1 | 0/1/0 |
| P+Lenva | JP / MD | EXP | BRISTOL-MYERS SQUIBB COMPANY | 1 | 0/1/0 |
| P+Lenva | RO / MD | EXP | BRISTOL MYERS SQUIBB | 1 | 1/1/0 |
| P+Lenva | US / CN | PER | BRISTOL-MYERS SQUIBB COMPANY | 1 | 1/0/0 |

P/N/T: proteinuria/nephrotic syndrome/renal-limited TMA. Components may co-occur. This is source-metadata review of available reconstructed comparator reports, not review of clinical narratives or histology. Repeated reports across overlapping comparison windows are intentional; rows must not be summed as independent cases.

# Supplementary Table S20. Phase III renal safety context from official label summaries

| **Trial** | **Safety population** | **Endpoint** | **Combination all / grade 3–4 %** | **Sunitinib all / grade 3–4 %** | **Interpretive limit** |
| --- | --- | --- | --- | --- | --- |
| KEYNOTE-426 | P+Axi 429; sunitinib 425 | Increased creatinine, laboratory | 43 / 4.3 | 40 / 2.4 | Serious AKI 2.3% in P+Axi; not a grade ≥3 value |
| CLEAR | P+Lenva 352; sunitinib 340 | Grouped AKI adverse reaction | 21 / 5 | 16 / 2 | Broad grouped endpoint includes creatinine/renal function terms |
| CLEAR | Same safety population | Proteinuria adverse reaction | 30 / 8 | 13 / 3 | Not a biopsy-defined endothelial endpoint |
| CheckMate 9ER | N+Cabo 320; sunitinib 320 | Increased creatinine, laboratory | 39 / 1.3 | 42 / 0.6 | Not identical to clinical AKI incidence |
| CheckMate 214 | N+Ipi 547; sunitinib 535 | Increased creatinine, laboratory | 42 / 2.1 | 46 / 1.7 | Not a head-to-head comparison with ICI–TKI combinations |

Laboratory percentages use patients with evaluable baseline and on-treatment tests, not necessarily the full safety population. KEYNOTE-426 laboratory denominators vary by test (342–425 versus 345–422); CheckMate 214 ranges are 490–538 versus 485–523. No cross-trial pooling or ranking is performed. Thresholded label tables omitting an event do not establish a zero incidence. Trial follow-up, grading, event grouping and surveillance differ from spontaneous reporting.

KEYNOTE-426 official prescribing information, accessed 16 September 2026: https://dailymed.nlm.nih.gov/dailymed/drugInfo.cfm?setid=8a903e31-936e-4ed7-8a59-59f32374f338

CLEAR official prescribing information, accessed 16 September 2026: https://dailymed.nlm.nih.gov/dailymed/fda/fdaDrugXsl.cfm?setid=f4bedd21-efde-44c6-9d9c-b48b78d7ed1e

CheckMate 9ER official prescribing information, accessed 16 September 2026: https://dailymed.nlm.nih.gov/dailymed/drugInfo.cfm?setid=3850cce2-6137-42e5-a792-d318c4a4b3b5

CheckMate 214 official prescribing information, accessed 16 September 2026: https://dailymed.awsprod.nlm.nih.gov/dailymed/drugInfo.cfm?setid=f570b9c4-6846-4de2-abfa-4d0a4ae4e394

# Supplementary Table S21. Regimen-only and feature-ablated temporal classifiers

| **Model** | **Features** | **Macro-AUPRC** | **Macro-AUROC** | **Immune-IS AP** | **VEGF-axis AP** | **AKI AP** |
| --- | --- | --- | --- | --- | --- | --- |
| M1 locked | 55 | 0.606 | 0.822 | 0.175 | 0.944 | 0.699 |
| Locked M2 reproduced | 60 | 0.638 | 0.840 | 0.253 | 0.946 | 0.717 |
| Regimen only | 5 | 0.453 | 0.678 | 0.266 | 0.773 | 0.320 |
| Remove reporting context | 46 | 0.586 | 0.781 | 0.363 | 0.883 | 0.511 |
| Remove creatinine term | 59 | 0.639 | 0.840 | 0.254 | 0.946 | 0.717 |
| Remove context + creatinine | 45 | 0.585 | 0.781 | 0.363 | 0.883 | 0.510 |
| Remove context + 3 renal-proximal terms | 43 | 0.583 | 0.779 | 0.364 | 0.881 | 0.503 |

All ablated models used the same 961 development reports and 537 temporal reports, C=0.03, l1_ratio=0, and balanced class weights. Median imputation and age scaling used development data only; no hyperparameters were retuned on the temporal set. All models converged. Original M1 and M2 metrics are provided in Table S9.

Reporting-context ablation removed seven country indicators, HCP and profession-missing flags, and five seriousness/outcome indicators (14 variables). Additional renal-proximal removal excluded blood creatinine increased, hyperkalaemia, and metabolic acidosis. Removing reporting context reduced macro-AUPRC from 0.638 to 0.586, but increased immune-interstitial AP from 0.253 to 0.363. VEGF-axis AP decreased from 0.946 to 0.883 and AKI AP from 0.717 to 0.511. These descriptive differences were not tested with paired difference intervals and were not used to replace M2. Feature sets and metrics are supplied in ML_ablation_feature_sets.csv and ML_ablation_metrics.csv.

# Supplementary Table S22. Full retained non-anchor term manifest and feature exclusions

| **No.** | **Retained term** | **Encoding** | **Use** |
| --- | --- | --- | --- |
| 1 | PT:HYPERTENSION | Binary co-report | M1 and M2 |
| 2 | PT:DIARRHOEA | Binary co-report | M1 and M2 |
| 3 | PT:PALMAR PLANTAR ERYTHRODYSAESTHESIA SYNDROME | Binary co-report | M1 and M2 |
| 4 | PT:MALIGNANT NEOPLASM PROGRESSION | Binary co-report | M1 and M2 |
| 5 | PT:HYPOTHYROIDISM | Binary co-report | M1 and M2 |
| 6 | PT:DECREASED APPETITE | Binary co-report | M1 and M2 |
| 7 | PT:FATIGUE | Binary co-report | M1 and M2 |
| 8 | PT:MALAISE | Binary co-report | M1 and M2 |
| 9 | PT:NAUSEA | Binary co-report | M1 and M2 |
| 10 | PT:PYREXIA | Binary co-report | M1 and M2 |
| 11 | PT:DEHYDRATION | Binary co-report | M1 and M2 |
| 12 | PT:PLATELET COUNT DECREASED | Binary co-report | M1 and M2 |
| 13 | PT:ANAEMIA | Binary co-report | M1 and M2 |
| 14 | PT:OEDEMA PERIPHERAL | Binary co-report | M1 and M2 |
| 15 | PT:HEPATIC FUNCTION ABNORMAL | Binary co-report | M1 and M2 |
| 16 | PT:WEIGHT DECREASED | Binary co-report | M1 and M2 |
| 17 | PT:RASH | Binary co-report | M1 and M2 |
| 18 | PT:BLOOD CREATININE INCREASED | Binary co-report | M1 and M2 |
| 19 | PT:DYSPNOEA | Binary co-report | M1 and M2 |
| 20 | PT:SEPSIS | Binary co-report | M1 and M2 |
| 21 | PT:VOMITING | Binary co-report | M1 and M2 |
| 22 | PT:HYPERKALAEMIA | Binary co-report | M1 and M2 |
| 23 | PT:STOMATITIS | Binary co-report | M1 and M2 |
| 24 | PT:DYSPHONIA | Binary co-report | M1 and M2 |
| 25 | PT:OEDEMA | Binary co-report | M1 and M2 |
| 26 | PT:ASTHENIA | Binary co-report | M1 and M2 |
| 27 | PT:PNEUMONIA | Binary co-report | M1 and M2 |
| 28 | PT:ADRENAL INSUFFICIENCY | Binary co-report | M1 and M2 |
| 29 | PT:CARDIAC FAILURE | Binary co-report | M1 and M2 |
| 30 | PT:BLOOD PRESSURE INCREASED | Binary co-report | M1 and M2 |
| 31 | PT:PLEURAL EFFUSION | Binary co-report | M1 and M2 |
| 32 | PT:OFF LABEL USE | Binary co-report | M1 and M2 |
| 33 | PT:GENERAL PHYSICAL HEALTH DETERIORATION | Binary co-report | M1 and M2 |
| 34 | PT:TASTE DISORDER | Binary co-report | M1 and M2 |
| 35 | PT:ASCITES | Binary co-report | M1 and M2 |
| 36 | PT:METABOLIC ACIDOSIS | Binary co-report | M1 and M2 |

The feature manifest (including 19 blacklist entries as written in the manifest, not 19 distinct clinical families), anchor-exclusion list, retained PT class frequencies and aggregate sparsity counts are available in the Zenodo archive. The complete sparse-term catalogue is not redistributed. Thirty-six retained PTs plus 19 static predictors form M1; five regimen indicators form M2. TTO variables remain sensitivity-only. Anchor exclusion is not proof that remaining predictors contain only biological information.

# Supplementary Table S23. READUS-PV and TRIPOD+AI reporting maps

Item-level applicability and location maps use paraphrased labels; original checklists remain authoritative. Zenodo v1.0.0: https://doi.org/10.5281/zenodo.22807359. The README and Source_Data_Index.csv describe reproduction requirements, exclusions and file locations. The study was not preregistered.

| **READUS-PV item** | **Topic** | **Location / limitation** |
| --- | --- | --- |
| 1a | Design | Title; §2.1 |
| 1b | Exposure outcome | Title; Abstract |
| 2a | Background | Introduction |
| 2b | Rationale | Introduction, final paragraphs |
| 2c | Suitability | §2.1; Discussion limitations |
| 3 | Objectives | Introduction final paragraph |
| 4a | Design | §2.1 |
| 4b | Outline | Figure 1; Methods |
| 5a | Database | §2.1; S1/S12 |
| 5b | Processing | §2.1; S1; archive code |
| 6a | Population | §2.2; Figure 1 |
| 6b | Variables | §§2.2–2.3; S2/S8 |
| 6c | Grouping | §§2.2–2.3; S2/S18 |
| 6d | Additional data | §2.9; S20 |
| 7a | Description | §2.8; Table 1 |
| 7b | Estimation | §2.4 |
| 7c | Sensitivity | §§2.4, 2.9 |
| 7d | Case assessment | No clinical adjudication; source metadata only S19 |
| 7e | Other sources | §2.7; S20 |
| 8a | Flow | Figure 1; S1/S4 |
| 8b | Characteristics | Table 1; S3 |
| 9 | Estimates | Tables 2/4; S5–S7/S13/S16–S18 |
| 10 | Case results | Source review S19; clinical narratives unavailable |
| 11 | Interpretation | Discussion; trial context S20 |
| 12a | Generalisability | Discussion |
| 12b | Clinical implications | Discussion; no treatment-selection use |
| 12c | Future research | Discussion final paragraph |
| 13 | Limitations | Discussion |
| 14a | Funding | Funding statement |
| 14b | Conflicts | Conflict statement |
| 14c | Ethics | Ethics statement |
| 14d | Access | Data availability statement; Zenodo v1.0.0: 10.5281/zenodo.22807359 |

READUS-PV checklist source: https://readus-statement.org/wp-content/uploads/2024/03/readus-checklist.pdf

| **TRIPOD+AI item** | **Topic** | **Location / applicability** |
| --- | --- | --- |
| 1 | Title | Population and reporting task named; model is secondary, not a clinical prediction claim |
| 2 | Abstract | Secondary model role, temporal n and principal performance reported |
| 3a | Context | Introduction; §2.5; not diagnostic/prognostic deployment |
| 3b | Purpose | §2.5; research users only |
| 3c | Inequalities | No subgroup fairness evaluation; reporting context limitations discussed |
| 4 | Objectives | Introduction; §2.5 |
| 5a | Sources | §2.1; S1 |
| 5b | Dates | §2.5; S4 |
| 6a | Setting | Public reporting systems; centres not identifiable |
| 6b | Eligibility | §§2.2–2.3; S4 |
| 6c | Treatments | §2.5; multi-hot regimen indicators |
| 7 | Preparation | §2.5; S8/S22; scripts |
| 8a | Labels | §2.3; proxy labels with no prediction horizon |
| 8b | Assessors | Not clinically adjudicated; deterministic labels |
| 8c | Blinding | No human blinded assessment; anchor exclusion documented |
| 9a | Selection | §2.5; S8/S22 |
| 9b | Predictors | S8/S22; co-recorded not pre-injury measurements |
| 9c | Assessors | No subjective predictor assessor; coded fields |
| 10 | Size | Available eligible reports; no formal power calculation; minority-class limitation |
| 11 | Missingness | §2.5; S3/S4; development-only imputation |
| 12a | Partitions | §2.5; S4 |
| 12b | Transforms | §2.9; manifest; model code |
| 12c | Model building | §2.5; frozen tuning grids |
| 12d | Clustering | No cluster-aware model; country/source dependence remains |
| 12e | Metrics | §2.5; S9/S10 |
| 12f | Updating | No temporal recalibration or model replacement |
| 12g | Prediction | Locked numerical parameters and portable inference code in the Zenodo archive, models/ and code/portable_model.py |
| 13 | Imbalance | Balanced weights; no synthetic oversampling; calibration limitations |
| 14 | Fairness | Not established; clinical deployment not proposed |
| 15 | Outputs | Class probabilities and argmax; mixed probabilities, no forced truth |
| 16 | Temporal differences | S1/S3/S4; missingness and class shift |
| 17 | Ethics | Ethics statement; no identifiable clinical records |
| 18a | Funding | Funding statement |
| 18b | Conflicts | Conflict statement |
| 18c | Protocol | Unregistered analysis specifications and amendments: §§2.1, 2.8; post hoc analyses: §2.9. |
| 18d | Registration | Not preregistered (§2.1); the Zenodo deposit is not a study registration. |
| 18e | Data | Zenodo v1.0.0 (10.5281/zenodo.22807359); scope and exclusions: Data availability statement and README. |
| 18f | Code | Zenodo v1.0.0 (10.5281/zenodo.22807359), code/ and models/; external-input requirements: README. |
| 19 | Public involvement | Patients and members of the public did not contribute to study design, conduct, interpretation, reporting, or dissemination planning. See manuscript involvement statement. |
| 20a | Flow | Figure 1; exact S4 |
| 20b | Description | Tables 1/S1/S3/S4; no full demographic subgroup evaluation |
| 20c | Shift | Figure 2; S2/S3; class counts and missingness |
| 21 | Analysis size | §3.1; S4/S21 |
| 22 | Specification | models/, metadata/feature_manifest_v1.0.csv and code/; numerical model parameters, not executable pickles. |
| 23a | Performance | Table 3; S9/S10; no subgroup fairness intervals |
| 23b | Heterogeneity | No cluster performance analysis; limitation retained |
| 24 | Updating | Not performed; locked model retained |
| 25 | Interpretation | Discussion; modest increment and weak immune recovery |
| 26 | Limitations | Discussion; reporting bias, proxies, missingness and small classes |
| 27a | Implementation | Not for clinical deployment |
| 27b | Users | Research-only code; no clinical interface |
| 27c | Next steps | Discussion; clinical adjudication and external clinical data needed |

TRIPOD+AI authoritative checklist and explanation: https://www.tripod-statement.org/wp-content/uploads/2024/04/TRIPODAI-Supplement.pdf; statement DOI: 10.1136/bmj-2023-078378. The checklist is used as a transparency map for a reporting-label classifier, not to imply clinical validation.

# Supplementary Table S24. Verification of references published in 2026

| **Reference** | **Final bibliographic coordinates** | **DOI** | **Status** |
| --- | --- | --- | --- |
| 28 | Front Immunol 17:1854177 | 10.3389/fimmu.2026.1854177 | Published 17 Jun 2026 |
| 31 | Cancer Immunol Immunother 75(3):84 | 10.1007/s00262-025-04288-6 | Published 25 Feb 2026 |
| 45 | Front Immunol 17:1759135 | 10.3389/fimmu.2026.1759135 | Published 18 Jun 2026 |
| 50 | Drug Saf 49(2):147–162 | 10.1007/s40264-025-01604-y | Final Feb 2026 issue; online 24 Sep 2025 |
| 51 | Front Pharmacol 17:1760208 | 10.3389/fphar.2026.1760208 | Published 20 Mar 2026 |
| 53 | PLoS One 21(1):e0340794 | 10.1371/journal.pone.0340794 | Published 9 Jan 2026 |

Reference 28 verification record: https://pubmed.ncbi.nlm.nih.gov/42389519/

Reference 31 verification record: https://pubmed.ncbi.nlm.nih.gov/41739193/

Reference 45 verification record: https://pubmed.ncbi.nlm.nih.gov/42396449/

Reference 50 verification record: https://pubmed.ncbi.nlm.nih.gov/40993470/

Reference 51 verification record: https://www.frontiersin.org/journals/pharmacology/articles/10.3389/fphar.2026.1760208/full

Reference 53 verification record: https://pubmed.ncbi.nlm.nih.gov/41511993/

Publication details were checked against PubMed and/or publisher records on 16 September 2026. Reference 50 has an indexed correction: Drug Saf 2025;48(12):1663–1664, DOI 10.1007/s40264-025-01624-8, PMID 41091354.
